# Development of Quinazolinone Derivatives as Modulators of Virulence Factors of *Pseudomonas aeruginosa* Cystic Fibrosis Strains

**DOI:** 10.3390/molecules28186535

**Published:** 2023-09-09

**Authors:** Gabriele Carullo, Giovanni Di Bonaventura, Sara Rossi, Veronica Lupetti, Valeria Tudino, Simone Brogi, Stefania Butini, Giuseppe Campiani, Sandra Gemma, Arianna Pompilio

**Affiliations:** 1 Department of Biotechnology, Chemistry and Pharmacy, University of Siena, Via Aldo Moro 2, 53100 Siena, Italy; gabriele.carullo@unisi.it (G.C.); rossi115@student.unisi.it (S.R.); valeria.tudino@unisi.it (V.T.); butini3@unisi.it (S.B.); giuseppe.campiani@unisi.it (G.C.); 2 Department of Medical, Oral and Biotechnological Sciences, School of Medicine and Health Sciences “G. d’Annunzio” University of Chieti-Pescara, Via dei Vestini 31, 66100 Chieti, Italy; gdibonaventura@unich.it (G.D.B.); veronica.lupetti@studenti.unich.it (V.L.); arianna.pompilio@unich.it (A.P.); 3 Department of Pharmacy, University of Pisa, Via Bonanno 6, 56126 Pisa, Italy; simone.brogi@unipi.it

**Keywords:** *Pseudomonas aeruginosa*, virulence factors, biofilm, quorum sensing, PqsR, cystic fibrosis, antimicrobial resistance, quinazolinone

## Abstract

*Pseudomonas aeruginosa* (PA), one of the ESKAPE pathogens, is an opportunistic Gram-negative bacterium responsible for nosocomial infections in humans but also for infections in patients affected by AIDS, cancer, or cystic fibrosis (CF). Treatment of PA infections in CF patients is a global healthcare problem due to the ability of PA to gain antibiotic tolerance through biofilm formation. Anti-virulence compounds represent a promising approach as adjuvant therapy, which could reduce or eliminate the pathogenicity of PA without impacting its growth. Pyocyanin is one of the virulence factors whose production is modulated by the *Pseudomonas* quinolone signal (PQS) through its receptor PqsR. Different PqsR modulators have been synthesized over the years, highlighting this new powerful therapeutic strategy. Based on the promising structure of quinazolin-4(3*H*)-one, we developed compounds **7a**–**d**, **8a**,**b**, **9**, **10**, and **11a**–**f** able to reduce biofilm formation and the production of virulence factors (pyocyanin and pyoverdine) at 50 µM in two PA strains responsible for CF acute and chronic infections. The developed compounds did not reduce the cell viability of IB3-1 bronchial CF cells, and computational studies confirmed the potential ability of novel compounds to act as potential Pqs system modulators.

## 1. Introduction

**Figure 1:**
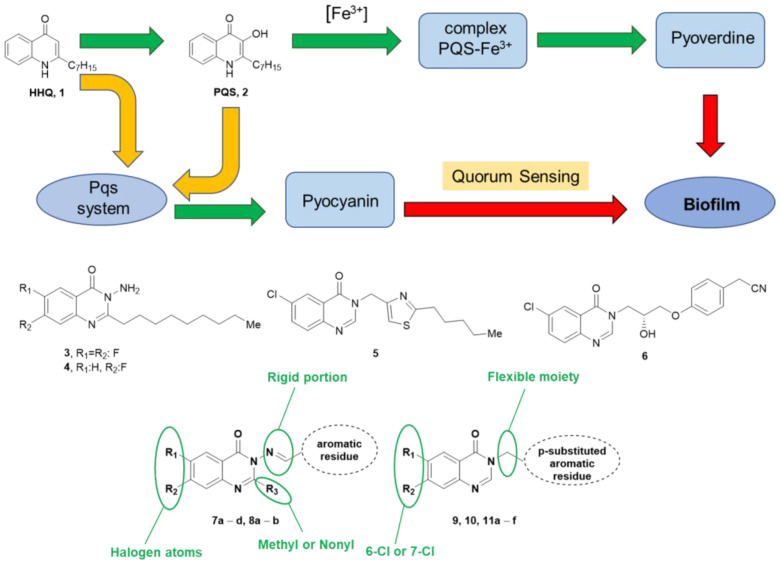
Design of new Pqs system modulators. Pqs involvement in biofilm formation mediated by virulence factors pyocyanin and pyoverdine; known Pqs system modulators and strategy for developing new modulators.

In 2008, the World Health Organization identified six multidrug-resistant and virulent bacterial pathogens grouped under the acronym ESKAPE: *Enterococcus faecium*, *Staphylococcus aureus*, *Klebsiella pneumoniae*, *Acinetobacter baumannii*, *Pseudomonas aeruginosa* (PA), and *Enterobacter* spp. These pathogens are common causes of life-threatening nosocomial infections and have become increasingly resistant to commonly used antibiotics [1]. Among the ESKAPE bacteria, PA is a Gram-negative opportunistic pathogen in immunocompromised patients affected by cancer and AIDS, but also in burn victims or patients under mechanical ventilation. The major challenge of PA infection treatment is due to the ability of PA to become resistant to antibiotics [2]. Therefore, there is a need for alternative treatment options that can effectively combat PA infections without contributing to antibiotic resistance. Colistin and tobramycin are among the antibiotics commonly considered for the first-line treatment of PA. Patients treated with these antibiotics generally present a reduction in the bacterial load, but eradication is difficult [3]. Infection caused by PA is particularly challenging in patients affected by cystic fibrosis (CF), a lung disease characterized by inflammation, lung damage, and ultimately, respiratory failure [4,5]. PA is intrinsically resistant to several antimicrobials due to its restricted outer membrane permeability, the expression of inducible cephalosporinases, and other mechanisms. It can also quickly acquire antibiotic resistance via chromosomal changes or horizontal gene acquisition. PA can also gain antibiotic tolerance by producing biofilms, surface-attached, structured microbial consortia encased in a self-produced extracellular matrix. For example, chronic pulmonary infections in CF patients are associated with bacterial biofilms coated in a matrix consisting of polysaccharide alginate, proteins, and DNA, which render arduous eradication and lead to chronic inflammation of the tissues, exacerbating the fibrosis [6]. To overcome the selection of multi- or even pan-resistant PA strains, an unavoidable problem associated with the administration of bacteriostatic or bactericidal agents, a promising treatment approach could be the design of anti-virulence compounds, which could reduce or eliminate the pathogenicity of bacteria without impacting their growth [7]. Compared to conventional antibiotic therapy, it would put less selection pressure on bacterial survival, lowering the risk of developing drug resistance. In monotherapy, non-bactericidal anti-virulence drugs may make pathogens more sensitive to the actions of the host immune system. Moreover, the host commensal flora would not be affected. By preventing the development of biofilms’ efficient barrier, anti-virulence compounds could restore the effectiveness of current antibiotics when used in combination therapy. Noteworthy, PA has a considerable genome of about 6000 genes, some of which are associated with the expression of virulence factors. PA often uses the cell density-dependent communication system named quorum sensing (QS) to control virulence factor production, motility, and biofilm formation [8]. Among the virulence traits produced during the infection, which include motility factors and biofilm formation [9], pyocyanin is a redox-active molecule capable of cytotoxic effects against mammalian cells, while pyoverdine is a siderophore produced by PA under low iron concentrations. In this context, interfering with the QS system is an appealing and highly investigated strategy to overcome PA pathogenicity and resistance. The principal genes involved in PA QS are *lasI/R*, *rhlI/R*, and *pqs*, which regulate the delivery of elastase, protease, exotoxin A, rhamnolipids, hydrogen cyanide, lipase, and pyocyanin under a transcriptional regulation mediated by the proteins LasR, RhlR, or PqsR [10]. PqsR uses two auto-inducers to favor communication among bacteria that are divided in the alkyl quinolones family, namely 2-heptyl-4-hydroxyquinoline (HHQ, **1**, Figure 1) and 3,4-dihydroxy-2-heptylquinoline (*Pseudomonas* quinolone signal, PQS, **2**, Figure 1). Their binding to PqsR promotes the positive regulation of different genes, including the *pqsABCDE* operon*,* which controls genes involved in protein translation, secretion, response to oxidative stress, and the production of different virulence factors such as pyocyanin and biofilm [11,12]. Alkyl quinolone modulation targeting the Pqs system has emerged as a promising anti-virulence strategy. Different 3-hydroxy-pyridin-4(1*H*)- and pyrimidine-based derivatives have been developed as PqsR antagonists or inverse agonists, showing biofilm inhibitory activity via the Pqs system [13,14,15]. In a medicinal chemistry effort, the quinolone structure of PQS was switched to a quinazolinone core, obtaining compounds **3** and **4** (Figure 1), which showed PqsR antagonism beyond biofilm inhibition and pyocyanin reduction in the laboratory PAO1 strain [16]. Further structure–activity relationship (SAR) studies revealed compounds **5** and **6** (Figure 1) as promising tools to obtain Pqs system modulators that could act as Pqs system conquerors [17,18]. In particular, **6** reduced levels of virulence factors in both PAO1 and PA14 laboratory strains and the PAK6085 clinical isolate. Pqs system modulators developed so far displayed significant variation in potency in phenotypical assays based on the strain used. So, to select hit compounds that interfere with critical processes of PA virulence in CF patients, we undertook a phenotypic-based screening against the PA clinical strains. In particular, we evaluated the effect of the synthesized compounds on biofilm and pigment (pyocyanin and pyoverdine) production. The library was built upon the privileged quinazolin-4(3*H*)-one scaffold known to interact with PqsR, decorated with substituents at N3, potentially able to fill the B-pocket of the target protein (Figure 1). In this work, we report on our phenotypical investigation of two groups of compounds, namely the 2-methyl- or 2-nonyl-quinazolin-4(3*H*)-one based hydrazones **7a**–**d** and **8a**,**b**, and *N*-benzyl substituted quinazolin-4(3*H*)-one compounds **9**, **10**, and **11a**–**f** (Figure 1). The interference of these compounds with the Pqs system has been investigated phenotypically using two PA clinical strains representative of acute (BJ5325 strain) and chronic (RP73) CF lung infections instead of laboratory reference strains, which might have lost important pathophysiological characteristics and therefore might be inadequate to document “real-world” pathogenesis. The most interesting hit compounds, tested at sub-inhibitory concentrations, showed anti-biofilm activity and lowered pyocyanin and pyoverdine levels.

## 2. Results and Discussion

### 2.1. Compounds’ Synthesis

**Scheme 1:**
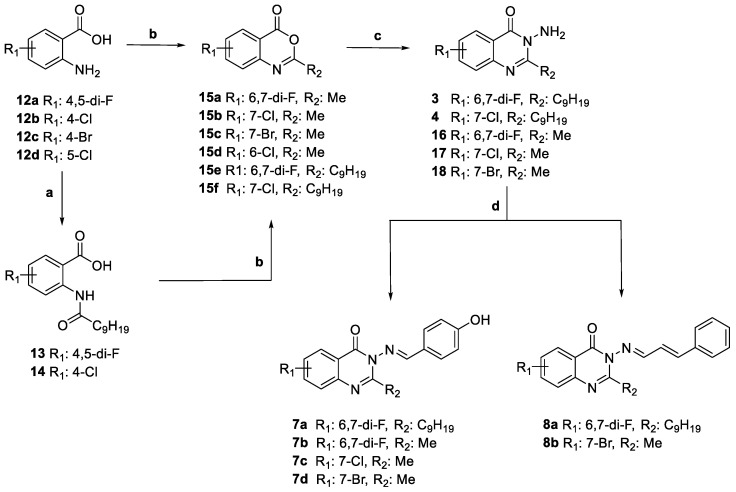
Synthesis of compounds **3**, **4**, **7a**–**d**, **8a**,**b.** Reagents and conditions: (a) decanoyl chloride, dry pyridine, 25 °C, 24 h; (b) acetic anhydride, 140 °C, 2 h; (c) hydrazine hydrate, ethanol, 80 °C, 15 h; (d) 4-hydroxybenzaldehyde (for **7a**–**d**) or (E)-cinnamaldehyde (for **8a**,**b**), acetic acid, ethanol, 80 °C, 20 h.

The synthesis of quinazolin-4(3*H*)-ones **3**, **4**, **7a**–**d**, and **8a**,**b** was realized starting from the corresponding halo-anthranilic acids **12a**–**d**, as depicted in Scheme 1. For the synthesis of lactones **15a**–**d**, the anthranilic acids were refluxed in acetic anhydride, while for the synthesis of **15e**–**f**, decanoyl chloride was first added to a solution of the corresponding anthranilic acids **12a**,**b** in dry pyridine to obtain the acyl intermediates **13** and **14** respectively, which then furnished **15e**–**f** after treatment with acetic anhydride. Lactones **15a**–**f** were treated with hydrazine hydrate in ethanol to obtain different 3-aminoquinazolin-4(3*H*)-ones **3**, **4**, and **16**–**18**. When **3** and **16**–**18** were treated with 4-hydroxybenzaldehyde in ethanol and acetic acid, the title compounds **7a**–**d** were obtained. When **3** and **18** were treated with (*E*)-cinnamaldehyde in the same conditions, **8a** and **8b** were isolated. The obtained hydrazone-based compounds **7a**–**d** and **8a**,**b** were obtained in the E_(C=N)_ geometrical configuration. According to the literature data, this configuration is the most thermodynamically stable compared to Z_(C=N)_ which is considered the short-lived isomeric form due to steric crowding which leads to a relatively less stability than E_(C=N)_ form [19,20].

**Scheme 2:**
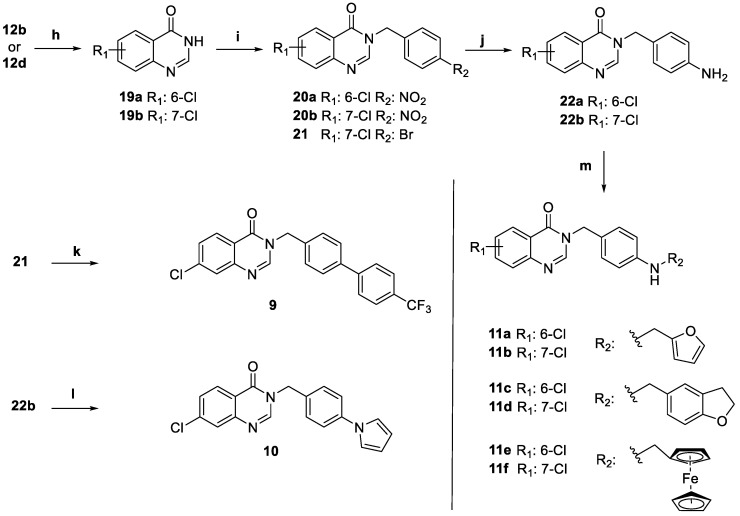
**Synthesis of compounds 9**, **10**, and **11a**–**f**. Reagents and conditions: (h) formamide, 150 °C, 20 h; (i) 4-nitrobenzyl chloride (for **20a**,**b**) or 4-bromobenzyl bromide (for **21**), potassium carbonate, sodium iodide, acetone, 50 °C, 24 h; (j) iron powder, aqueous ammonium chloride (s.s.), ethanol, 70 °C, 2 h; (k) (4′-(trifluoromethyl)-[1,1′-biphenyl]-4-yl)boronic acid, sodium carbonate, palladium, 1,2-diethoxyethane/ethanol (1:1), 80 °C, 12 h; (l) 2,5-dimethoxytetrahydrofuran, 4-chloropyridine hydrochloride, 1,4-dioxane, 100 °C, 2 h; (m) i: furfural (for **11a**,**b**), 2,3-dihydrobenzofuran-5-carbaldehyde (for **11c**–**d**), ferrocene-2-carboxaldehyde (for **11e**–**f**), acetic acid, methanol, 50 °C, 12 h, ii: sodium cyanoborohydride, 25 °C, 2 h.

The synthesis of title compounds **9**, **10**, and **11a**–**f** is depicted in Scheme 2. After heating in formamide, the anthranilic acids **12b** and **12d** were first converted into lactams **19a**,**b**. These were then alkylated in the presence of 4-nitrobenzyl chloride to obtain **20a**,**b**. Instead, **19b** was alkylated in the presence of 4-bromobenzyl bromide to obtain **21**, which was converted into the final compound **9** under Suzuki conditions using the commercially available (4′-(trifluoromethyl)-[1,1′-biphenyl]-4-yl)boronic acid. Intermediates **20a**,**b** were then reduced to anilines **22a**,**b**. Aniline **22b** was converted into final compound **10** under Clauson–Kaas conditions. Through reductive amination conditions, anilines **22a**,**b** were converted into the final compounds by treating with the corresponding aldehydes furfural (**11a**,**b**), 2,3-dihydrobenzofuran-5-carbaldehyde (**11c**–**d**), and ferrocene-2-carboxaldehyde (**11e**–**f**).

### 2.2. Antivirulence Activity

The inhibitory potential of the compounds towards PA strains was tested by the broth microdilution method. Susceptibility assays showed that all compounds have MIC values > 50 µM, thus excluding that the mechanism of action underlying the anti-virulence effects observed is related to the antibacterial activity.

#### 2.2.1. Antibiofilm Activity and SAR Study

**Figure 2:**
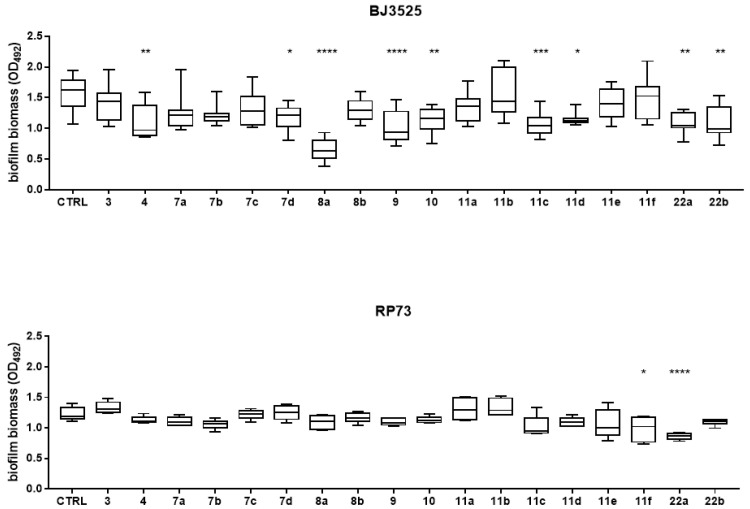
**In vitro activity compounds 3**, **4**, **7a**–**d**, **8a**,**b**, **9**, **10**, **11a**–**f**, and **22a**,**b against biofilm formation by RP73 and BJ3525 PA strains**. All molecules were tested at 50 µM under «CF-like» conditions (artificial sputum medium, 5% CO_2_, pH 6.8). Results are shown as box and whisker: the ends of the whiskers represent the minima and the maxima of all the data; the box always extends from the 5th to the 95th percentiles, while the line in the middle of the box is plotted at the median. Statistical significance at ordinary one-way ANOVA + Holm–Sidak’s multiple comparisons test: * *p* < 0.05, ** *p* < 0.01, *** *p* < 0.001, and **** *p* < 0.0001 compared to unexposed sample (control, CTRL).

The inhibitory effects of compounds **3**, **4** (reference acylhydrazide-based compounds), **7a**–**d**, **8a**,**b** (acylhydrazone-based compounds) **9**, **10**, **11a**–**f**, and intermediates **22a**,**b** (lactam-based compounds) were tested for the ability to interfere with biofilm formation at a sub-inhibitory concentration of 50 µM, and the results are shown in Figure 2. To this aim, the biofilm biomass formed by PA BJ3525 and RP73 strains was measured by the crystal violet assay staining in both cell and extracellular polymeric substances. The first series of hydrazone-based derivatives (**7a**–**d** and **8a**,**b**, Figure 1, Scheme 1) was developed based on compounds **3** and **4**, previously reported as promising Pqs system modulators [16]. In particular, compounds **7a**–**d** and **8a**,**b** are characterized by a conjugated hydrazone substituent with linkers of different lengths coupled to either a methyl or a nonyl chain at C2 of the quinazolin-4(3*H*)-one structure (Figure 1). Based on the obtained results, compounds **7d** and **8a** significantly affected the biofilm formation (reduction rate: 25%, *p* < 0.05, and 58%, *p* < 0.0001, respectively) at the concentration tested against the first-infection PA BJ3525 strain (Figure 2). To remove either the bromine substituent or the nonyl chain responsible for the low solubility of the identified compounds, we decided to further investigate the quinazolinone scaffold of reference compounds **5** and **6**. In particular, we explored the effect of the 6-Cl or 7-Cl substituents coupled to p-substituted benzyl rings at the N3 (**22a**,**b**, **9**, **10**, **11a**–**f**, Scheme 2).

Concerning the PA BJ3525 strain, compound **8a** remained the most active, causing a reduction rate of 58.4%, significantly higher than **22a**,**b** (30% and 30.2%, *p* < 0.05, respectively) bearing a small -NH_2_ substituent but also if compared with **11c** (32%, *p* < 0.001) and **11d** (26.7%, *p* < 0.01), and **10** (27.5%, *p* < 0.01). Conversely, compound **8a** showed a comparable reduction rate with **9** (34.5%) characterized by the presence of a rigid biphenyl system as the N3 substituent. Based on the obtained results, two compounds significantly affected the biofilm formation by the RP73-persistent strain, at comparable levels. Specifically, **11f** (7-chloro-substituted lactam with a pendant ferrocene at the *p*-position of the benzyl ring at N3 position) and **22a** (6-chloro-substituted lactam with a free NH_2_ at the *p*-position of the benzyl ring at N3 position), respectively, caused a reduction of 19.6% (*p* < 0.05) and 29.9% (*p* < 0.0001) compared to the unexposed control. Our findings notably indicated that **22a** exhibits an anti-biofilm effect regardless of the tested strain, while other compounds showed strain-dependent activity. Different effects of biofilm inhibitors on different PA phenotypes such as in lab and clinical strains have been observed in previous studies [17] and could be ascribed to different reasons (efflux pumps, operon activity, Pqs sequence differences). In this study, we observed that the RP73 strain showed lower susceptibility than the BJ3525 strain to the anti-biofilm activity of the tested compounds.

#### 2.2.2. Activity against Pigment Production

**Figure 3:**
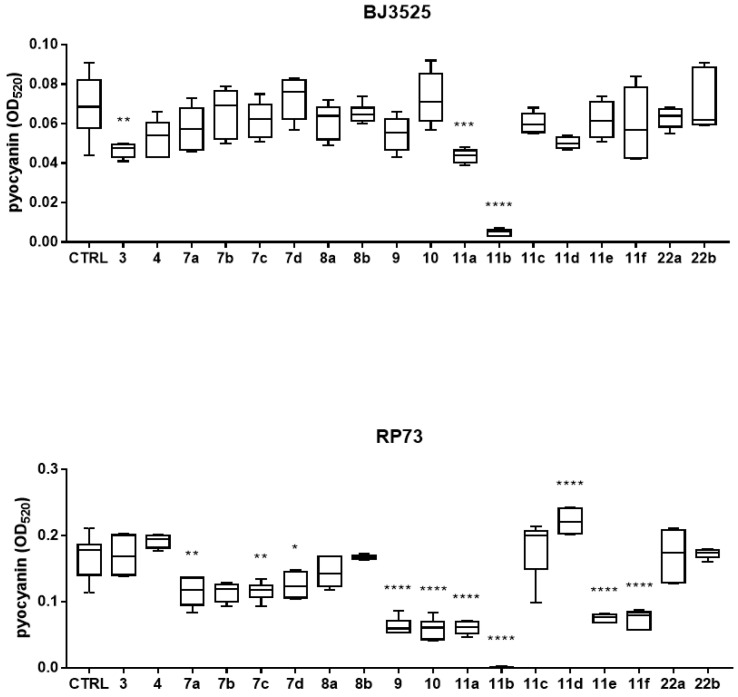
**In vitro effect of compounds 3**, **4**, **7a**–**d**, **8a**,**b**, **9**, **10**, **11a**–**f**, and **22a**,**b on pyocyanin production by BJ3525 and RP73 PA strains**. All compounds were tested at 50 µM in LB medium under an aerobic atmosphere. Results are shown as box and whisker: the ends of the whiskers represent the minima and maxima of all the data; the box always extends from the 5th to the 95th percentiles, while the line in the middle of the box is plotted at the median. Statistical significance at ordinary one-way ANOVA + Tukey’s multiple comparisons test: * *p* < 0.05, ** *p* < 0.01, *** *p* < 0.001, and **** *p* < 0.0001 compared to unexposed sample (control, CTRL).

**Figure 4:**
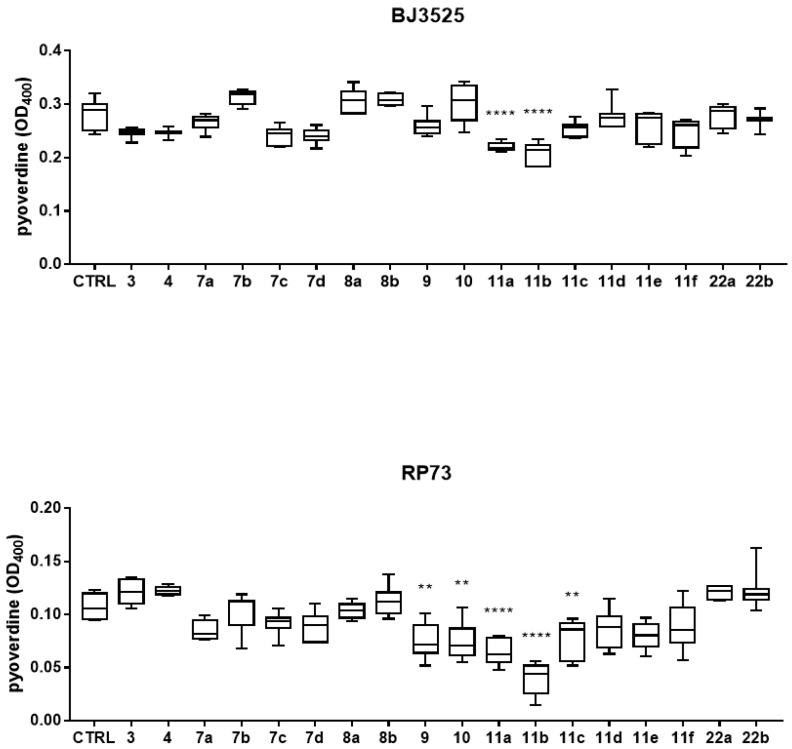
In vitro effect of compounds **3**, **4**, **7a**–**d**, **8a**,**b**, **9**, **10**, **11a**–**f**, and **22a**,**b** on pyoverdine production by BJ3525 and RP73 PA strains. All compounds were tested at 50 µM in LB medium under an aerobic atmosphere. Results are shown as box and whisker: the ends of the whiskers represent the minima and maxima of all the data; the box always extends from the 5th to the 95th percentiles, while the line in the middle of the box is plotted at the median. Statistical significance at ordinary one-way ANOVA + Tukey’s multiple comparisons test: ** *p* < 0.01 and **** *p* < 0.0001 compared to unexposed sample (control, CTRL).

Based on the results of the activity against biofilm, the effect of exposure to compounds **7a**–**d**, **8a**,**b**, **9**, **10**, **11a**–**f**, and **22a**,**b** along with the literature compounds **3** and **4** on the production of pyocyanin and pyoverdine by both PA strains was evaluated spectrophotometrically, and the results are shown in Figure 3 and Figure 4. RP73 was more sensitive to interference with pyocyanin production by the tested compounds (Figure 3). Pyocyanin production by the RP73 strain was significantly decreased by compounds **7a**,**c**,**d** (bearing a phenol substituent), **9**, **10**, and **11a**,**b**, although to different extents (pyocyanin reduction, range: 23.7–99.5%). Compound **11b** was the most effective, causing a nearly complete suppression of pyocyanin formation (99.5% vs. control, *p* < 0.0001). Conversely, exposure to **11d** significantly increased pigment formation by 35.3% compared to the control. In the case of BJ3525 strain, pyocyanin production was significantly reduced by only two of the new compounds, with **11b** as the most active, causing a significantly higher reduction rate compared to the structurally related analog **11a** (92.2% vs. 35.7%; *p* < 0.0001) (Figure 3). The effect on pyoverdine production, another important virulence factor produced by PA and involved in iron uptake, was also evaluated, and results are reported in Figure 4.

Compound **11b** was the most effective against the RP73 strain, causing a 58.3% reduction, significantly higher than that of other active compounds: **11a** (40%; *p* < 0.05), **10** (30.5%, *p* < 0.0001), **9** (29.3%, *p* < 0.0001), and **11c** (28.5%, *p* < 0.0001). Regarding the BJ3525 strain, derivatives **11a** and **11b** significantly affected pyoverdine production in a comparable way, causing a reduction rate of 21.5% and 25.6%, respectively. Overall, these findings indicated **11a** and **11b** as the most effective compounds, being able to affect pigment production in both PA strains. We also tested reference compounds **3** and **4** previously published as potent Pqs system modulators [16]. In the PAO1 strain, compound **4** was reported to significantly reduce pyocyanin levels at 100 µM, while in our study, it caused a reduction at 50 µM, although not statistically significant, of 23.7% only in the BJ3525 strain (Figure 3). Compound **4** was also effective against biofilm formation by BJ3525 (Figure 2).

### 2.3. Cytotoxic Effects

**Figure 5:**
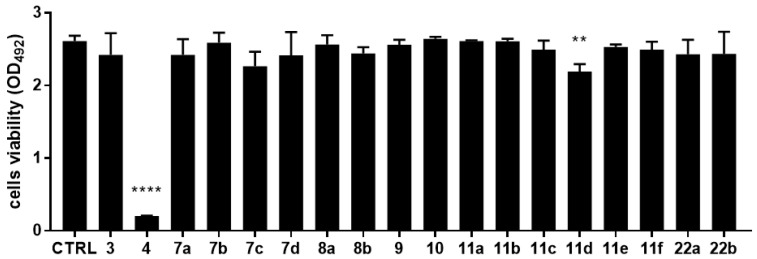
**In vitro cytotoxicity of 3**, **4**, **7a**–**d**, **8a**,**b**, **9**, **10**, **11a**–**f**, and **22a**,**b compounds against IB3-1 cells**. IB3-1 monolayers were exposed for 24 h to each compound at 50 µM. The cell viability was then measured by an MTS tetrazolium-based colorimetric assay and expressed as mean + SD absorbance at 492 nm. Statistical significance at one-way ANOVA + Holm–Sidak’s multiple comparisons post-test: ** *p* < 0.01 and **** *p* < 0.0001 vs. untreated cells (control, CTRL).

The cytotoxic potential of each compound was investigated using a cell-based MTS assay. Monolayers of IB3-1 bronchial CF cells were exposed to each compound at 50 µM, and the results are summarized in Figure 5. MTS tetrazolium-based colorimetric assay showed that all compounds tested were not toxic for IB3-1 cells, except for **4** and **11d**, which reduced cell viability by 92.3% (*p* < 0.0001) and 16.1% (*p* < 0.01), respectively, compared with untreated control cells.

### 2.4. Molecular Modeling

Identifying compounds active in phenotypic assays is an essential pre-requisite to identify molecular scaffolds worth further optimization. With the above data in our hands, we performed molecular modeling studies to set up a strategy for the future optimization of this class of derivatives.

**Figure 6:**
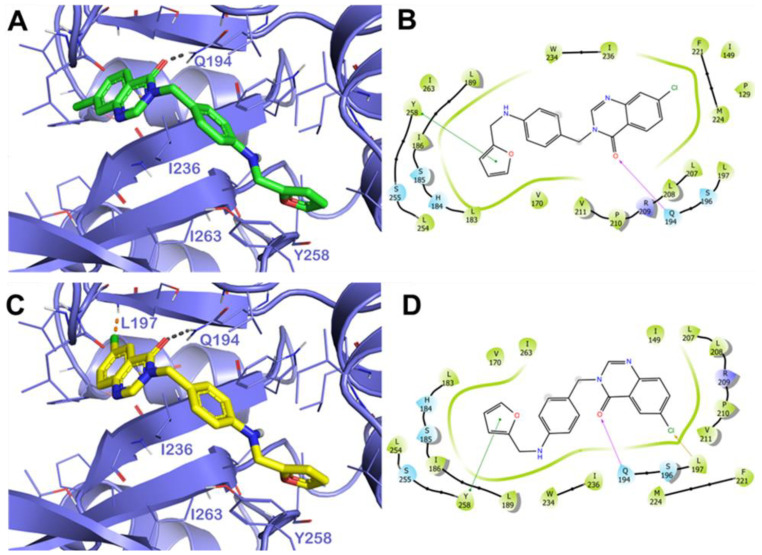
**Docking simulation for compounds 11a**,**b on PqsR active site**. (**A**) Docking output of compound **11b** (green sticks) and (**C**) of **11a** (yellow sticks) within the binding site of PqsR (PDB ID 6B8A, light blue cartoon). Lines represent the key interacting residues. The H bonds are depicted as gray dotted lines, while orange dotted lines represent the halogen bonds. Non-polar hydrogen atoms were removed for the sake of clarity. (**B**,**D**) Two-dimensional representation of the contacts established by **11b** and **11a**, respectively, within the mentioned binding site. Pictures were prepared by PyMOL and the ligand interaction diagram application was implemented in Maestro.

Accordingly, the most interesting compounds, **9** and **10**, displaying notable antibiofilm activity in the BJ3525 strain and diminishing pyocyanin/pyoverdine production in the RP73 strain, along with **11a**,**b**, affecting pyocyanin and pyoverdine production by the greatest extent, were submitted to molecular modeling studies on the PqsR as a putative target of their activity. Assessing whether the compounds directly bind to the protein target could offer valuable insights into their behavior, helping to better understand the interactions established by the selected compounds within the binding site on PqsR, and to rationalize the observed activities. With this aim, we conducted a comprehensive computer-based investigation based on molecular docking coupled to 200 ns of molecular dynamics simulation (MD) in explicit solvent. We observed a similar interaction network among the examined compounds that established several contacts with the key residues within the selected binding site [16,21,22,23,24]. In particular, for compound **11b**, we detected an H bond with the sidechain of Q194 and a π-π stacking with the residue Y258. Furthermore, a strong network of hydrophobic interactions was observed with residues I236 and I263 (Figure 6A,B). This binding mode accounted for a docking score of −8.48 kcal/mol, indicating a significant affinity of the compound for the selected binding site.

**Figure 7:**
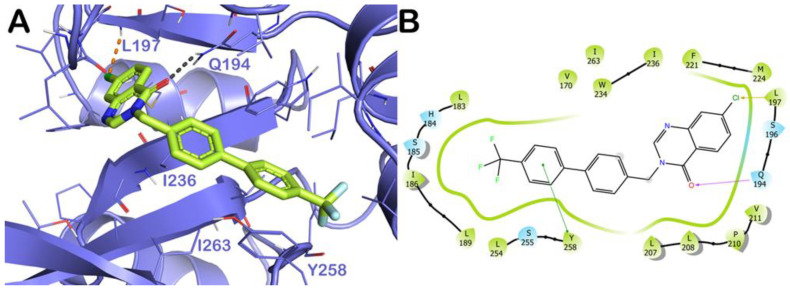
**Docking simulation for compound 9 on PqsR active site**. (**A**) Docking output of compound **9** (light yellow sticks) within the binding site of PqsR (PDB ID 6B8A, light blue cartoon). Lines represent the key interacting residues. The H bonds are depicted as gray dotted lines, while orange dotted lines represent the halogen bonds. Non-polar hydrogen atoms were removed for the sake of clarity. (**B**) Two-dimensional representation of the contacts established by **9** within the mentioned binding site. Pictures were prepared by PyMOL and the ligand interaction diagram application was implemented in Maestro.

The output of molecular docking calculation for compound **11a** (Figure 6C,D) is similar to **11b** with the difference of a potential halogen bond with the backbone of residue L197. This binding mode accounted for a docking score comparable to that found for compound **11b**, of −8.24 kcal/mol. Similarly, for compound **11a**, derivative **9** established polar and hydrophobic contacts with pivotal residues within the PsqR of the PA binding site. Compound **9** can target Y258 (π-π stacking), Q194 (H bond), and L197 (halogen-bond) with additional hydrophobic interactions with the residues I236 and I263 (Figure 7A,B). The docking score for this binding mode is close to that of the previous derivatives (−8.78 kcal/mol).

**Figure 8:**
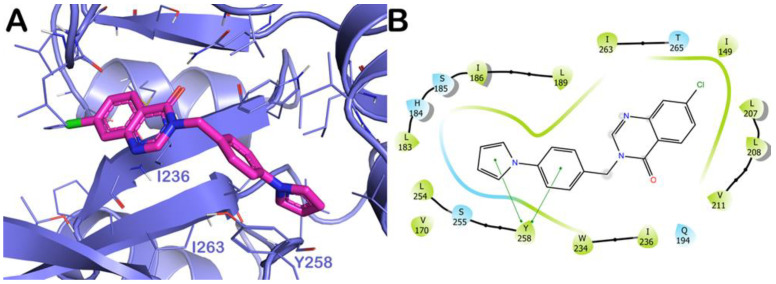
**Docking simulation for compound 10 on PqsR active site** (**A**) Docking output of compound **10** (magenta sticks) within the binding site of PqsR (PDB ID 6B8A, light blue cartoon). Lines represent the key interacting residues. Non-polar hydrogen atoms were removed for the sake of clarity. (**B**) Two-dimensional representation of the contacts established by **10** within the mentioned binding site. Pictures were prepared by PyMOL and the ligand interaction diagram application was implemented in Maestro.

The last compound examined was the derivative **10**. In this case, this compound’s different arrangement of chemical structure was reflected in a slightly different interaction pattern with respect to the other derivatives discussed previously. In particular, the contacts with Q194 and L197 were no longer detected, due to the distance over the 3.0 Å between the mentioned residues and the fused ring of the compound **10**. On the contrary, the compound was able to strongly target Y258 establishing a double π-π stacking, in addition to the hydrophobic interactions with the residues I236 and I263 (Figure 8). A docking score of −7.88 kcal/mol was observed in line with the reduced number of contacts with key residues of the binding site.

**Figure 9:**
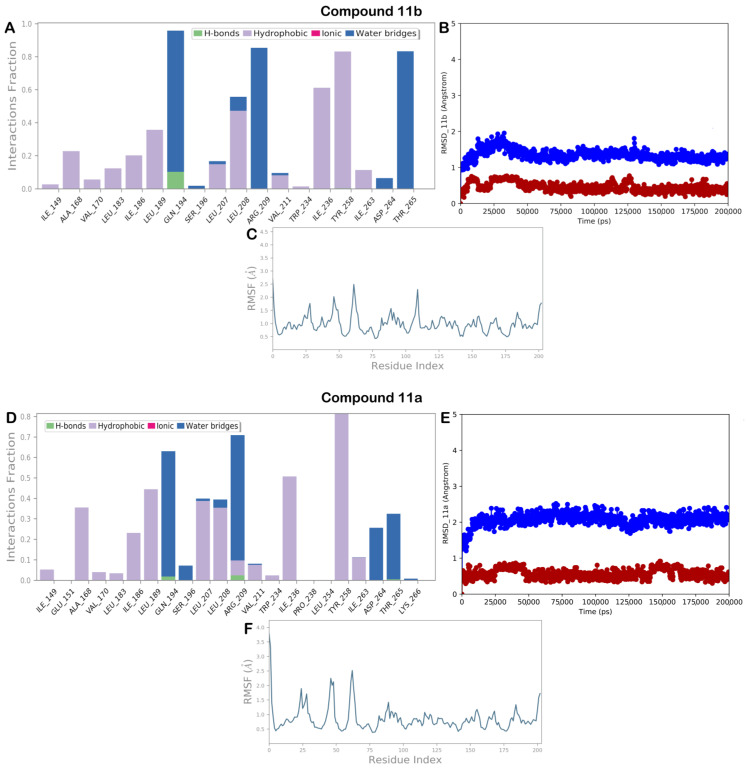
(**A**) 11b monitored during the MD run; (**B**) 11b RMSD calculation for each complex investigated in this study: protein (blue line) and ligand (red line); (**C**) RMSF calculation for 11b after 200 ns of MD simulation; (**D**) 11a monitored during the MD run; (**E**) 11a RMSD calculation for each complex investigated in this study: protein (blue line) and ligand (red line); (**F**) RMSF calculation for 11a after 200 ns of MD simulation. The interactions can be grouped into four types: H bonds (green), hydrophobic (gray), ionic (magenta), and water bridges (blue).

**Figure 10:**
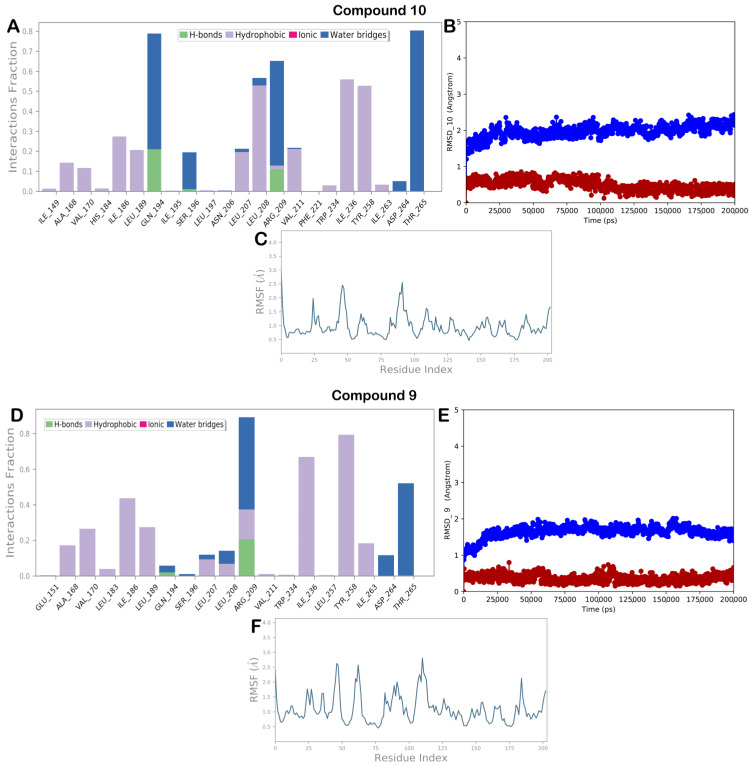
Molecular dynamics for compounds 9, (**A**) 10 monitored during the MD run; (**B**) 10 RMSD calculation for each complex investigated in this study: protein (blue line) and ligand (red line); (**C**) RMSF calculation for 10 after 200 ns of MD simulation; (**D**) 9 monitored during the MD run; (**E**) 9 RMSD calculation for each complex investigated in this study: protein (blue line) and ligand (red line); (**F**) RMSF calculation for 9 after 200 ns of MD simulation. The interactions can be grouped into four types: H bonds (green), hydrophobic (gray), ionic (magenta), and water bridges (blue).

To validate the docking output, we investigated the behavior of the selected compound within the selected binding site, performing 200 ns of the MD simulation. The output of the timeline of interactions, along with the calculation of RMSD and RMSF, is reported in Figure 9 and Figure 10. As depicted in Figure 9A, compound **11b** maintained the same binding mode found by molecular docking studies. In fact, the interaction with Q194 is still detectable, although sometimes it becomes water mediated. Moreover, the strong hydrophobic network of interaction with Y258 is nicely maintained, and in addition, we observed some other hydrophobic interactions (L189, L208, and I236) and water-mediated H bonds (R209 and T265) that can contribute to stabilizing the binding mode previously discussed. This occurrence is in line with the high stability of the system found, with no expansion or contraction of the investigated complex (Figure 9B,C). The output of the molecular MD simulation for compound **11a** is illustrated in Figure 9D. As discussed previously for compound **11b**, a similar trend was observed for compound **11a** (Figure 9D). In fact, also in this case, the contacts found by molecular docking were maintained, and similar additional hydrophobic contacts (L189, L207, L208, and I236) and water-mediated H bonds (R209, D264, and T265) were observed with slight differences. Furthermore, this investigated system also showed high stability and small fluctuations, as illustrated in Figure 9E,F by calculating RMSD and RMSF.

The MD simulation output for compound **10**, also in this case, highlighted a conservation of the main contacts found by molecular docking. As reported in Figure 10A, the contacts with Q194 and Y258 were maintained while the halogen bond with the backbone of L197 became sporadic. In addition, hydrophobic contacts (L208 and I236) and water-mediated H bonds (R209 and T265) can contribute to stabilizing the described binding mode. Furthermore, as reported in Figure 10B,C, the complex PqsR/**10** showed low RMSD and RMSF, indicating the system was stable during the 200 ns of the MD simulation.

Finally, MD simulation studies for compound **9**, although the derivatives showed a slightly different arrangement, showed a similar pattern of interaction as previously described (Figure 10D), and also, in this case, the stability of the biological system is significant as highlighted by the calculation of RMSD and RMSF (Figure 10E,F).

## 3. Materials and Methods

### 3.1. General Chemistry Methods

All the reagents used were commercially available and purchased from Merck (Milan, Italy). The reaction progress was monitored by TLC using silica gel 60 F254 (0.040−0.063 mm) with detection by UV (254 nm). ^1^H NMR and ^13^C NMR spectra were recorded on a Varian 300 MHz spectrometer or Bruker 400 MHz spectrometer using the residual signal of the deuterated solvent as the internal standard. Splitting patterns are described as singlet (s), doublet (d), triplet (t), and quartet (q); the value of chemical shifts (δ) is given in ppm, and coupling constants (*J*) are shown in hertz (Hz). Electrospray ionization mass spectrometry (ESI-MS) spectra were performed by an Agilent 1100 series LC/MSD spectrometer. HPLC/MS analyses were performed with the LC/MSD preparative system Agilent 1260 Infinity II single quadrupole (LC/MSDiQ), connected to a UV detector (254 nm) using an InfinityLab Poroshell 120 EC-18 column (2.1 × 50 mm^2^, 2.7 µm) and a second InfinityLab Poroshell 120 EC-C18 column (4.6 × 100 mm^2^, 2.7 µm). For the ESI ionization, N_2_ was used as drying gas flow (9 mL/min, temperature 350 °C, atomizing pressure 40 PSI). ESI-HRMS spectra were acquired by a linear ion-trap-Orbitrap hybrid mass spectrometer (LTQ Orbitrap XL) (Thermo Fisher Scientific, Bremen, Germany) operating in positive electrospray ionization mode. Data were collected and analyzed using the Xcalibur 2.2 software provided by the manufacturer (Chemical Compounds Characterization: Supplementary Information).

*General procedure for the synthesis of* **13**–**14**. To a solution of anthranilic acid (**12a** or **12b**) (500 mg, 1 eq.) in dry pyridine (5 mL), decanoyl chloride (850 µL, 1.5 eq.) was dropped. The reaction mixture was stirred at 25 °C for 24 h. Then, the reaction was warmed to room temperature (RT) and treated with 15 mL of 5% HCl. The mixture was then partitioned between H_2_O and EtOAc (3 × 20 mL). The combined organics were dried, filtered, and evaporated under reduced pressure.
*2-Decanamido-4,5-difluorobenzoic acid* (**13**). Pale brown solid, 76% yield. Spectroscopic data are in agreement with those reported [25].
*4-Chloro-2-decanamidobenzoic acid* (**14**). White solid, 91% yield. Spectroscopic data are in agreement with those reported [16].
*General procedure for the synthesis of compounds* **15a**–**f**. Anthranilic acids **12a**–**d** or substituted anthranilic acids **13**–**14** (500 mg, 1 eq.) were heated at 140 °C in acetic anhydride (2.8 mL, 10 eq.) for 2 h. The reaction was warmed to RT and pH adjusted to 7 by adding solid NaHCO_3_. Then, the mixture was partitioned between H_2_O and DCM. The organic layer was dried, filtered, and evaporated under reduced pressure to obtain the title compounds **15a**–**f** as solids.
*6,7-Difluoro-2-methyl-4H-benzo[d][1,3]oxazin-4-one* (**15a**). Yellow solid, 83% yield. Spectroscopic data are in agreement with those reported [16].
*7-Chloro-2-methyl-4H-benzo[d][1,3]oxazin-4-one* (**15b**). Yellow solid, 92% yield. Spectroscopic data are in agreement with those reported [16].
*7-Bromo-2-methyl-4H-benzo[d][1,3]oxazin-4-one* (**15c**). White solid, 94% yield. Spectroscopic data are in agreement with those reported [26].
*6-Chloro-2-methyl-4H-benzo[d][1,3]oxazin-4-one* (**15d**). Yellow solid, 90% yield. ^1^H NMR (300 MHz, DMSO-*d_6_*) δ 8.02 (d, J = 2.3 Hz, 1H), 7.91 (dd, *J* = 8.6, 2.4 Hz, 1H), 7.56 (d, *J* = 8.6 Hz, 1H), 2.38 (s, 3H).
*6,7-Difluoro-2-nonyl-4H-benzo[d][1,3]oxazin-4-one* (**15e**). White solid, 52% yield. Spectroscopic data are in agreement with those reported [27].
*7-Chloro-2-nonyl-4H-benzo[d][1,3]oxazin-4-one* (**15f**). White solid, 50% yield. Spectroscopic data are in agreement with those reported [25].
*General procedure for the synthesis of compounds* **3**, **4**, and **16**–**18**. To a solution of the corresponding lactone **15a**–**f** (356 mg, 1 eq.) in EtOH (6 mL), hydrazine hydrate (150 µL, 4 eq.) was added. The mixture was heated at 80 °C for 15 h. Then, the mixture was warmed at RT and treated with a saturated solution of NaHCO_3_ and EtOAc (3 × 10 mL). The combined organics were dried, filtered, and evaporated under reduced pressure. The title compounds **3**, **4**, and **16**–**18** have been purified through silica gel column chromatography, eluent petroleum ether/EtOAc (3:1).
*3-Amino-6,7-difluoro-2-nonylquinazolin-4(3H)-one* (**3**). White solid, 26% yield. Spectroscopic data are in agreement with those reported [16].
*3-Amino-7-chloro-2-nonylquinazolin-4(3H)-one* (**4**). White solid, 66% yield. Spectroscopic data are in agreement with those reported [25].
*3-Amino-6,7-difluoro-2-methylquinazolin-4(3H)-one* (**16**). Yellow solid, 62% yield. Spectroscopic data are in agreement with those reported [28].
*3-Amino-7-chloro-2-methylquinazolin-4(3H)-one* (**17**). Yellow solid, 100% yield. ^1^H NMR (300 MHz, CDCl_3_) δ 8.15 (d, 1H *J* = 8.6 Hz), 7.63 (d, 1H, *J* = 2.0 Hz), 7.40 (dd, 1H, *J* = 8.6, 2.0 Hz), 4.88 (bs, 2H), 2.70 (s, 3H). ESI-MS *m/z*: 210 [M + H]^+^.
*3-Amino-7-bromo-2-methylquinazolin-4(3H)-one* (**18**). White solid, 68% yield. ^1^H NMR (300 MHz, Acetone-*d*_6_) δ 8.04 (s, 1H), 7.82–7.71 (m, 1H), 7.66–7.54 (m, 1H), 5.59 (s, 2H), 2.65 (s, 3H). ESI-MS *m/z*: 255 [M + H]^+^.
*General procedure for the synthesis of compounds* **7a**–**d** and **8a**,**b**. To a mixture of **3**, **16**–**18** (100 mg, 1 eq.) and the appropriate aldehyde (1 eq.) in EtOH (3 mL), AcOH (2 drops) was added. The mixture was heated at 80 °C for 20 h. The reaction mixture was then cooled to 25 °C and partitioned between H_2_O and EtOAc (3 × 10 mL). The combined organics were dried, filtered, and evaporated under reduced pressure. The title compounds were purified through silica gel column chromatography, eluent petroleum ether/EtOAc (3:1).
*(E)-6,7-Difluoro-3-((4-hydroxybenzylidene)amino)-2-nonylquinazolin-4(3H)-one* (**7a**). White solid, Mp: 134 °C, 59% yield. ^1^H NMR (300 MHz, DMSO-*d*_6_) δ 10.42 (s, 1H), 8.69 (s, 1H), 8.07–7.90 (m, 1H), 7.82–7.57 (m, 3H), 6.91 (d, *J* = 8.6 Hz, 2H), 2.86–2.61 (m, 2H), 1.74–1.40 (m, 2H), 1.43–0.94 (m, 12H), 0.80 (t, *J* = 6.8 Hz, 3H). ^13^C NMR (75 MHz, DMSO-*d*_6_) δ 170.4, 162.4, 157.2 (d, *J* = 14.9 Hz), 152.5 (m), 150.9 (m), 145.66 (d, *J* = 14.3 Hz), 144.5 (d, *J* = 45.9 Hz), 131.4, 123.4, 118.5 (d, *J* = 18.3 Hz), 116.4, 115.4, 34.3, 31.7, 29.2, 29.0, 28.9, 26.2, 22.5, 14.4. ESI-MS *m/z*: 428 [M + H]^+^. LC-MS Rt: 14.036 min, purity 100%, MS: 428.2. HRMS ESI *m/z* [M + H]^+^ calcd for C_24_H_28_F_2_N_3_O_2_ 428.2144, found 428.2126.
*(E)-6,7-Difluoro-3-((4-hydroxybenzylidene)amino)-2-methylquinazolin-4(3H)-one* (**7b**). White solid, Mp: 245 °C, 32% yield. ^1^H NMR (300 MHz, DMSO-*d*_6_) δ 10.41 (s, 1H), 8.69 (s, 1H), 8.13–7.92 (m, 1H), 7.87–7.59 (m, 3H), 6.92 (d, *J* = 8.5 Hz, 2H), 2.46 (s, 3H). ^13^C NMR (75 MHz, DMSO-*d*_6_) δ 170.3, 162.5, 154.9 (m), 147.0 (m), 144.7 (m), 131.5, 130.4, 123.4, 116.5, 115.2 (d, *J* = 17.6 Hz), 114.2, 49.0, 22.6. ESI-MS *m/z*: 316 [M + H]^+^. LC-MS Rt: 10.135 min, purity 95.5%, MS: 316.0.
*(E)-7-Chloro-3-((4-hydroxybenzylidene)amino)-2-methylquinazolin-4(3H)-one* (**7c**). White solid, Mp: 285 °C, 47% yield. ^1^H NMR (300 MHz, DMSO-*d*_6_) δ 10.40 (s, 1H), 8.70 (s, 1H), 8.11 (d, *J* = 8.6 Hz, 1H), 7.83–7.65 (m, 3H), 7.53 (dd, *J* = 8.5, 1.6 Hz, 1H), 6.92 (d, *J* = 8.5 Hz, 2H), 2.47 (s, 3H, under DMSO). ^13^C NMR (75 MHz, CDCl_3_) δ 169.1, 158.1, 155.2, 146.8, 134.9, 130.3, 129.8, 129.4, 129.0, 128.6, 127.8, 123.6, 120.2, 22.7. ESI-MS *m/z*: 314 [M + H]^+^. LC-MS Rt: 10.341 min, purity 98.7%, MS: 313.9.
*(E)-7-Bromo-3-((4-hydroxybenzylidene)amino)-2-methylquinazolin-4(3H)-one* (**7d**). White solid, Mp: 280 °C, 65% yield. ^1^H NMR (300 MHz, DMSO-*d*_6_) δ 10.39 (s, 1H), 8.70 (s, 1H), 8.03 (d, *J* = 8.5 Hz, 1H), 7.85 (d, *J* = 1.8 Hz, 1H), 7.78 (d, *J* = 8.6 Hz, 2H), 7.66 (dd, *J* = 8.5, 1.8 Hz, 1H), 6.92 (d, *J* = 8.6 Hz, 2H), 2.50 (m, 3H, under DMSO). ^13^C NMR (75 MHz, DMSO-*d_6_*) δ 171.5, 164.0, 159.2, 156.5, 148.9, 134.5, 133.0, 132.1, 129.4, 126.7, 121.3, 117.4, 115.1, 24.0 (under DMSO). ESI-MS *m/z*: 358 [M + H]^+^, 356 [M − H]^−^. LC-MS Rt: 10.494 min, purity 100%, MS: 357.90, 359.9.
*(E)-6,7-Difluoro-2-nonyl-3-4-phenylbuta-1,3-dien-1-yl)quinazolin-4(3H)-one* (**8a**). White solid, Mp: 94 °C, 49% yield. ^1^H NMR (300 MHz, CDCl_3_) δ 8.64 (d, *J* = 8.9 Hz, 1H), 7.98 (t, *J* = 9.3 Hz, 1H), 7.67–6.92 (m, 8H), 2.99–2.70 (m, 2H), 1.93–1.57 (m, 2H), 1.55–1.04 (m, 12H), 0.85 (t, *J* = 6.0 Hz, 3H). ^13^C NMR (75 MHz, CDCl_3_) δ 169.48, 157.4, δ 156.5 (d, *J* = 14.9 Hz), 152.9 (d, *J* = 11.8 Hz), 150.99 (d, *J* = 14.5 Hz), 147.66 (d, *J* = 14.3 Hz), 144.3 (m), 134.9, 131.2, 130.3, 129.1, 128.9, 128.5, 127.8, 123.6, 118.1, 115.0 (m), 34.8, 31.9, 29.4, 29.3, 26.6, 22.7, 14.1. ESI-MS *m/z*: 438 [M + H]^+^. LC-MS Rt: 16.889 min, purity 95.8%, MS: 438.2.
*(E)-7-Bromo-2-methyl-3-(-(3-phenylallylidene)amino)quinazolin-4(3H)-one* (**8b**). White solid, Mp: 200 °C, 52% yield. ^1^H NMR (300 MHz, CDCl_3_) δ 8.69 (d, *J* = 8.9 Hz, 1H), 8.10 (d, *J* = 8.5 Hz, 1H), 7.84 (d, *J* = 1.7 Hz, 1H), 7.63–7.48 (m, 3H), 7.46–7.35 (m, 3H), 7.27–7.03 (m, 2H), 2.50 (s, 3H). ^13^C NMR (75 MHz, CDCl_3_) δ 169.1, 158.1, 155.2, 146.8, 134.9, 130.3, 129.8, 129.4, 129.0, 128.6, 127.8, 123.6, 120.2, 22.7. ESI-MS *m/z*: 368 [M + H]^+^. LC-MS Rt: 11.960 min, purity 95.5%, MS: 368.9, 370.0.
*General procedure for the synthesis of compounds* **19a**,**b**. Carboxylic acid **12b** or **12d** (2.0 g, 1 eq.) was diluted in formamide (4 mL). The reaction was heated at 150 °C for 20 h. Then, ice (2.0 g) was added to the reaction to favor the precipitation of the product, which was filtered and washed with H_2_O. Then, it was dried at 50 °C for 2 h.
*6-Chloroquinazolin-4(3H)-one* (**19a**). Starting from **12d**, the title compound was obtained as a white solid, 93% yield. Spectroscopic data are in agreement with those reported [29].
*7-Chloroquinazolin-4(3H)-one* (**19b**). Starting from **12b**, the title compound was obtained as a white solid, 90% yield. ^1^H NMR (300 MHz, DMSO-*d*_6_) δ 12.39 (s, 1H), 8.25–7.92 (m, 2H), 7.71 (d, *J* = 2.0 Hz, 1H), 7.54 (dd, *J* = 8.6, 2.1 Hz, 1H).
*General procedure for the synthesis of compounds* **20a**,**b** and **21**. To a mixture of **19a**,**b** (207 mg, 1 eq.) in acetone (13 mL), the appropriate aryl halide (178 mg, 1 eq.), K_2_CO_3_ (718 mg, 5 eq.), and NaI (155 mg, 1 eq.) were added. The mixture was stirred at 50 °C for 24 h. After this time, the reaction was partitioned between brine and EtOAc and the aqueous layer was washed again with EtOAc (3 × 10 mL). The combined organics were dried, filtered, and evaporated in vacuo. The title compound was purified through silica gel column chromatography, eluent petroleum ether/EtOAc (3:1).
*6-Chloro-3-(4-nitrobenzyl)quinazolin-4(3H)-one* (**20a**). Yellow solid, 66% yield. ^1^H NMR (300 MHz, DMSO-*d*_6_) δ 8.63 (s, 1H), 8.18 (d, *J* = 8.6 Hz, 2H), 8.05 (d, *J* = 2.3 Hz, 1H), 7.86 (dd, *J* = 8.7, 2.4 Hz, 1H), 7.73 (d, *J* = 8.7 Hz, 1H), 7.59 (d, *J* = 8.6 Hz, 2H), 5.32 (s, 2H). ^13^C NMR (75 MHz, DMSO-*d*_6_) δ 159.7, 148.9, 147.4, 147.1, 144.5, 135.1, 132.0, 130.0, 129.2, 125.5, 124.2, 123.3, 49.3. ESI-MS *m/z*: 316 [M + H]^+^.
*7-Chloro-3-(4-nitrobenzyl)quinazolin-4(3H)-one* (**20b**). Yellow solid, 60% yield. ^1^H NMR (300 MHz, DMSO-*d*_6_) δ 8.65 (s, 1H), 8.14 (dd, *J* = 23.0, 8.6 Hz, 3H), 7.76 (d, *J* = 1.9 Hz, 1H), 7.57 (dd, *J* = 12.5, 5.4 Hz, 3H), 5.30 (s, 2H). ^13^C NMR (75 MHz, DMSO-*d*_6_) δ 160.0, 149.8, 149.4, 147.3, 144.6, 139.6, 129.1, 128.6, 128.0, 126.9, 124.1, 120.8, 49.2. ESI-MS *m/z*: 316 [M + H]^+^.
*3-(4-Bromobenzyl)-7-chloroquinazolin-4(3H)-one* (**21**). Yellow solid, 67% yield. ^1^H NMR (300 MHz, CDCl_3_) δ 8.19 (t, *J* = 15.5 Hz, 1H), 8.08 (s, 1H), 7.69 (s, 1H), 7.47 (d, *J* = 7.4 Hz, 3H), 7.22 (d, *J* = 7.7 Hz, 2H), 5.11 (s, 2H). ^13^C NMR (75 MHz, CDCl_3_) δ 160.0, 148.9, 146.9, 134.5, 131.8, 129.7, 128.2, 127.0, 122.5, 120.1, 49.2. ESI-MS *m/z*: 350 [M + H]^+^.
*General procedure for the synthesis of compounds* **22a**,**b**. To a mixture of **20a**,**b** (182 mg, 1 eq.) in EtOH (6 mL), heated at 55 °C, saturated NH_4_Cl (6 mL) was added. After that, iron powder (330 mg, 10 eq.) was added and the mixture was heated at 70 °C for 2 h. Solvent was removed, the mixture was diluted in EtOAc and filtered on Celite^®^, then pH was adjusted to 8 by adding saturated NaHCO_3_. The mixture was then partitioned between H_2_O and EtOAc. The organic layer was dried, filtered, and evaporated to afford the final product.
*3-(4-Aminobenzyl)-6-chloroquinazolin-4(3H)-one* (**22a**). White solid, Mp: 194 °C, 54% yield. ^1^H NMR (300 MHz, DMSO-*d*_6_) δ 8.53 (s, 1H), 8.07 (d, *J* = 2.4 Hz, 1H), 7.82 (dd, *J* = 8.7, 2.5 Hz, 1H), 7.68 (d, *J* = 8.7 Hz, 1H), 7.07 (d, *J* = 8.3 Hz, 2H), 6.48 (d, *J* = 8.3 Hz, 2H), 5.09 (s, 2H), 4.97 (s, 2H). 13C NMR (75 MHz, DMSO-*d*_6_) δ 159.5, 148.9, 148.8, 147.0, 131.8, 129.9, 129.6, 125.5, 123.7, 123.3, 114.1, 49.2. ESI-MS *m/z*: 286 [M + H]^+^. LC-MS Rt: 8.251 min, purity 99.4%, MS: 286.0.
*3-(4-Aminobenzyl)-7-chloroquinazolin-4(3H)-one* (**22b**). Yellow solid, Mp: 168 °C, 95% yield. ^1^H NMR (300 MHz, DMSO-*d*_6_) δ 8.55 (s, 1H), 8.12 (d, *J* = 8.6 Hz, 1H), 7.73 (d, *J* = 1.9 Hz, 1H), 7.53 (dt, *J* = 30.1, 15.1 Hz, 1H), 7.03 (t, *J* = 19.0 Hz, 2H), 6.48 (d, *J* = 8.4 Hz, 2H), 5.11 (s, 2H), 4.96 (s, 2H). ^13^C NMR (75 MHz, DMSO-*d*_6_) δ 159.8, 149.8, 149.2, 148.9, 148.7, 139.3, 129.7, 128.5, 127.9, 126.4, 123.4, 114.1, 48.9. ESI-MS *m/z*: 286 [M + H]^+^. LC-MS Rt: 8.233 min, purity 97.7%, MS: 286.0.
*Synthesis of 7-Chloro-3-((4′-(trifluoromethyl)-[1,1′-biphenyl]-4-yl)methyl)quinazolin-4(3H)-one (**9**).* To a solution of **21** (100 mg, 1 eq.) in anhydrous EtOH (2.5 mL) and anhydrous 1,2-diethoxyethane (2.5 mL), Pd^0^ (1.3 mg, 0.04 eq.) was added. The mixture was refluxed and after 30 min, Na_2_CO_3_ (189 mg, 6 eq.) in H_2_O (1 mL) and (4-(trifluoromethyl)phenyl)boronic acid (80 mg, 1.4 eq.) in EtOH (0.5 mL) were added. The mixture was refluxed for 12 h. After this time, H_2_O was added (1 mL) and the mixture was extracted with EtOAc (10 mL). The aqueous layer was extracted twice with EtOAc (2 × 5 mL). The combined organic layers were dried, filtered, and evaporated under reduced pressure. The title compound 9 was purified through silica gel column chromatography, eluent petroleum ether/EtOAc (3:1). Yellow solid, Mp: 167 °C, 63% yield. ^1^H NMR (300 MHz, CDCl_3_) δ 8.30–7.92 (m, 2H), 7.90–7.06 (m, 10H), 5.17 (s, 2H). ^13^C NMR (75 MHz, CDCl_3_) δ 160.4, 148.9, 147.4, 147.3 (dd, *J* = 14.3, 3.6 Hz), 143.7, 140.6, 138.9, 135.4, 132.1, 129.6, 128.6, 128.3 (d, *J* = 2.0 Hz), 128.1 (d, *J* = 2.9 Hz), 127.9, 127.3, 127.1 (d, *J* = 2.7 Hz), 127.0, 125.9, 125.8 (d, *J* = 3.8 Hz), 125.7, 120.6, 49.5. ESI-MS *m/z*: 437 [M + Na]^+^. LC-MS Rt: 12.716 min, purity 95.0%, MS: 415.1.
*Synthesis of 3-(4-(1H-pyrrol-1-yl)benzyl)-7-chloroquinazolin-4(3H)-one (***10***).* According to published procedures [30,31], a mixture of 4-chloropyridine hydrochloride (79.5 mg, 5 eq.) in 1,4-dioxane was heated at 150 °C for 30 min. Then, **22b** (125 mg, 1 eq.) and 2,5-dimethoxy tetrahydrofuran (57 µL, 1 eq.) were added and the mixture was heated at 150 °C for 2 h. The reaction mixture was then filtered on Celite^®^ and the product was purified through silica gel column chromatography, eluent petroleum ether/EtOAc (3:1). White solid, Mp: 195 °C, 92% yield. ^1^H NMR (300 MHz, CDCl_3_) δ 8.24 (d, *J* = 8.6 Hz, 1H), 8.14 (s, 1H), 7.70 (d, *J* = 1.7 Hz, 1H), 7.56–7.30 (m, 5H), 7.05 (d, *J* = 1.9 Hz, 2H), 6.33 (d, *J* = 1.9 Hz, 2H), 5.18 (s, 2H). ^13^C NMR (75 MHz, CDCl_3_) δ 160.4, 148.9, 147.3, 147.2, 140.7, 140.6, 132.6, 129.4, 128.3, 128.1, 127.1, 120.8, 120.6, 119.1, 110.7, 49.3. ESI-MS *m/z*: 336 [M + H]^+^. LC-MS Rt: 11.546 min, purity 97.5%, MS: 336.0.
*General procedure for the synthesis of compounds* **11a**–**f**. To a well-stirred solution of **22a**,**b** (180 mg, 1 eq.) in MeOH (5 mL), the appropriate aldehyde (1 eq.) and AcOH (5 drops) were added. The mixture was heated at 50 °C for 12 h. After this time, the reaction was cooled to 25 °C and NaBH_3_CN (60 mg, 1.5 eq.) was added. The mixture was stirred for an additional 2 h. Then, pH was adjusted to 8 by adding a saturated NaHCO_3_ aqueous solution. The crude was partitioned between H_2_O and EtOAc; the aqueous layer was extracted with EtOAc (3 × 10 mL). The title compound was purified through silica gel column chromatography, eluent petroleum ether/EtOAc (3:1).
*6-Chloro-3-(4-((furan-2-ylmethyl)amino)benzyl)quinazolin-4(3H)-one* (**11a**). Yellow solid, Mp: 161 °C, 73% yield. ^1^H NMR (300 MHz, CDCl_3_) δ 8.26 (d, *J* = 1.8 Hz, 1H), 8.06 (s, 1H), 7.63 (q, *J* = 8.7 Hz, 2H), 7.33 (s, 1H), 7.19 (d, *J* = 8.4 Hz, 2H), 6.62 (d, *J* = 8.4 Hz, 2H), 6.30 (s, 1H), 6.20 (d, *J* = 2.8 Hz, 1H), 5.04 (s, 2H), 4.37–4.08 (m, 3H). ^13^C NMR (75 MHz, CDCl_3_) δ 160.0, 152.2, 147.7, 146.5, 142.0, 134.5, 133.0, 129.7, 129.1, 126.2, 124.3, 123.2, 113.2, 110.4, 110.4, 107.1, 49.5, 41.1. ESI-MS *m/z*: 366 [M + H]^+^, 388 [M + Na]^+^. LC-MS Rt: 11.122 min, purity 100%, MS: 365.9. HRMS ESI *m/z* [M + Na]^+^ calcd for C_20_H_16_ClN_3_O_2_Na 388.0823, found 388.0809.
*7-Chloro-3-(4-((furan-2-ylmethyl)amino)benzyl)quinazolin-4(3H)-one* (**11b**). White solid, Mp: 126 °C, 70% yield. ^1^H NMR (300 MHz, Acetone-*d*_6_) δ 8.41 (s, 1H), 8.19 (d, *J* = 8.6 Hz, 1H), 7.64 (d, *J* = 1.9 Hz, 1H), 7.50 (dd, *J* = 8.6, 2.0 Hz, 1H), 7.42 (s, 1H), 7.25 (d, *J* = 8.5 Hz, 3H), 6.69 (d, *J* = 8.5 Hz, 3H), 6.27 (dd, *J* = 17.7, 2.4 Hz, 2H), 5.47 (t, *J* = 5.3 Hz, 1H), 5.10 (s, 3H), 4.30 (d, *J* = 5.9 Hz, 3H). ^13^C NMR (75 MHz, Acetone-*d*_6_) δ 159.7, 153.3, 149.4, 148.7, 148.3, 141.7, 139.3, 129.4, 128.2, 127.1, 126.6, 124.5, 121.0, 112.6, 110.2, 106.6, 48.8, 40.3. ESI-MS *m/z*: 366 [M + H]^+^. LC-MS Rt: 11.154 min, purity 99.1%, MS: 366.1. HRMS ESI *m/z* [M + H]^+^ calcd for C_20_H_17_ClN_3_O_2_ 366.1004, found 366.0993.
*6-Chloro-3-(4-(((2,3-dihydrobenzofuran-5-yl)methyl)amino)benzyl)quinazolin-4(3H)-one* (**11c**). White solid, Mp: 153 °C, 75% yield. ^1^H NMR (300 MHz, DMSO-*d*_6_) δ 8.53 (s, 1H), 8.05 (s, 1H), 7.82 (dd, *J* = 8.7, 2.5 Hz, 1H), 7.67 (d, *J* = 8.7 Hz, 1H), 7.23–7.05 (m, 3H), 7.00 (d, *J* = 7.9 Hz, 1H), 6.64 (d, *J* = 8.1 Hz, 1H), 6.49 (d, *J* = 8.5 Hz, 2H), 6.23 (t, *J* = 5.9 Hz, 1H), 4.97 (s, 2H), 4.44 (t, *J* = 8.7 Hz, 2H), 4.09 (d, *J* = 5.8 Hz, 2H), 3.08 (t, *J* = 8.6 Hz, 2H). ^13^C NMR (75 MHz, DMSO-*d*_6_) δ 159.6, 159.1, 148.8, 147.1, 134.9, 132.2, 131.9, 129.9, 129.6, 127.6, 127.1, 125.5, 124.4, 123.7, 123.3, 112.5, 108.9, 71.2, 49.2, 46.3, 29.6. ESI-MS *m/z*: 418 [M + H]^+^. LC-MS Rt: 11.383 min, purity 95.1%, MS: 418.0. HRMS ESI *m/z* [M + H]^+^ calcd for C_24_H_21_ClN_3_O_2_ 418.1317, found 418.1302.
*7-Chloro-3-(4-(((2,3-dihydrobenzofuran-5-yl)methyl)amino)benzyl)quinazolin-4(3H)-one* (**11d**). White solid, 181 °C, 72% yield. ^1^H NMR (300 MHz, DMSO-*d*_6_) δ 8.54 (s, 1H), 8.11 (d, *J* = 8.6 Hz, 1H), 7.72 (d, *J* = 1.9 Hz, 1H), 7.55 (dd, *J* = 8.6, 2.0 Hz, 1H), 7.24–6.90 (m, 4H), 6.64 (d, *J* = 8.1 Hz, 1H), 6.50 (d, *J* = 8.5 Hz, 2H), 6.20 (t, *J* = 5.9 Hz, 1H), 4.96 (s, 2H), 4.44 (t, *J* = 8.7 Hz, 2H), 4.10 (d, *J* = 5.9 Hz, 2H), 3.09 (t, *J* = 8.7 Hz, 2H). ^13^C NMR (75 MHz, DMSO-*d*_6_) δ 159.9, 159.0, 149.8, 148.7, 132.2, 129.6, 128.9, 127.8, 127.2, 126.8, 124.7, 123.6, 120.7, 112.7, 108.8, 71.2, 49.3, 46.7, 29.5. ESI-MS *m/z*: 418 [M + H]^+^. LC-MS Rt: 11.418 min, purity 99.1%, MS: 418.0. HRMS ESI *m/z* [M + H]^+^ calcd forC_24_H_21_ClN_3_O_2_ 418.1317, found 418.1304.
*6-Chloro-3-(4-((ferrocene-2-ylmethyl)amino)benzyl)quinazolin-4(3H)-one* (**11e**). Red solid, Mp: 112 °C, 70% yield. ^1^H NMR (300 MHz, CDCl_3_) δ 8.29 (d, *J* = 2.6 Hz, 1H), 8.09 (d, *J* = 6.1 Hz, 1H), 7.71–7.55 (m, 3H), 7.18 (dd, *J* = 17.4, 7.8 Hz, 2H), 6.61 (d, *J* = 8.5 Hz, 2H), 5.07 (s, 3H), 4.22 (s, 3H), 4.17 (s, 6H), 4.14 (s, 2H), 3.93 (s, 2H). ^13^C NMR (75 MHz, CDCl_3_) δ 160.1, 148.3, 146.5, 135.3, 134.6, 133.0, 129.8, 129.1, 126.2, 123.8, 123.3, 113.0, 86.0, 70.3, 69.5, 69.1, 68.3, 49.6, 43.2. ESI-MS *m/z*: 484 [M + H]^+^. LC-MS Rt: 12.287 min, purity 97.9%, MS: 483.0.
*7-Chloro-3-(4-((ferrocene-2-ylmethyl)amino)benzyl)quinazolin-4(3H)-one* (**11f**). Red solid, Mp: 155 °C, 75% yield. ^1^H NMR (300 MHz, CDCl_3_) δ 8.22 (d, *J* = 8.5 Hz, 1H), 8.07 (s, 1H), 7.66 (s, 1H), 7.40 (d, *J* = 8.5 Hz, 1H), 7.20 (d, *J* = 8.2 Hz, 2H), 6.60 (d, *J* = 8.2 Hz, 2H), 5.03 (s, 2H), 4.31–4.01 (m, 9H), 3.92 (s, 2H). ^13^C NMR (75 MHz, CDCl_3_) δ 160.5, 149.0, 148.3, 147.6, 140.2, 129.8, 128.3, 127.7, 126.9, 123.8, 120.7, 112.9, 86.0, 68.5, 68.1, 67.9, 49.5, 43.2. ESI-MS *m/z*: 484 [M + H]^+^. LC-MS Rt: 12.302 min, purity 99.4%, MS: 483.1.

### 3.2. Biological Methods

#### 3.2.1. Bacterial Strains

Two PA strains were tested in the present study: RP73, a multi-drug resistant strain isolated 17 years after the onset of infection in a CF patient from the Hannover cohort [32]; and BJ3525, a multi-drug resistant strain causing first infection. The strains were identified using MALDI-TOF mass spectrometry and then stored at −80 °C until cultured twice on Tryptone Soya Agar (TSA; Oxoid, Milan, Italy) to restore their original phenotypes.

#### 3.2.2. “CF-like” Experimental Conditions

To simulate the physical-chemical properties observed in the CF airways, MIC and biofilm assays were carried out under “CF-like” conditions [33,34], namely using an artificial sputum medium (ASM) under acid conditions (pH 6.8) and at 5% CO_2_ atmosphere. ASM had a composition closely resembling CF sputum [35], with some modifications. All ingredients, except Casamino acids, were from Merk Life Science S.r.l. (Milan, Italy): 5 g mucin from pig stomach type II, 4 g DNA from herring sperm, 5.9 mg diethylene triamine pentaacetic acid, 5 g NaCl, 2.2 g KCl, 1 g Trizma base, 5 mL egg yolk emulsion, and 5 g Casamino acids (Life Technologies Italia, Monza, Italy) per 1 L water.

#### 3.2.3. Drug Susceptibility Assays of Planktonic Cells: MIC and MBC Measurements

Several colonies from an overnight growth at 37 °C onto TSA (Oxoid) were resuspended in sterile NaCl 0.9% (Fresenius Kabi Italia, Verona, Italy), adjusted to a final concentration of 1–2 × 10^8^ CFU/mL, and finally diluted 1:10 (*vol*/*vol*) in sterile saline. Five microliters of this standardized inoculum were added to each well of a microtiter plate containing 100 µL ASM with the compound at the desired concentration. Each compound was tested at concentrations ranging from 0.1 to 50 µM, whereas tobramycin (Merck, Milan, Italy) was tested in the 512-1 µg/mL range. After 24 h incubation at 37 °C, the MIC value was read as the lowest concentration inhibiting visible bacterial growth. The MBC value was evaluated by plating onto Mueller–Hinton Agar (MHA; Oxoid) 10 μL of broth culture from wells showing no visible growth at MIC determination. Following incubation at 37 °C for 24 h, the MBC value was defined as the minimum antibiotic concentration needed to eradicate 99.9% of the starting inoculum. Differences between MIC values and those between MBC values were considered significant for discrepancies ≥2 log_2_ concentration steps [36].

#### 3.2.4. Biofilm Formation Assay

For each PA strain, several colonies grown overnight onto TSA were resuspended in Trypticase Soy Broth (TSB; Oxoid) and incubated at 37 °C under agitation (130 rpm). After 16 h of incubation, the broth culture was adjusted with sterile TSB to an optical density measured at 550 nm (OD_550_) of 0.8, corresponding to 1–4 × 10^9^ CFU/mL, and finally diluted 1:100 (*vol*/*vol*) in ASM. In each well of a 96-well flat-bottom polystyrene tissue culture microtiter plate (Becton, Dickinson & Co., Milan, Italy), 200 μL of the standardized inoculum was aliquoted, and then the test agents were added to reach a final concentration of 50 µM. Negative controls were prepared similarly using ASM and 2.5% DMSO without test compounds. After 24 h of incubation at 37 °C under static and CF-like conditions, samples were washed twice with phosphate-buffered saline (PBS; Merck, Italy) pH 7.2 to remove non-adherent cells. Samples were fixed at 60 °C for one hour, and then biofilm biomass was stained for 5 min with 200 µL of Hucker-modified crystal violet [37] (Merck, Italy) and air-dried (37 °C, 30 min). Finally, crystal violet was extracted by exposure for 15 min to 200 μL of 33% glacial acetic acid (Merck, Italy). Biofilm biomass was measured as OD_492_ (Infinite^®^ M PLEX; Tecan, Milan, Italy). The cutoff value (ODc) for biofilm formation was defined as the mean OD of negative controls + 3× standard deviation.

#### 3.2.5. Pyocyanin and Pyoverdine Formation Assays

Several colonies grown on TSA were resuspended in 5 mL Luria Bertani broth (LB; ThermoFisher Scientific Italia, Monza, Italy) to achieve an OD_600_ of 0.5 (corresponding to 1–3 × 10^8^ CFU/mL). This standardized inoculum was diluted 1:10 (*vol*/*vol*) with fresh LB broth and incubated with test compounds, each tested at 50 µM, in static conditions at 37 °C for 48 h. Control samples were prepared in LB and 0.25% DMSO without test compounds. Following incubation, the OD_600_ of the exposed and control cultures was measured, and the bacterial load was checked by CFU count, resulting in no significant difference. Next, cultures were centrifuged (10,000 rpm, 15 min, at RT), and the supernatants were 0.2 µm filtered before quantifying virulence factors. Pyocyanin pigment was extracted by exposure to chloroform (Merck, Italy) and 0.2 N hydrochloric acid (Merck, Italy) and quantified at OD_520_ [38]. Pyoverdine was quantified by measuring the OD_400_ of supernatants. The value obtained for each virulence factor was multiplied by the ratio OD_600_ of the control/OD_600_ of the sample to normalize for slight differences in the culture densities between the controls and exposed samples after 48 h of incubation.

#### 3.2.6. Cytotoxicity Evaluation

The cytotoxic potential of each test compound was evaluated using IB3-1 bronchial epithelial cells (ATCC#CRL-2777) isolated from a pediatric CF patient who harbored the ΔF508/W1282X mutations within the CFTR gene. Cells were grown at 37 °C in LHC-8 medium (Thermo Fisher Scientific Italia, Rodano, Italy) supplemented with 5% fetal bovine serum (Gibco, Monza, Italy) under a 5% CO_2_ atmosphere, and once the confluence was reached, exposed to each compound at 50 µM. After 24 h exposure at 37 °C, the test compound was removed by washing with a sterile medium, and the cell viability was measured by an MTS tetrazolium-based colorimetric assay (CellTiter 96^®^ AQueous One Solution Cell Proliferation Assay; Promega, Milan, Italy). Briefly, 20 μL of a mixture of MTS [3-(4,5-dimethylthiazol-2-yl)-5-(3-carboxymethoxyphenyl)-2-(4-sulfophenyl)-2H-tetrazolium] and the electron coupling reagent PES (phenazine ethosulfate) were added to each well containing exposed cells. Untreated IB3-1 cells were used as the control. After 4 h of incubation at 37 °C, the OD_492_ was measured using an ELISA plate reader (Infinite^®^ M PLEX; Tecan) [39].

### 3.3. Statistical Analysis

Each experiment was carried out at least in triplicate and repeated on two different occasions (n ≥ 6). Statistical analysis was performed using GraphPad software (ver. 8.0; GraphPad Inc., San Diego, CA, USA). Data distribution was assessed using the D’Agostino and Pearson normality test. The differences were evaluated using ordinary one-way ANOVA followed by Holm–Sidak’s (biofilm formation, pyoverdine production) or Tukey’s (pyocyanin production) multiple comparisons test, with a simple pooled variance. The significance level was set at *p* < 0.05.

### 3.4. Molecular Modeling

#### 3.4.1. Protein and Ligand Preparation

The selected derivatives were drawn in Maestro using the drawing tools of the software and then were prepared employing MacroModel and LigPrep as previously reported [40,41,42]. The selected molecules were minimized using a MacroModel with the OPLS3 force field [43]. A GB/SA solvation model for simulating the solvent effect was employed with “no cutoff” for non-bonded interactions. The PRCG method (5000 maximum iterations and 0.001 gradient convergence threshold) was used. Lastly, the LigPrep program was used for optimizing the molecules, generating possible ionization states at pH 7.4 ± 0.2. PsqR obtained from PA (PDB ID 6B8A) [24] was downloaded from the Protein Data Bank (PDB) and prepared using Protein Preparation Wizard implemented in Maestro Suite. The materials used for the crystallization process were removed.

#### 3.4.2. Molecular Docking

To conduct molecular docking studies, we used the software Glide (Grid-Based Ligand Docking with Energetics) (Glide version 8.8, Schrödinger, LLC, New York, NY, USA, 2020) employing compounds and the protein prepared as mentioned above, applying Glide standard precision (SP) as a scoring function [44]. Energy grids were prepared using the default value of the protein atom scaling factor (1.0 Å) within a cubic box centered on the crystallized ligand [24]. Subsequently, the selected derivatives were docked into the selected binding site using default parameters. The number of poses entered for post-docking minimization was set to 50. Glide SP scores were evaluated. The interactions of drugs with protein were assessed using the ligand interaction diagram available in the Maestro suite. The docking protocol was validated considering the re-docking procedure using the crystallized ligand. The presented docking protocol correctly accommodated the reference ligand with a small RMSD value of the docking pose with respect to the crystallized one (RMSD = 0.176 Å).

#### 3.4.3. Molecular Dynamics Simulation

MD simulations were carried out by the Desmond 6.4 academic version, provided by D. E. Shaw Research (“DESRES”), using Maestro 12.6 as the graphical interface (Desmond Molecular Dynamics System, D. E. Shaw Research, New York, NY, USA, 2020. Maestro-Desmond Interoperability Tools, Schrödinger, New York, NY, USA, 2020). MD was performed using the Compute Unified Device Architecture (CUDA) API on two NVIDIA GPUs. The four complexes derived from docking studies were imported in Maestro, and using a Desmond system builder, were solvated into an orthorhombic box filled with water, simulated by the TIP3P model [45,46]. The OPLS force field was utilized for MD calculations. Na^+^ and Cl^−^ ions were added to provide a final salt concentration of 0.15 M to simulate the physiological concentration of monovalent ions. Constant temperature (300 K) and pressure (1.01325 bar) were employed with NPT (constant number of particles, pressure, and temperature) as an ensemble class. An RESPA integrator was applied to integrate the equations of motion, with an inner time step of 2.0 fs for bonded and non-bonded interactions within the short-range cutoff. Nose–Hoover thermostats [47] were employed to maintain the constant simulation temperature, and the Martyna–Tobias–Klein method was utilized to control the pressure. Long-range electrostatic interactions were estimated by the particle mesh Ewald (PME) technique [48]. The cutoff for van der Waals and short-range electrostatic interactions was set at 9.0 Å. The equilibration of the system was performed with the default protocol provided in Desmond, which consists of a series of restrained minimizations and MD simulations applied to relax the system slowly. Consequently, one individual trajectory for each complex of 200 ns was calculated. MD simulation experiments were repeated twice for each complex to improve the presented results. The trajectory files were analyzed by simulation event analysis and simulation interaction diagram tools implemented in the Maestro graphical interface. The same applications were used to generate all plots concerning MD simulation experiments presented in this work. Accordingly, the RMSD was evaluated using the following equation:

RMSDx=1N∑i=1Nr′itx−⁡ri(tref)2

where the RMSD_x_ is referred to as the calculation for a frame x, N is the number of atoms in the atom selection; t_ref_ is the reference time (typically the first frame is used as the reference and it is regarded as time t = 0); and r’ is the position of the selected atoms in frame x, after superimposing on the reference frame, where frame x is recorded at time t_x_. The procedure was repeated for every frame in the simulation trajectory. Regarding the RMSF, the following equation was used for the calculation:

RMSFi=1T∑t=1T<r′it−⁡ri(tref)2>

where RMSF_i_ is referred to as generic residue I, T is the trajectory time over which the RMSF is calculated, t_ref_ is the reference time, r_i_ is the position of residue i; r’ is the position of atoms in residue i after superposition on the reference, and the angle brackets indicate that the average of the square distance is taken over the selection of atoms in the residue.

## 4. Conclusions

To obtain new compounds with the potential to reduce biofilm formation and production of virulence factors such as pyocyanin and pyoverdine in two clinical isolates of PA strains in CF-like conditions, the quinazolin-4(3*H*)-one core has been selected as the privileged scaffold. Differently decorated compounds were synthesized and tested, namely the 2-methyl- or 2-nonyl-quinazolin-4(3*H*)-one based hydrazones **7a**–**d** and **8a**,**b**, and *N*-benzyl substituted quinazolin-4(3*H*)-one compounds **9**, **10**, and **11a**–**f**. Most of the synthesized compounds were able to reduce biofilm formation and virulence factors (pyocyanin and pyoverdine) in the two PA strains BJ3525 (acute CF-like condition) and RP73 (chronic CF-like condition), although with different degrees of efficacy at the sub-inhibitory concentration of 50 µM. Furthermore, the quinazolinone derivatives did not reduce the cell viability of IB3-1 bronchial CF cells and, when docked in the PqsR active site, demonstrated to be potential candidates for developing new Pqs system modulators.

## Data Availability

Not applicable.

## Supplementary Materials

The following supporting information can be downloaded at: https://www.mdpi.com/article/10.3390/molecules28186535/s1. (Chemical Compounds Characterization: 1H, 13C NMR and HPLC/MS spectra of compounds).
