# Supplementary material for: Development of Quinazolinone Derivatives as Modulators of Virulence Factors of Pseudomonas aeruginosa Cystic Fibrosis Strains
Source: Molecules. 2023 Sep 9;28(18):6535. doi: 10.3390/molecules28186535 (PMC10536951; doi:10.3390/molecules28186535)
Supplement: Supplementary file 1 [file molecules-28-06535-s001.zip › molecules_cmpd-check-list molecules-2582478.pdf]

molecules

MDPI

Chemical Compounds Characterization Checklist

Manuscript ID: molecules-2582478

Submitting Author: Sandra Gemma

Compound			Characterization											
Compound Nr.	New	Known (Ref. in main text)	Physical State (purity %)	1H NMR	13C NMR	2D NMR	HRMS	IR	XRD	GC/ HPLC				Data in SI
7a	☑		solid (100%	☑	☑	☐	☑	☐	☐	☑	☐	☐	☐	☑
7b	☑		solid (95.5%	☑	☑	☐	☐	☐	☐	☑	☐	☐	☐	☑
7c	☑		solid (98.7%	☑	☑	☐	☐	☐	☐	☑	☐	☐	☐	☑
7d	☑		solid (100%	☑	☑	☐	☐	☐	☐	☑	☐	☐	☐	☑
8a	☑		solid (95.8%	☑	☑	☐	☐	☐	☐	☑	☐	☐	☐	☑
8b	☑		solid (95.5%	☑	☑	☐	☐	☐	☐	☑	☐	☐	☐	☑
9	☑		solid (95.0%	☑	☑	☐	☐	☐	☐	☑	☐	☐	☐	☑
10	☑		solid (95.5%	☑	☑	☐	☐	☐	☐	☑	☐	☐	☐	☑
11a	☑		solid (100%	☑	☑	☐	☑	☐	☐	☑	☐	☐	☐	☑
11b	☑		solid (99.1%	☑	☑	☐	☑	☐	☐	☑	☐	☐	☐	☑
11c	☑		solid (95.1%	☑	☑	☐	☑	☐	☐	☑	☐	☐	☐	☑
11d	☑		solid (99.1%	☑	☑	☐	☑	☐	☐	☑	☐	☐	☐	☑

Note: insert the relevant information and select only the techniques used in this study. In the empty columns you can insert any additional methods.

Compound			Characterization											
Compound Nr.	New	Known (Ref. in main text)	Physical State (purity %)	1H NMR	13C NMR	2D NMR	HRMS	IR	XRD	GC/ HPLC				Data in SI
11e	☑		solid (97.9%	☑	☑	☐	☐	☐	☐	☑	☐	☐	☐	☑
11f	☑		solid (99.4%	☑	☑	☐	☐	☐	☐	☑	☐	☐	☐	☑
	☐			☐	☐	☐	☐	☐	☐	☐	☐	☐	☐	☐
	☐			☐	☐	☐	☐	☐	☐	☐	☐	☐	☐	☐
	☐			☐	☐	☐	☐	☐	☐	☐	☐	☐	☐	☐
	☐			☐	☐	☐	☐	☐	☐	☐	☐	☐	☐	☐
	☐			☐	☐	☐	☐	☐	☐	☐	☐	☐	☐	☐
	☐			☐	☐	☐	☐	☐	☐	☐	☐	☐	☐	☐
	☐			☐	☐	☐	☐	☐	☐	☐	☐	☐	☐	☐
	☐			☐	☐	☐	☐	☐	☐	☐	☐	☐	☐	☐
	☐			☐	☐	☐	☐	☐	☐	☐	☐	☐	☐	☐
	☐			☐	☐	☐	☐	☐	☐	☐	☐	☐	☐	☐
	☐			☐	☐	☐	☐	☐	☐	☐	☐	☐	☐	☐
	☐			☐	☐	☐	☐	☐	☐	☐	☐	☐	☐	☐
	☐			☐	☐	☐	☐	☐	☐	☐	☐	☐	☐	☐
	☐			☐	☐	☐	☐	☐	☐	☐	☐	☐	☐	☐

Compound			Characterization											
Compound Nr.	New	Known (Ref. in main text)	Physical State (purity %)	1H NMR	13C NMR	2D NMR	HRMS	IR	XRD	GC/ HPLC				Data in SI
	☐			☐	☐	☐	☐	☐	☐	☐	☐	☐	☐	☐
	☐			☐	☐	☐	☐	☐	☐	☐	☐	☐	☐	☐
	☐			☐	☐	☐	☐	☐	☐	☐	☐	☐	☐	☐
	☐			☐	☐	☐	☐	☐	☐	☐	☐	☐	☐	☐
	☐			☐	☐	☐	☐	☐	☐	☐	☐	☐	☐	☐
	☐			☐	☐	☐	☐	☐	☐	☐	☐	☐	☐	☐
	☐			☐	☐	☐	☐	☐	☐	☐	☐	☐	☐	☐
	☐			☐	☐	☐	☐	☐	☐	☐	☐	☐	☐	☐
	☐			☐	☐	☐	☐	☐	☐	☐	☐	☐	☐	☐
	☐			☐	☐	☐	☐	☐	☐	☐	☐	☐	☐	☐
	☐			☐	☐	☐	☐	☐	☐	☐	☐	☐	☐	☐
	☐			☐	☐	☐	☐	☐	☐	☐	☐	☐	☐	☐
	☐			☐	☐	☐	☐	☐	☐	☐	☐	☐	☐	☐
	☐			☐	☐	☐	☐	☐	☐	☐	☐	☐	☐	☐
	☐			☐	☐	☐	☐	☐	☐	☐	☐	☐	☐	☐
	☐			☐	☐	☐	☐	☐	☐	☐	☐	☐	☐	☐

molecules

MDPI

Compound			Characterization											
Compound Nr.	New	Known (Ref. in main text)	Physical State (purity %)	1H NMR	13C NMR	2D NMR	HRMS	IR	XRD	GC/ HPLC				Data in SI
	☐			☐	☐	☐	☐	☐	☐	☐	☐	☐	☐	☐
	☐			☐	☐	☐	☐	☐	☐	☐	☐	☐	☐	☐
	☐			☐	☐	☐	☐	☐	☐	☐	☐	☐	☐	☐
	☐			☐	☐	☐	☐	☐	☐	☐	☐	☐	☐	☐
	☐			☐	☐	☐	☐	☐	☐	☐	☐	☐	☐	☐
	☐			☐	☐	☐	☐	☐	☐	☐	☐	☐	☐	☐
	☐			☐	☐	☐	☐	☐	☐	☐	☐	☐	☐	☐
	☐			☐	☐	☐	☐	☐	☐	☐	☐	☐	☐	☐
	☐			☐	☐	☐	☐	☐	☐	☐	☐	☐	☐	☐
	☐			☐	☐	☐	☐	☐	☐	☐	☐	☐	☐	☐
	☐			☐	☐	☐	☐	☐	☐	☐	☐	☐	☐	☐
	☐			☐	☐	☐	☐	☐	☐	☐	☐	☐	☐	☐
	☐			☐	☐	☐	☐	☐	☐	☐	☐	☐	☐	☐
	☐			☐	☐	☐	☐	☐	☐	☐	☐	☐	☐	☐
	☐			☐	☐	☐	☐	☐	☐	☐	☐	☐	☐	☐
	☐			☐	☐	☐	☐	☐	☐	☐	☐	☐	☐	☐

molecules

MDPI

Compound			Characterization											
Compound Nr.	New	Known (Ref. in main text)	Physical State (purity %)	1H NMR	13C NMR	2D NMR	HRMS	IR	XRD	GC/ HPLC				Data in SI
	☐			☐	☐	☐	☐	☐	☐	☐	☐	☐	☐	☐
	☐			☐	☐	☐	☐	☐	☐	☐	☐	☐	☐	☐
	☐			☐	☐	☐	☐	☐	☐	☐	☐	☐	☐	☐
	☐			☐	☐	☐	☐	☐	☐	☐	☐	☐	☐	☐
	☐			☐	☐	☐	☐	☐	☐	☐	☐	☐	☐	☐
	☐			☐	☐	☐	☐	☐	☐	☐	☐	☐	☐	☐
	☐			☐	☐	☐	☐	☐	☐	☐	☐	☐	☐	☐
	☐			☐	☐	☐	☐	☐	☐	☐	☐	☐	☐	☐
	☐			☐	☐	☐	☐	☐	☐	☐	☐	☐	☐	☐
	☐			☐	☐	☐	☐	☐	☐	☐	☐	☐	☐	☐
	☐			☐	☐	☐	☐	☐	☐	☐	☐	☐	☐	☐
	☐			☐	☐	☐	☐	☐	☐	☐	☐	☐	☐	☐
	☐			☐	☐	☐	☐	☐	☐	☐	☐	☐	☐	☐
	☐			☐	☐	☐	☐	☐	☐	☐	☐	☐	☐	☐
	☐			☐	☐	☐	☐	☐	☐	☐	☐	☐	☐	☐
	☐			☐	☐	☐	☐	☐	☐	☐	☐	☐	☐	☐
