# Supplementary material for: Development of Quinazolinone Derivatives as Modulators of Virulence Factors of Pseudomonas aeruginosa Cystic Fibrosis Strains
Source: Molecules. 2023 Sep 9;28(18):6535. doi: 10.3390/molecules28186535 (PMC10536951; doi:10.3390/molecules28186535)
Supplement: Supplementary file 1 [file molecules-28-06535-s001.zip › Supporting Information_V1_2023_08_30.pdf]

Supplementary Information for:

Development of quinazolinone derivatives as modulators of virulence factors of *Pseudomonas aeruginosa* cystic fibrosis strains

CONTENTS

^{1}H, ^{13}C NMR and HPLC/MS spectra of compound **7a**

^{1}H, ^{13}C NMR and HPLC/MS spectra of compound **7b**

^{1}H, ^{13}C NMR and HPLC/MS spectra of compound **7c**

^{1}H, ^{13}C NMR and HPLC/MS spectra of compound **7d**

^{1}H, ^{13}C NMR and HPLC/MS spectra of compound **8a**

^{1}H, ^{13}C NMR and HPLC/MS spectra of compound **8b**

^{1}H, ^{13}C NMR and HPLC/MS spectra of compound **9**

^{1}H, ^{13}C NMR and HPLC/MS spectra of compound **10**

^{1}H, ^{13}C NMR and HPLC/MS spectra of compound **11a**

^{1}H, ^{13}C NMR and HPLC/MS spectra of compound **11b**

^{1}H, ^{13}C NMR and HPLC/MS spectra of compound **11c**

^{1}H, ^{13}C NMR and HPLC/MS spectra of compound **11d**

^{1}H, ^{13}C NMR and HPLC/MS spectra of compound **11e**

^{1}H, ^{13}C NMR and HPLC/MS spectra of compound **11f**

^{1}H, ^{13}C NMR and HPLC/MS spectra of compound **22a**

^{1}H, ^{13}C NMR and HPLC/MS spectra of compound **22b**

HRMS spectrum of compound **7a**

HRMS spectrum of compound **11a**

HRMS spectrum of compound **11b**

HRMS spectrum of compound **11c**

HRMS spectrum of compound **11d**

^{1}H, ^{13}C NMR and HPLC/MS spectra of compound 7a

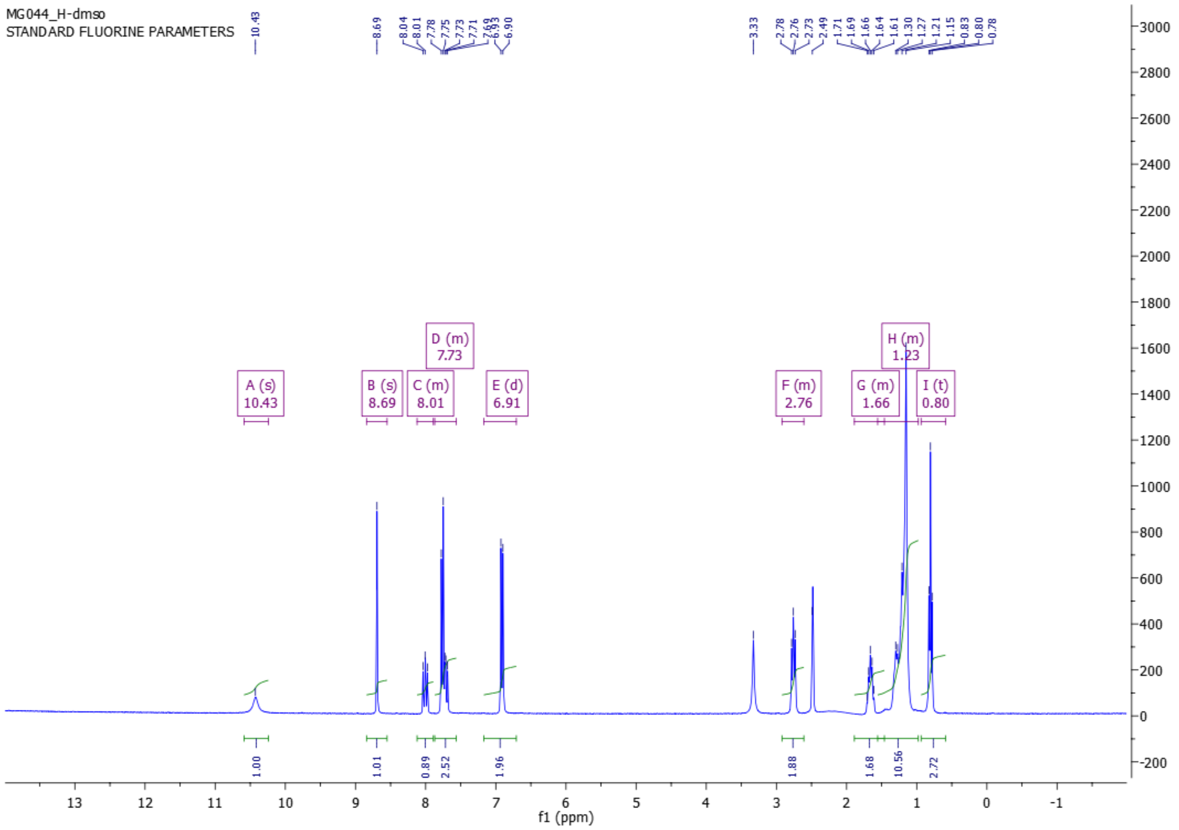

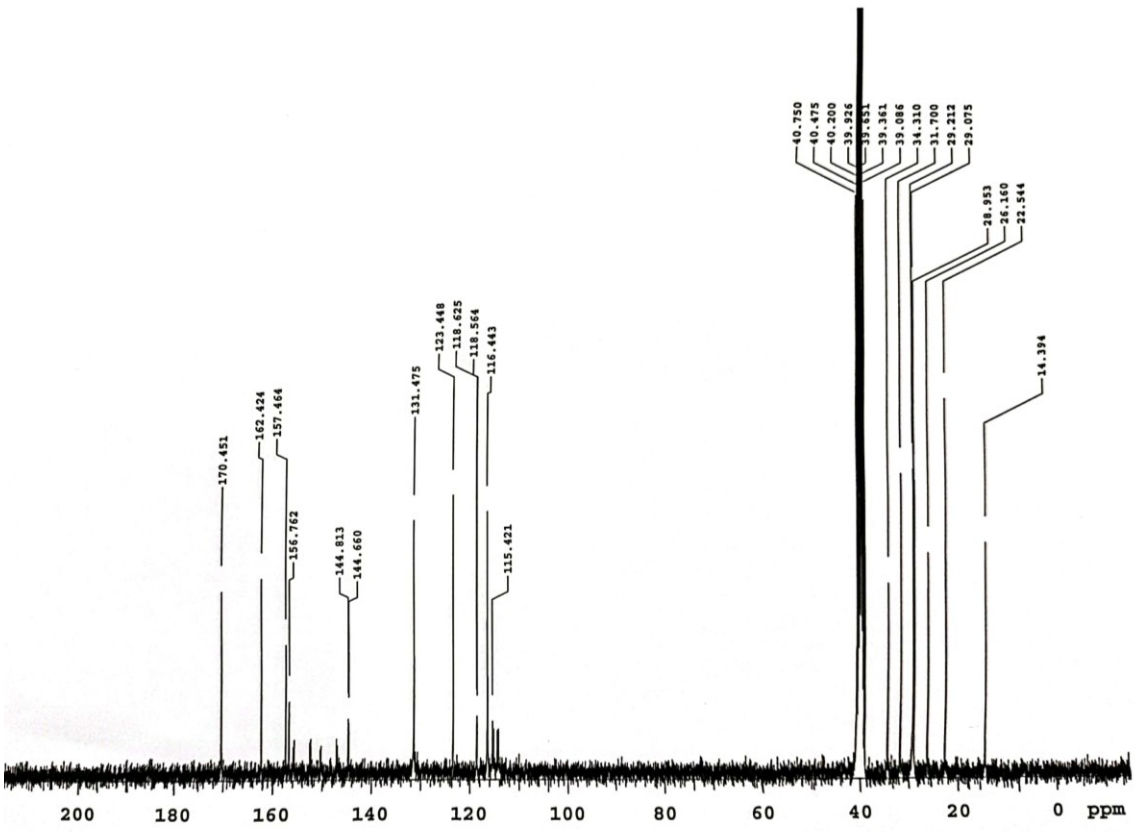

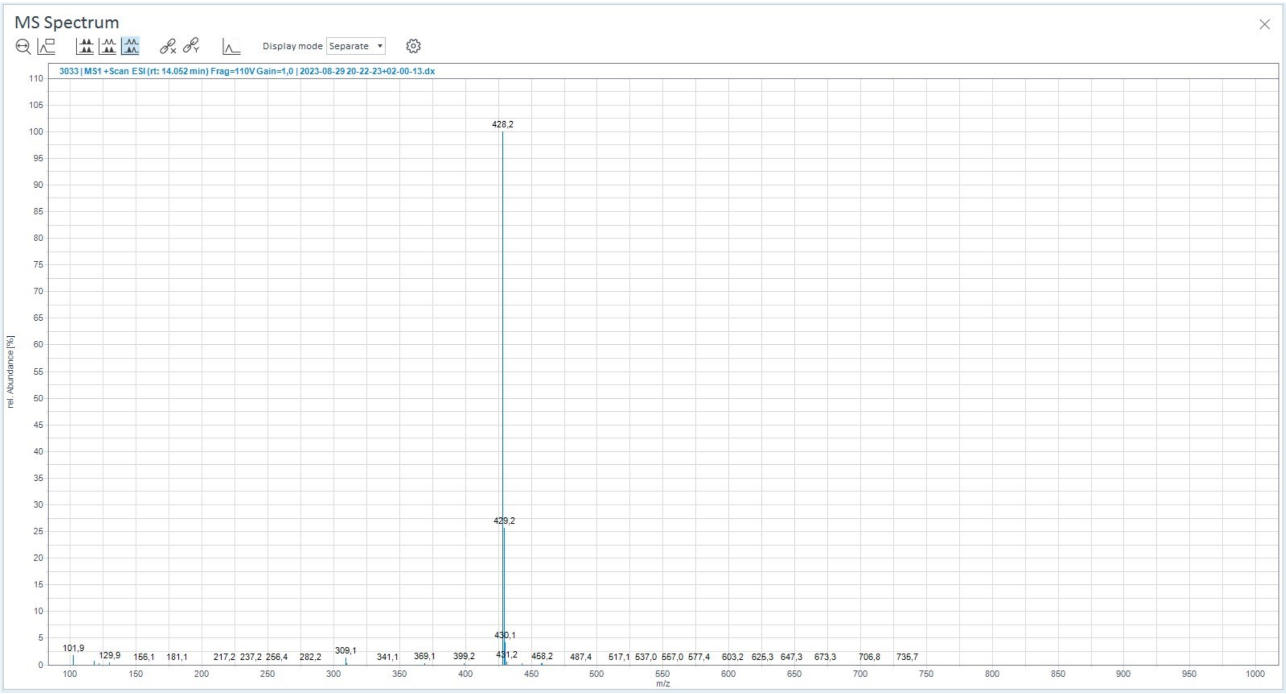

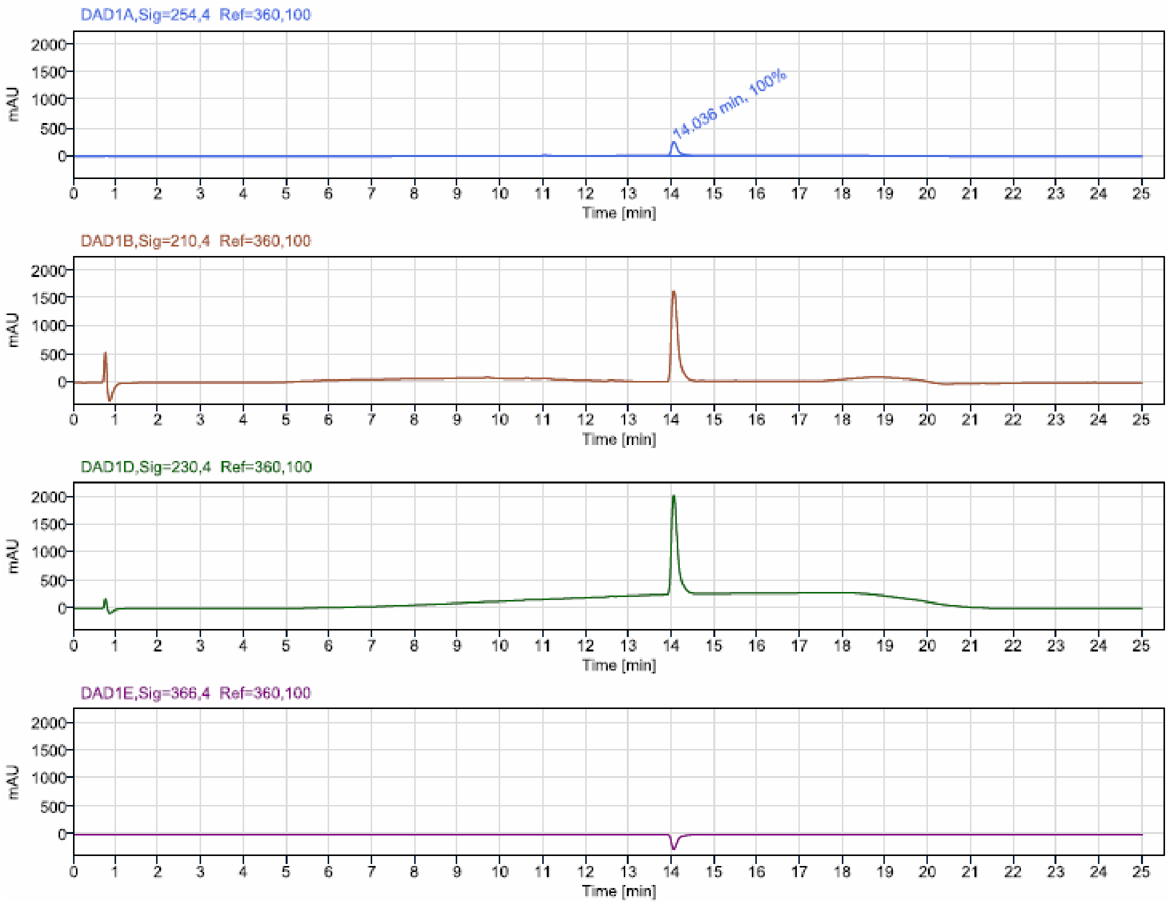

Peak Results (Area Percent at least 1%)

RT (min)	Signal Description	Width (min)	Area	Height	Area%
14.036	DAD1A,Sig=254,4 Ref=360,100	0.610	2456.4	246.2	100.00
14.056	MS1 +TIC SCAN ESI Frag=110V Gain=1.0	0.792	17266573.7	992387.3	100.00

^{1}H, ^{13}C NMR and HPLC/MS spectra of compound 7b

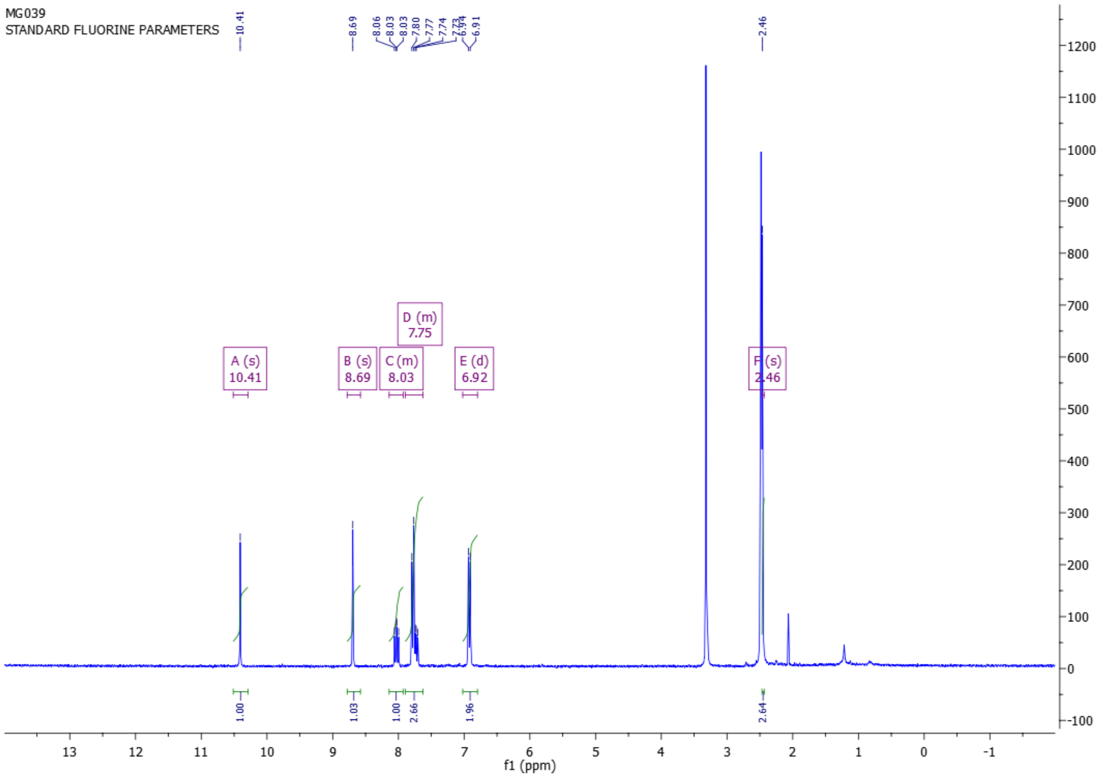

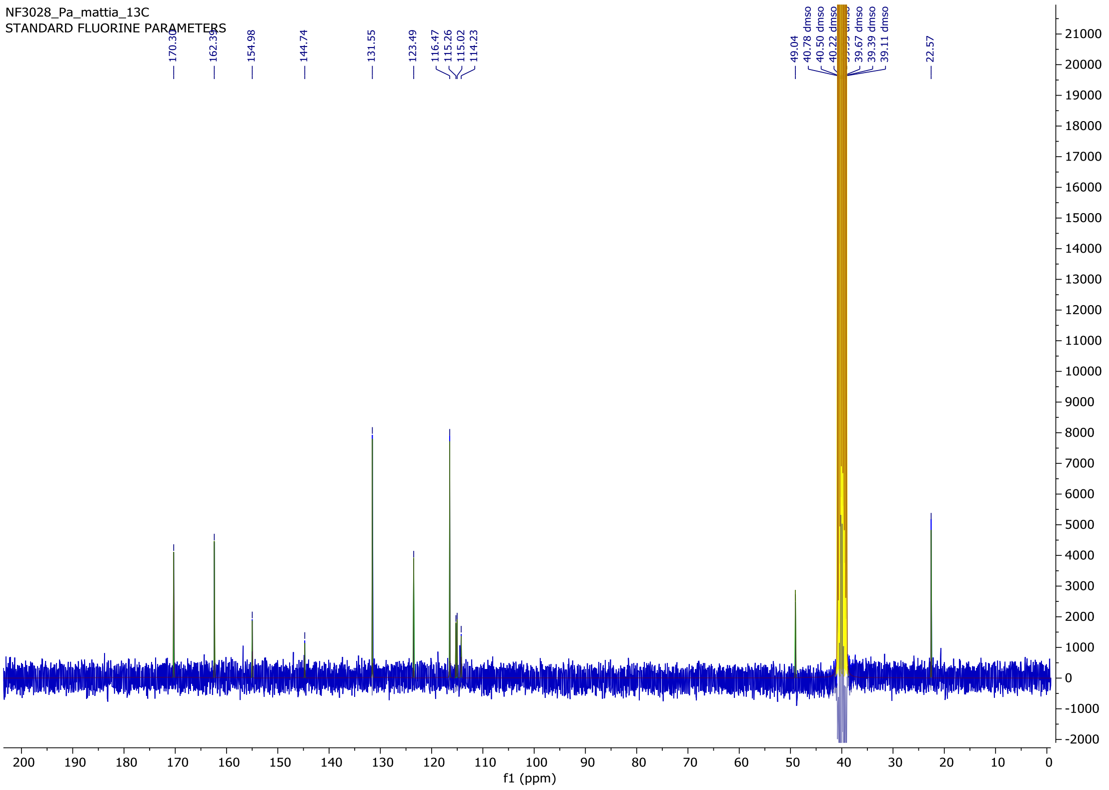

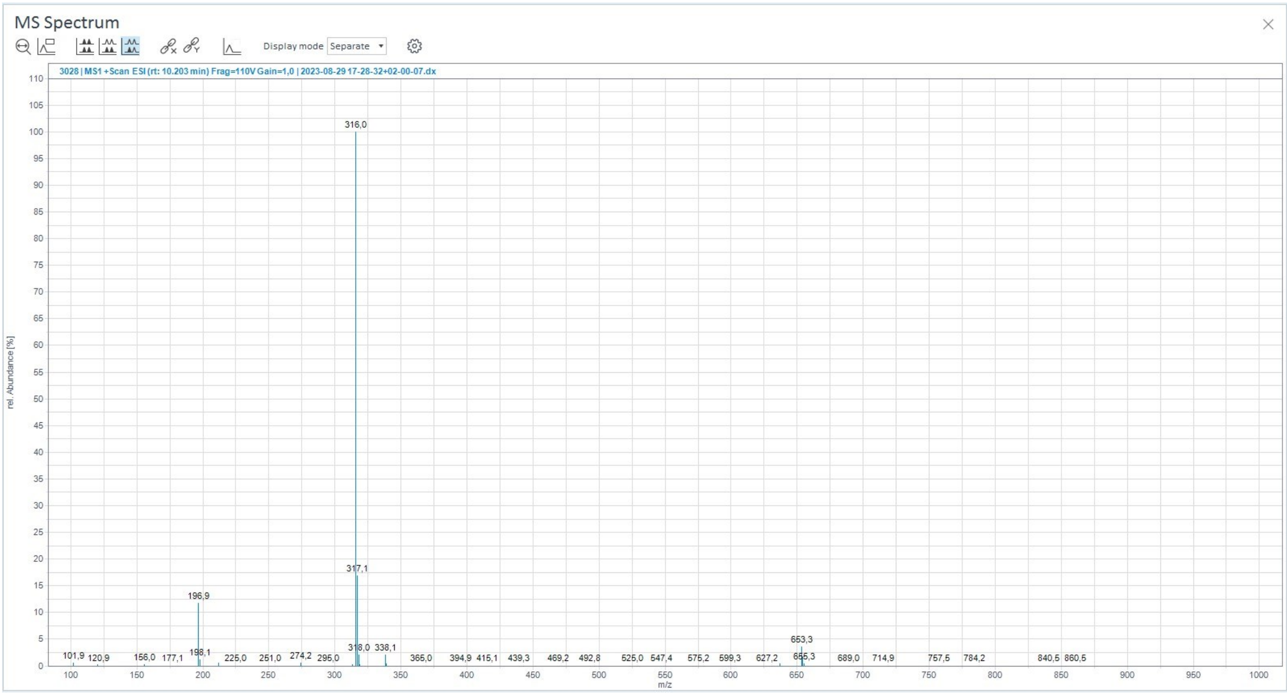

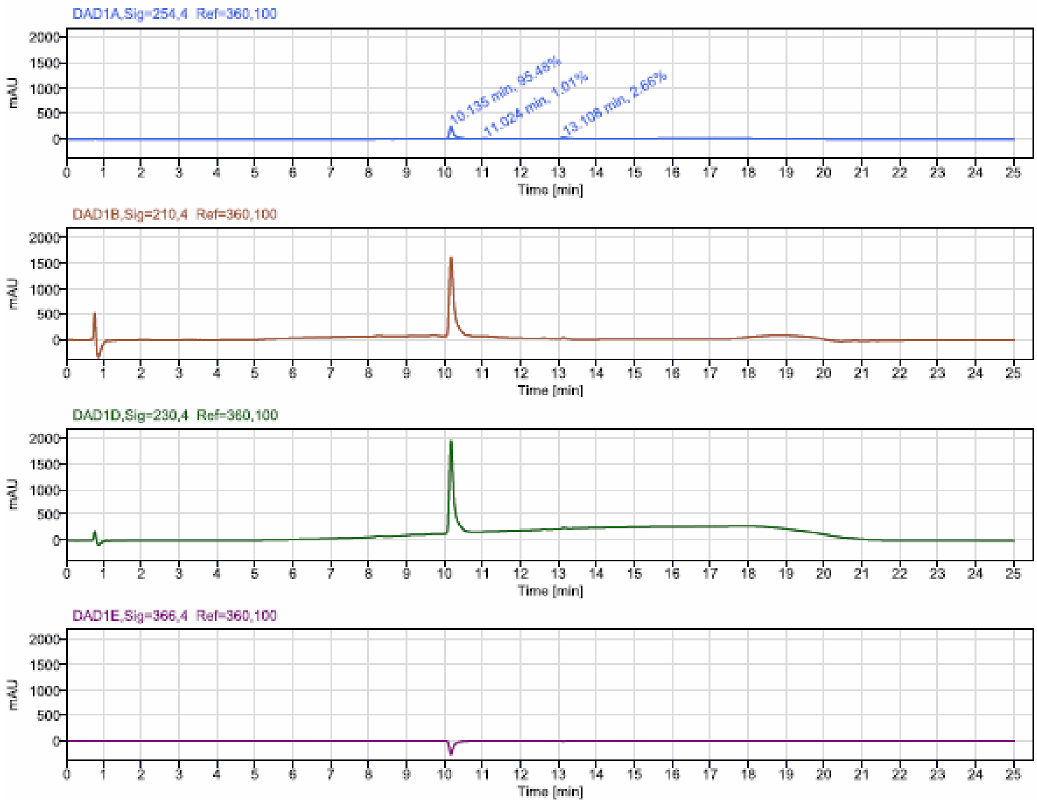

Peak Results (Area Percent at least 1%)

RT (min)	Signal Description	Width (min)	Area	Height	Area%
10,135	DAD1A,Sig=254,4 Ref=360,100	0,957	2041,4	241,0	95,48
10.203	MS1 +TIC SCAN ESI Frag=110V Gain=1.0	0.875	26574503.5	1687708.2	100.00
11,024	DAD1A,Sig=254,4 Ref=360,100	0,134	21,5	4,4	1,01
13,108	DAD1A,Sig=254,4 Ref=360,100	0,146	56,9	12,5	2,66

^{1}H, ^{13}C NMR and HPLC/MS spectra of compound 7c

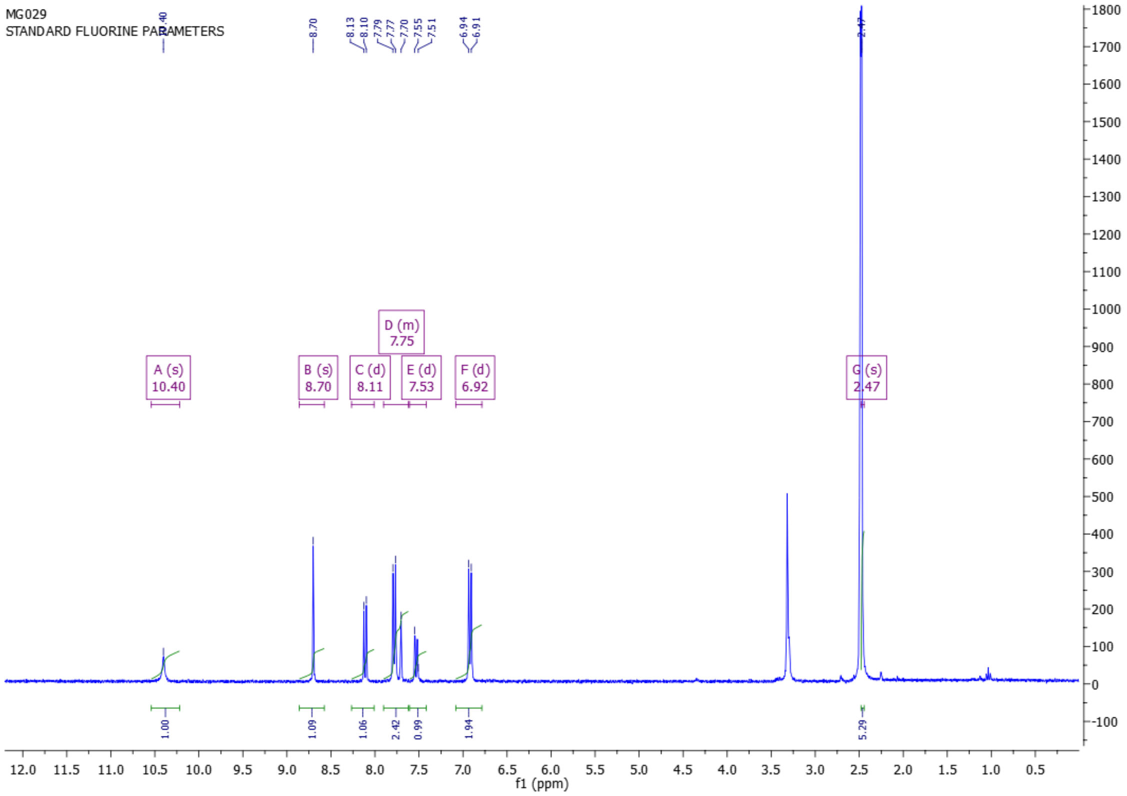

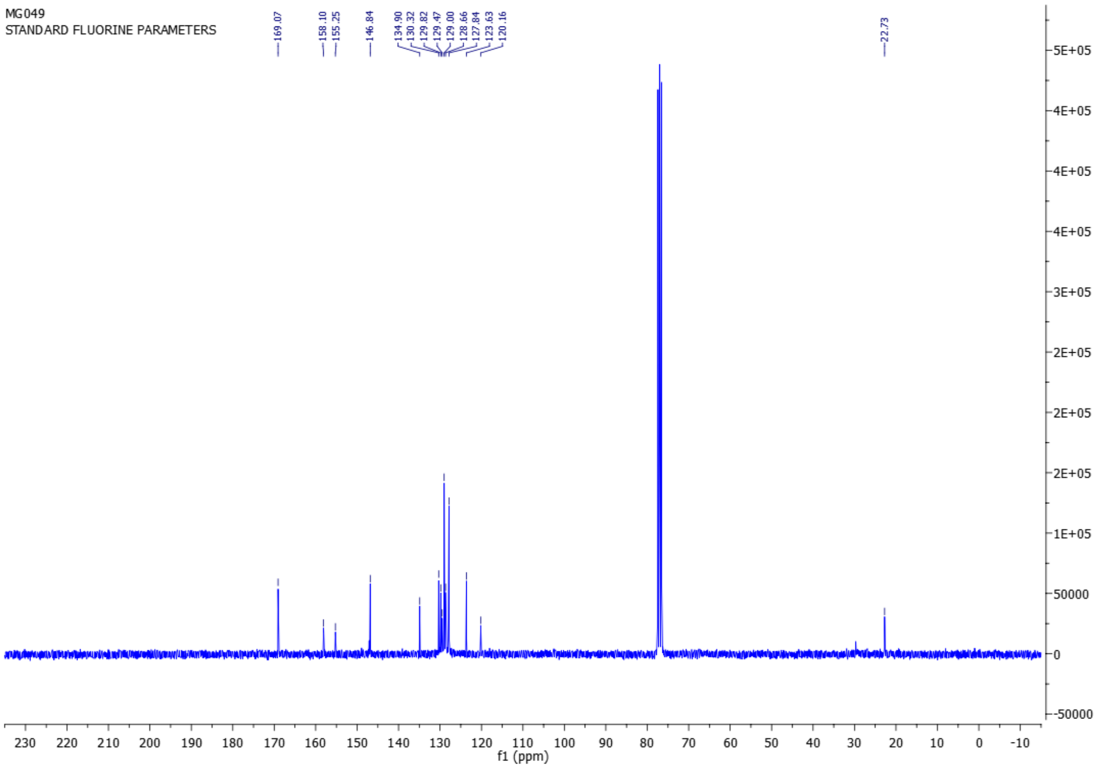

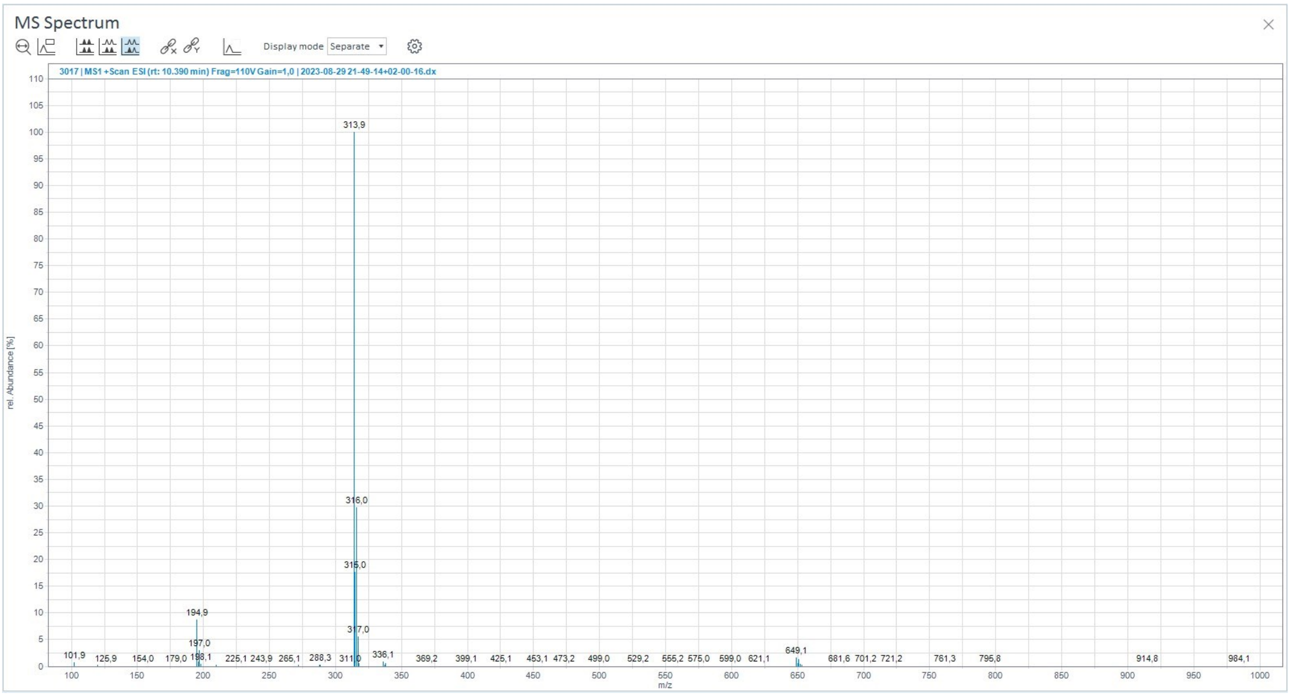

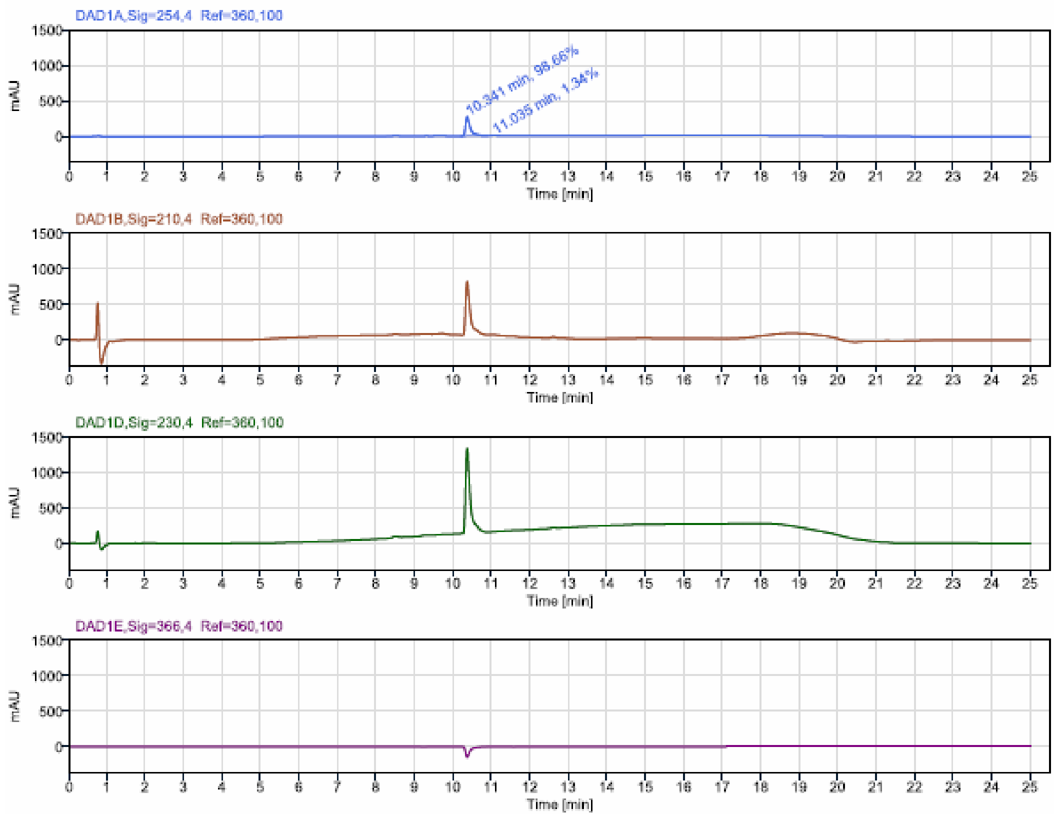

Peak Results (Area Percent at least 1%)

RT (min)	Signal Description	Width (min)	Area	Height	Area%
10,341	DAD1A,Sig=254,4 Ref=360,100	0,700	2307,3	272,2	98,66
10.398	MS1 +TIC SCAN ESI Frag=110V Gain=1.0	0.446	18242039.3	1526702.9	100.00
11.035	DAD1A,Sig=254,4 Ref=360,100	0.198	31.3	4.7	1.34

^{1}H, ^{13}C NMR and HPLC/MS spectra of compound 7d

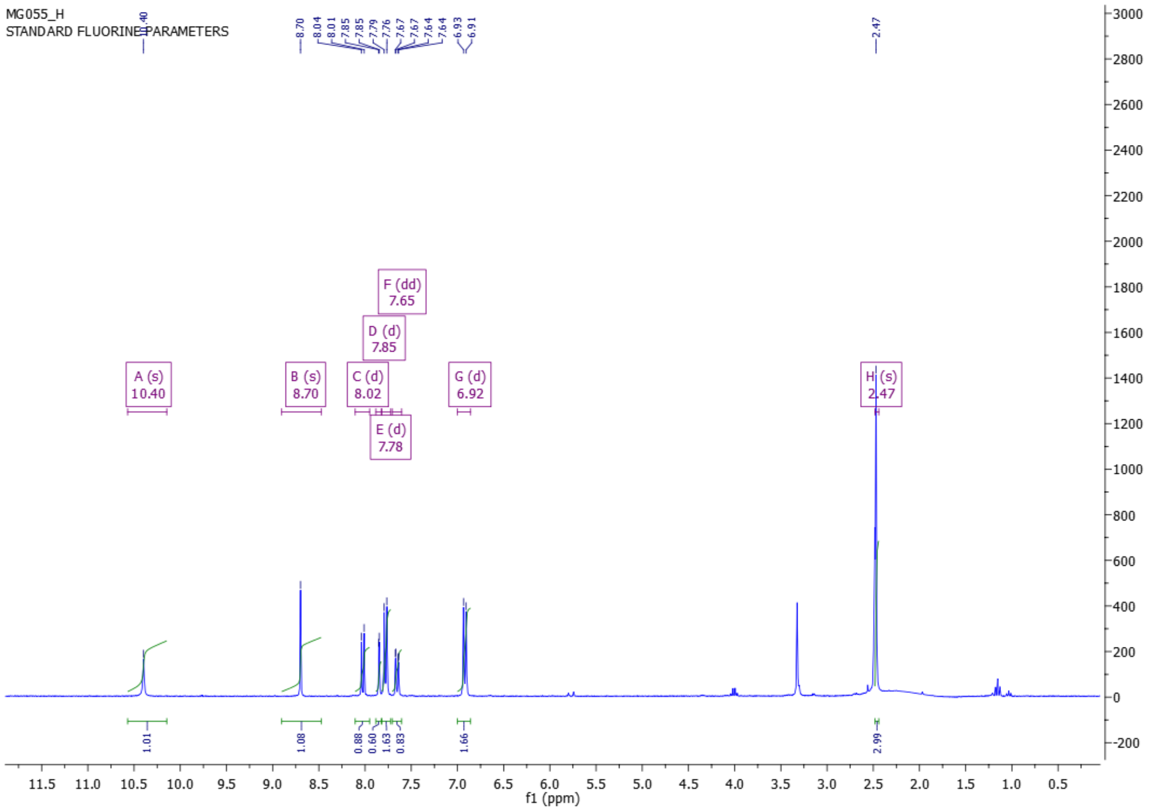

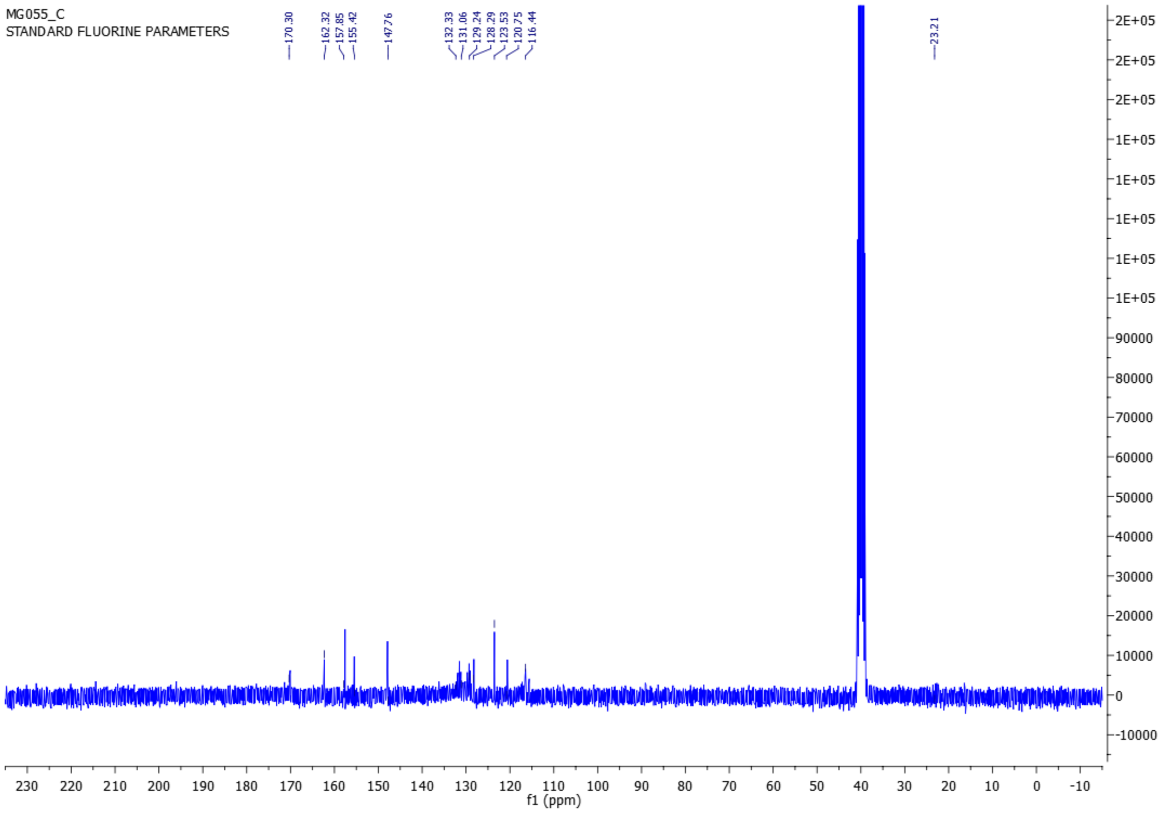

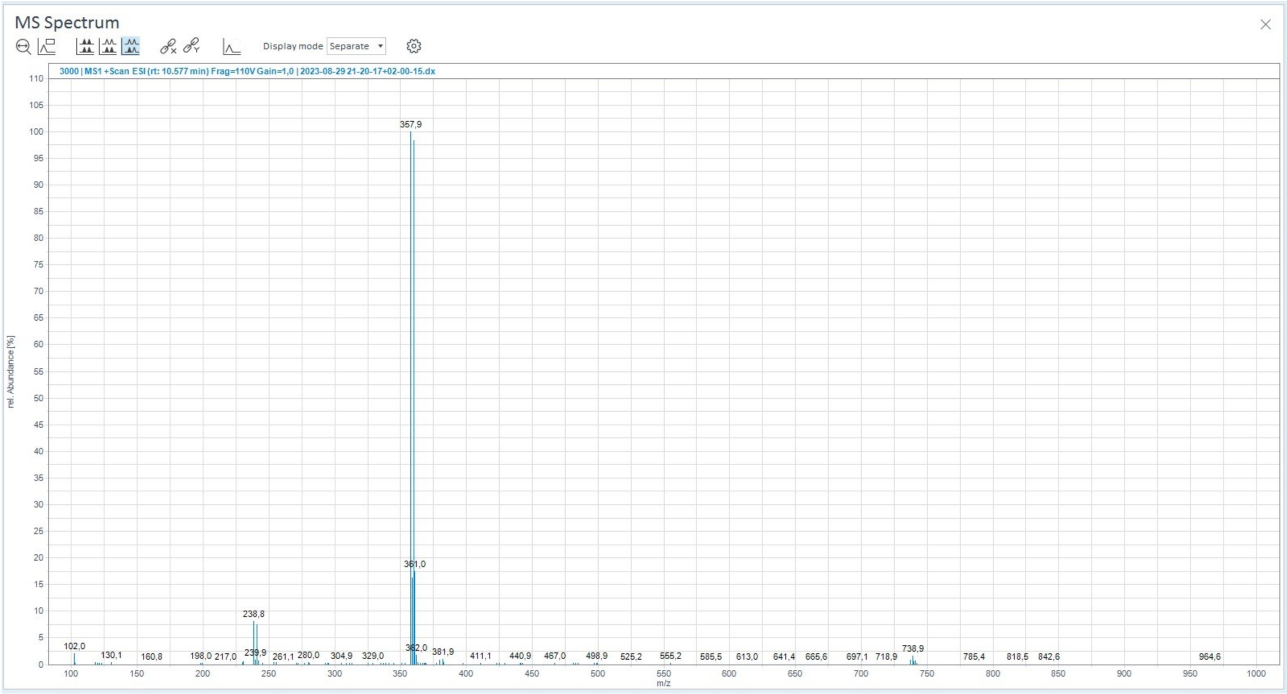

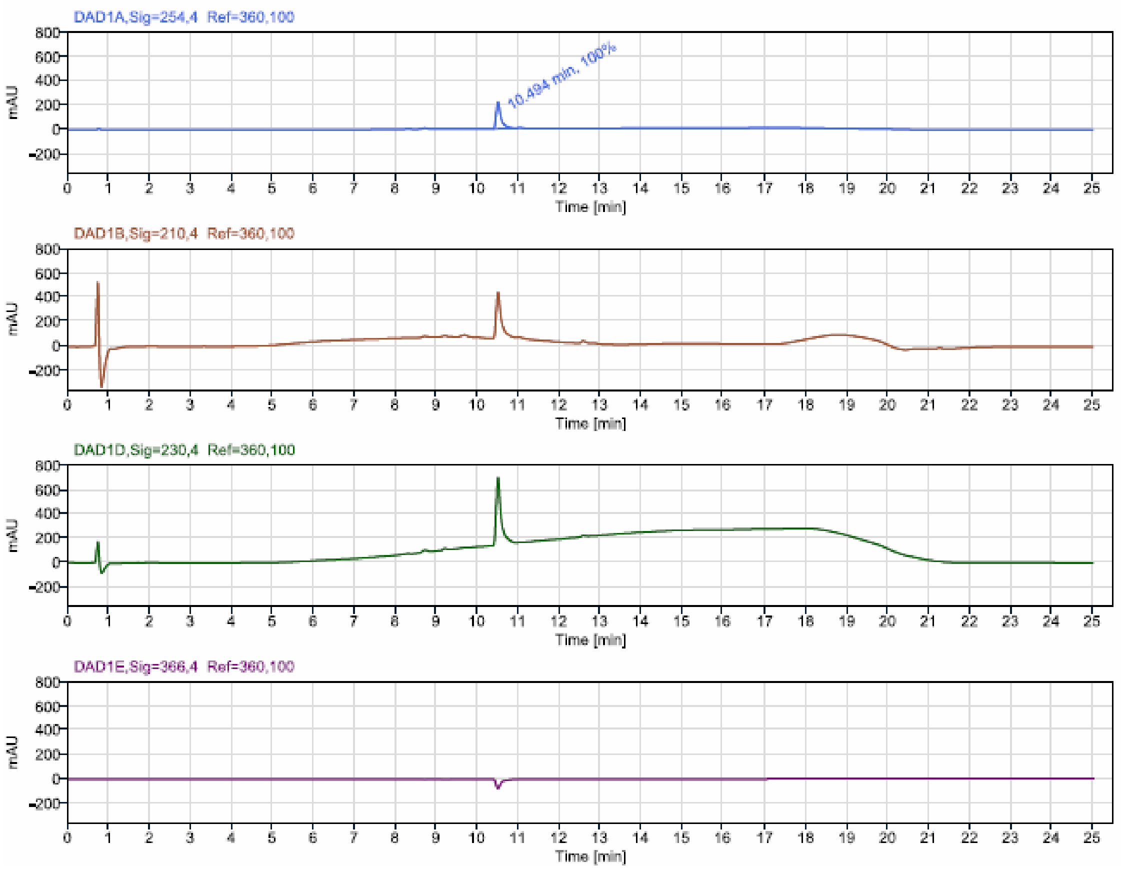

Peak Results (Area Percent at least 1%)

RT (min)	Signal Description	Width (min)	Area	Height	Area%
10,494	DAD1A,Sig=254,4 Ref=360,100	0,599	1748,6	216,8	100,00
10,571	MS1 +TIC SCAN ESI Frag=110V Gain=1,0	0,159	5198269,9	994096,1	100,00

^{1}H, ^{13}C NMR and HPLC/MS spectra of compound 8a

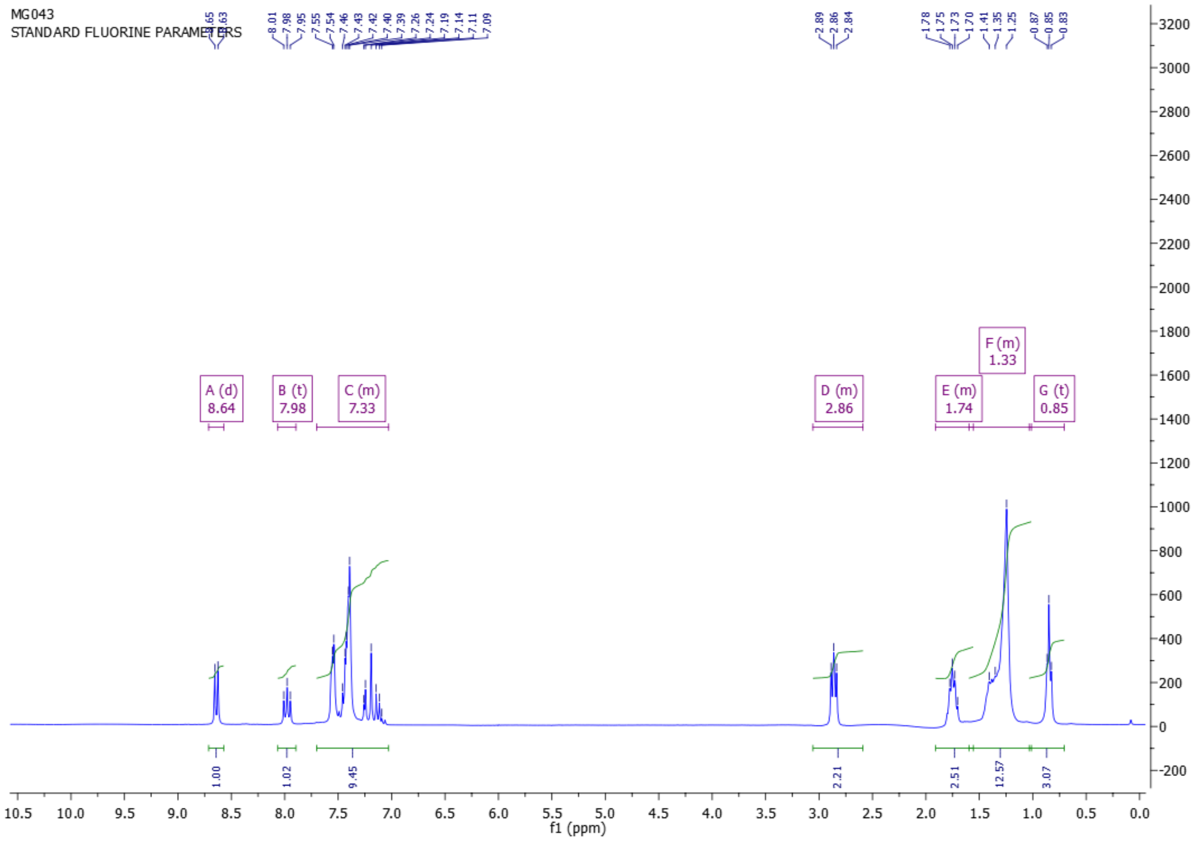

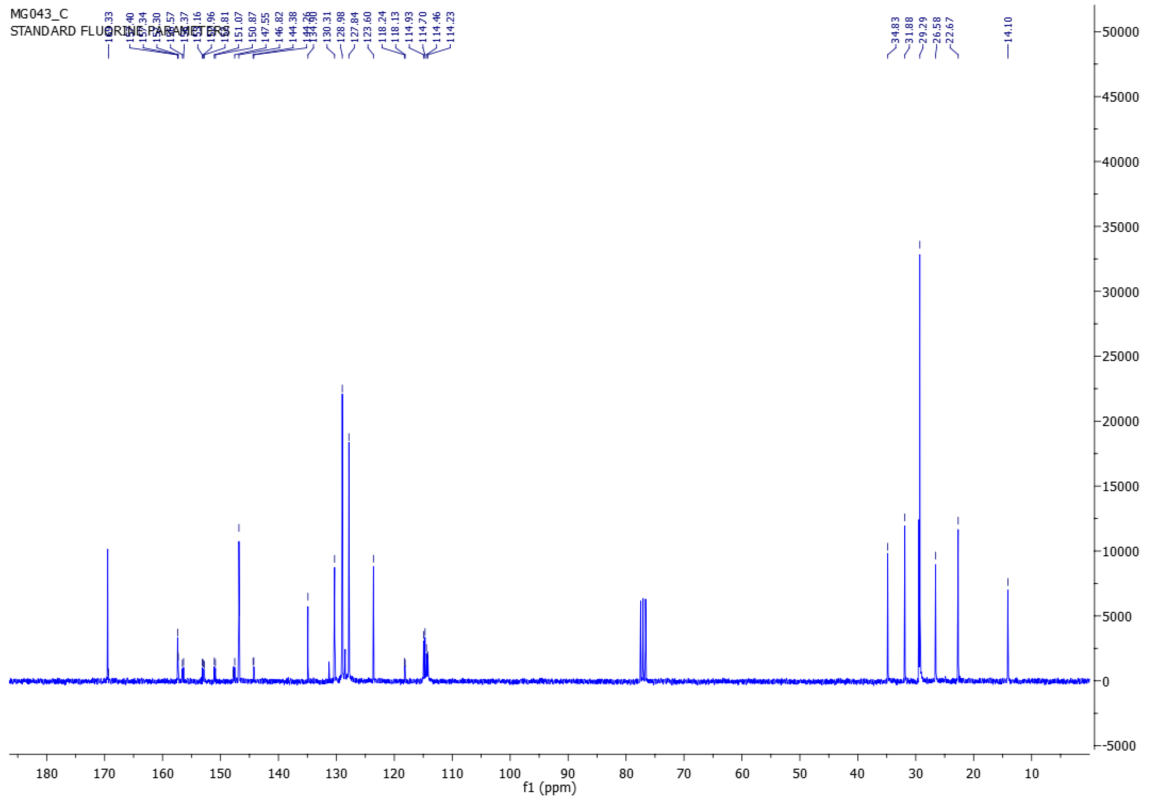

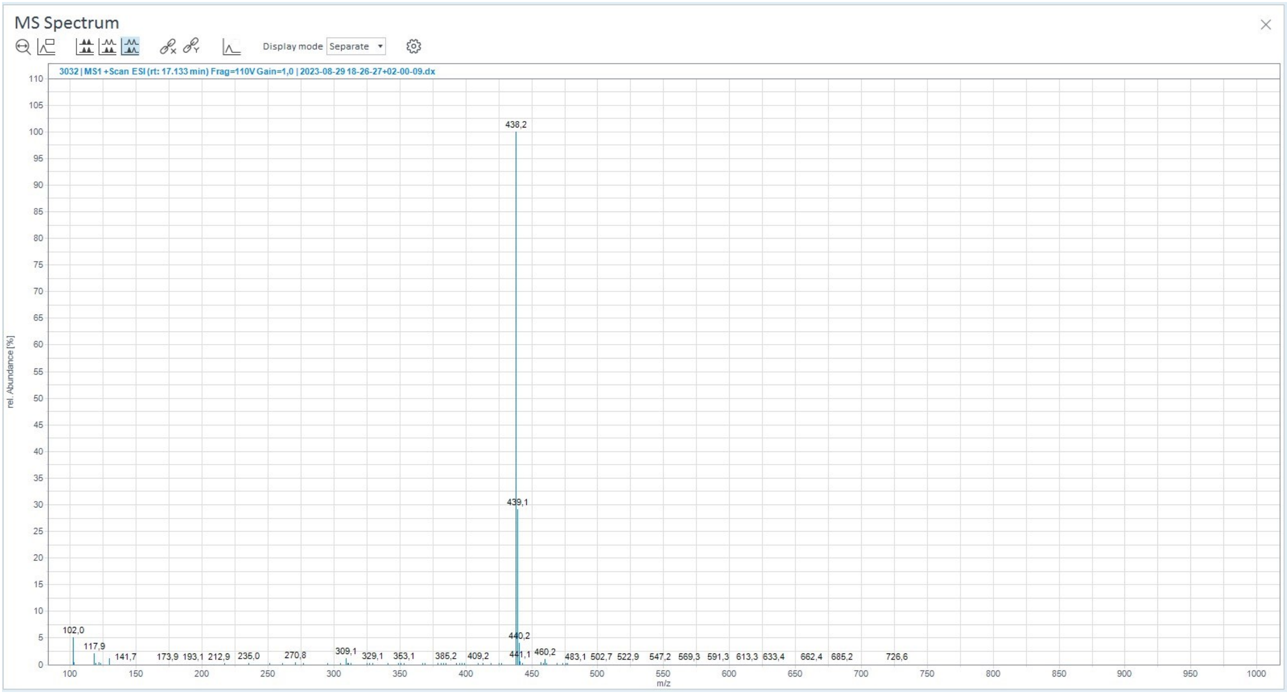

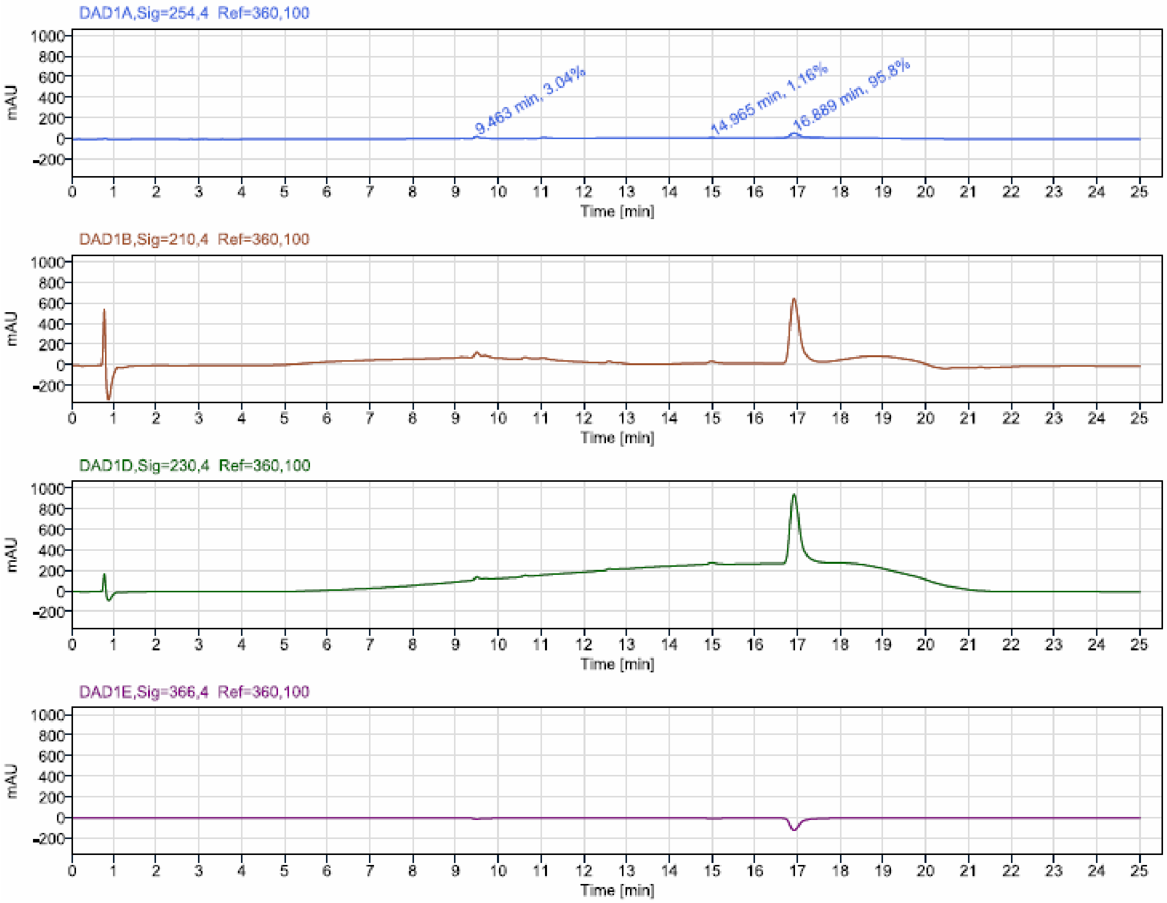

Peak Results (Area Percent at least 1%)

RT (min)	Signal Description	Width (min)	Area	Height	Area%
9.463	DAD1A,Sig=254,4 Ref=360,100	0.088	21.4	7.0	3.04
14.965	DAD1A,Sig=254,4 Ref=360,100	0.171	8.2	1.2	1.16
16,889	DAD1A,Sig=254,4 Ref=360,100	1,046	674,7	43,3	95,80
17.135	MS1 +TIC SCAN ESI Frag=110V Gain=1.0	0.563	7460209.7	319982.3	100.00

^1H, ^{13}C NMR and HPLC/MS spectra of compound 8b

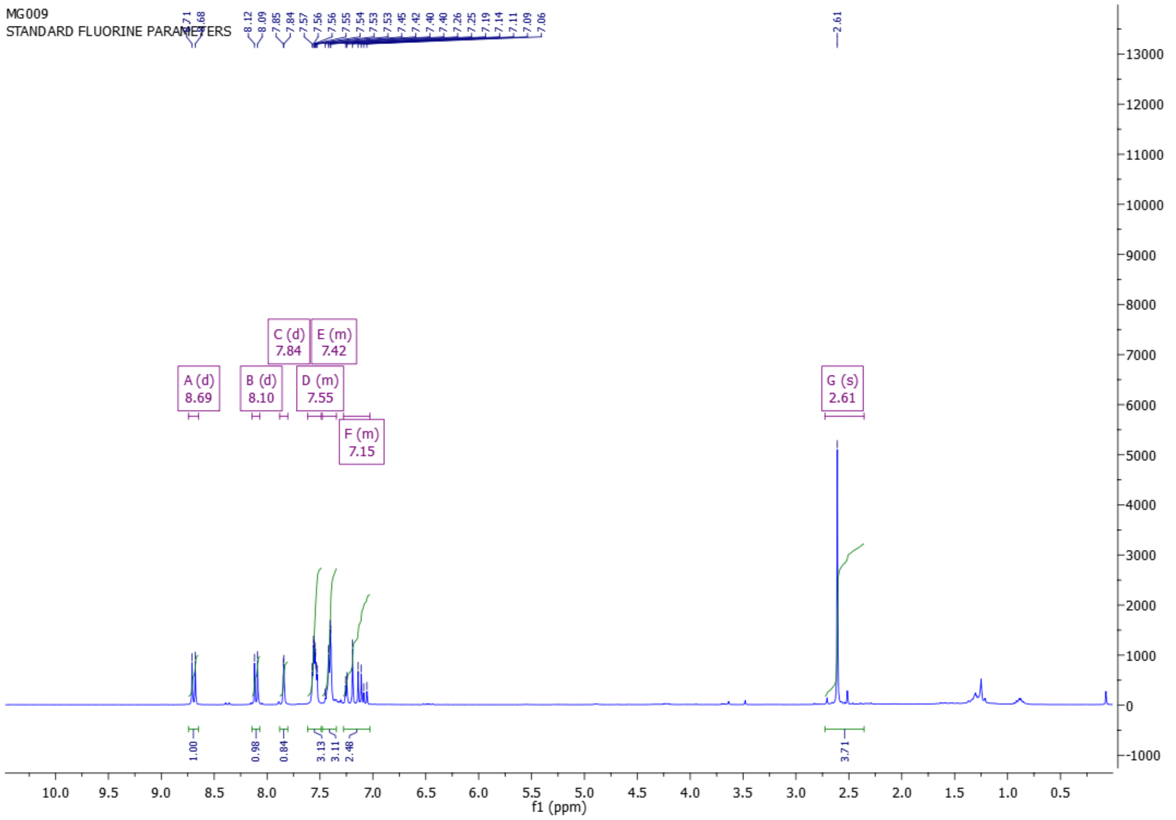

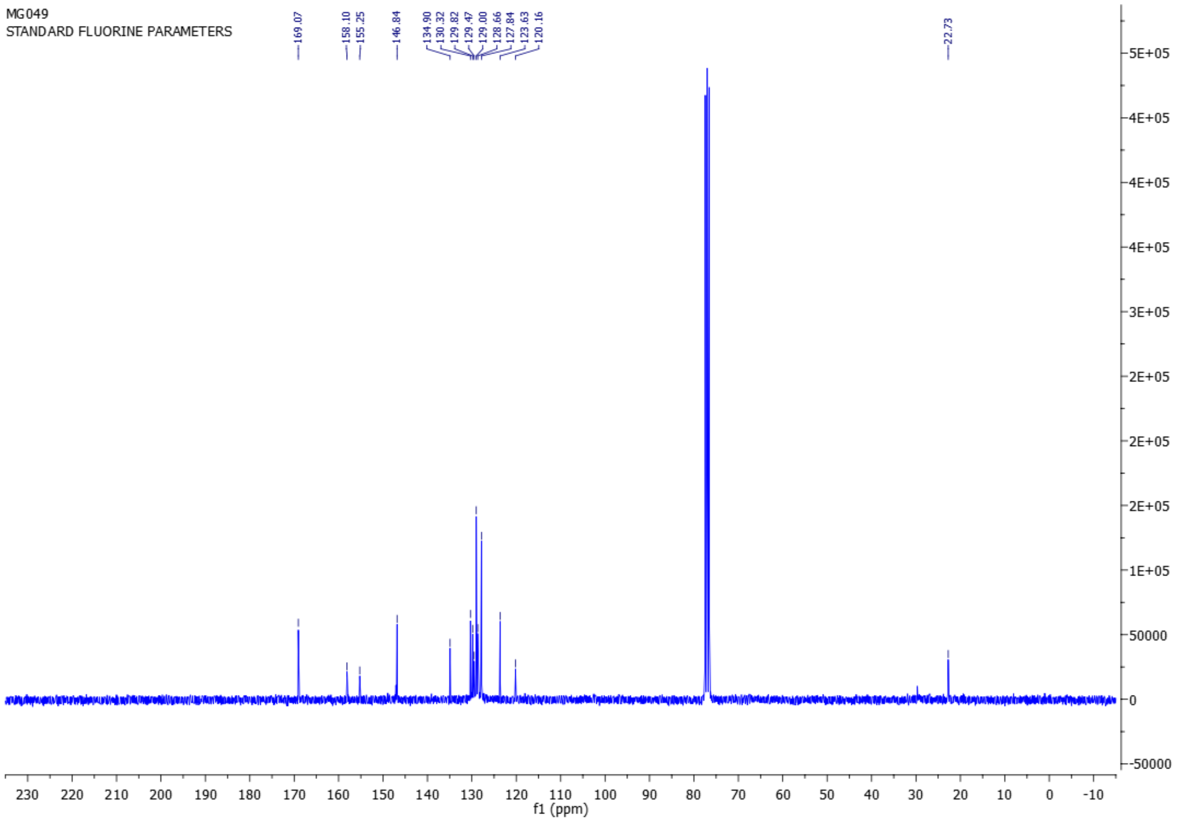

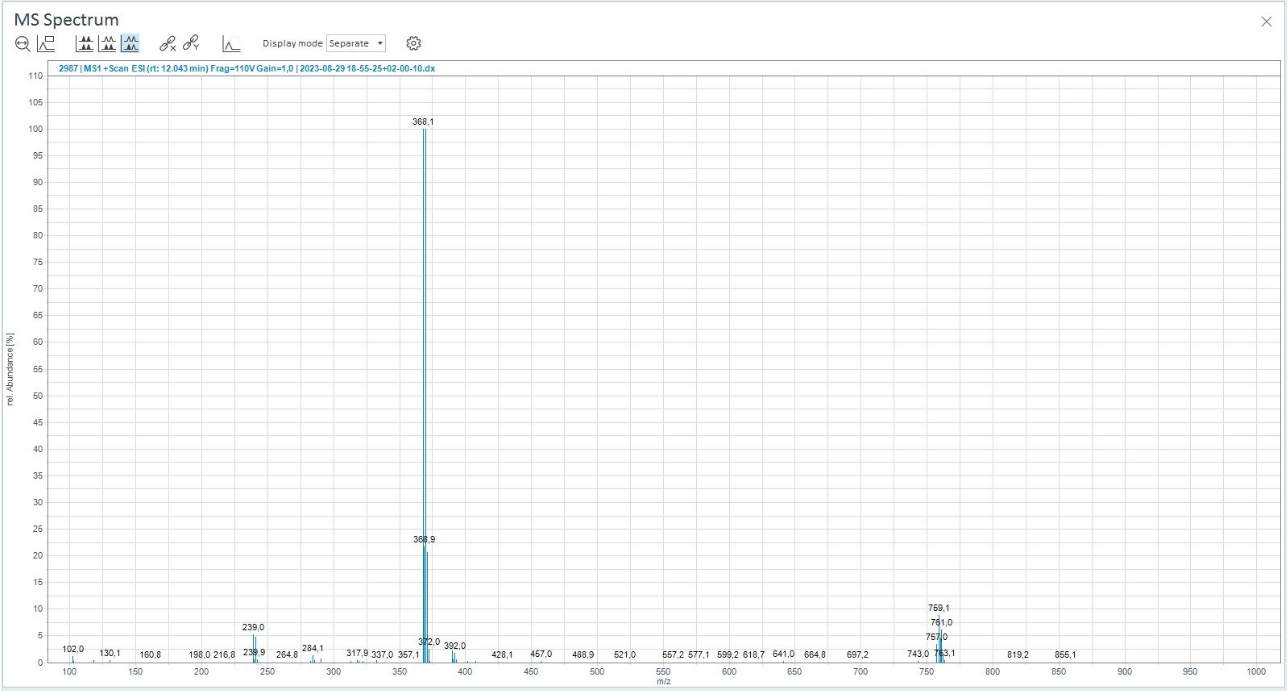

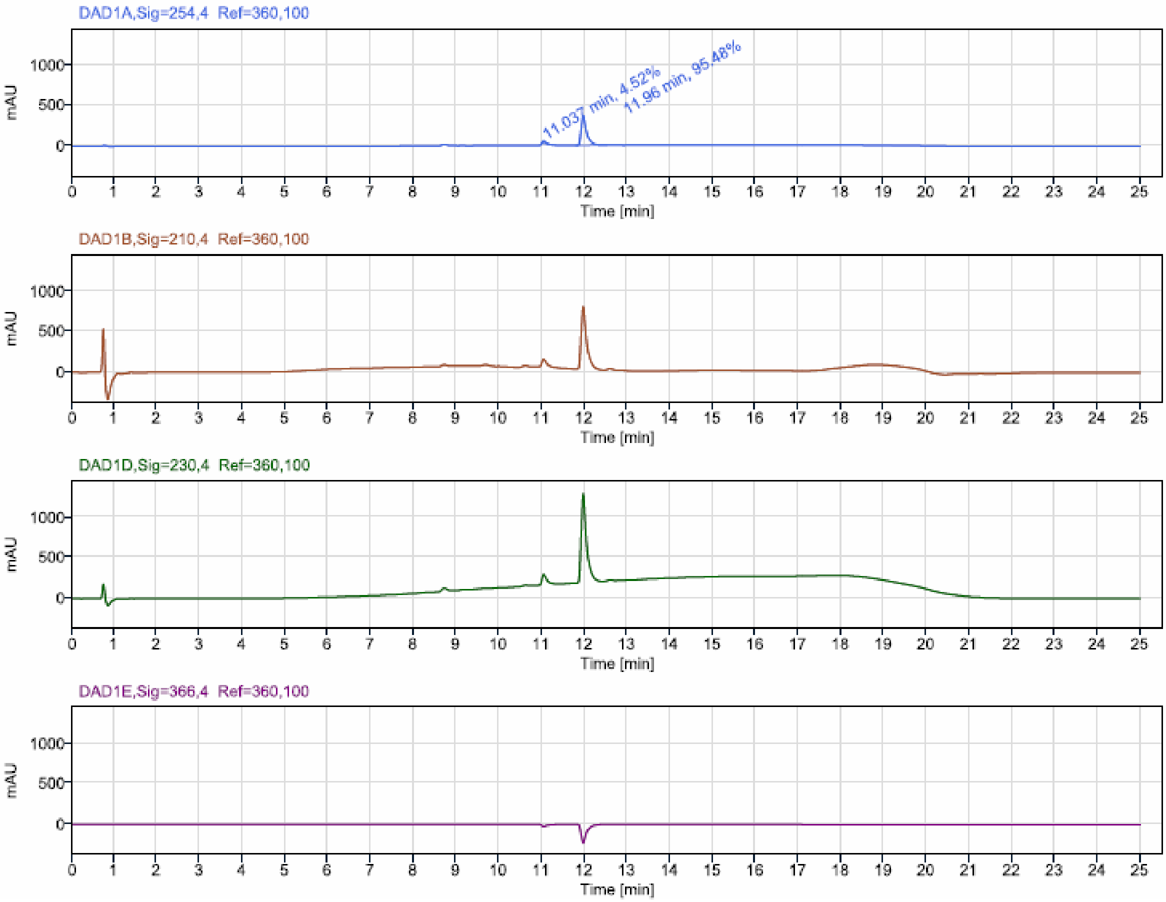

Peak Results (Area Percent at least 1%)

RT (min)	Signal Description	Width (min)	Area	Height	Area%
11.037	DAD1A,Sig=254,4 Ref=360,100	0.158	153.6	31.0	4.52
11.960	DAD1A,Sig=254,4 Ref=360,100	0.746	3241.3	364.8	95.48
12,040	MS1 +TIC SCAN ESI Frag=110V Gain=1,0	0,698	25124082,9	1714932,0	100,00

^{1}H, ^{13}C NMR and HPLC/MS spectra of compound 9

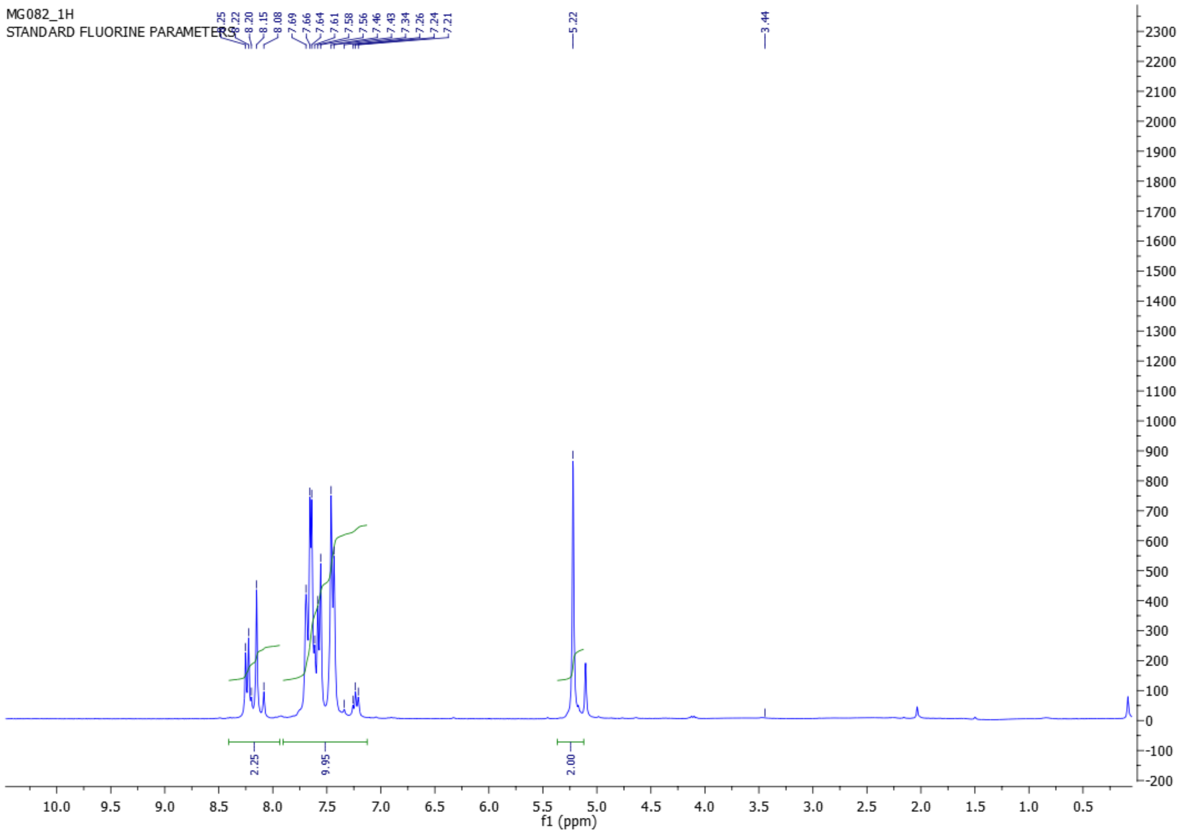

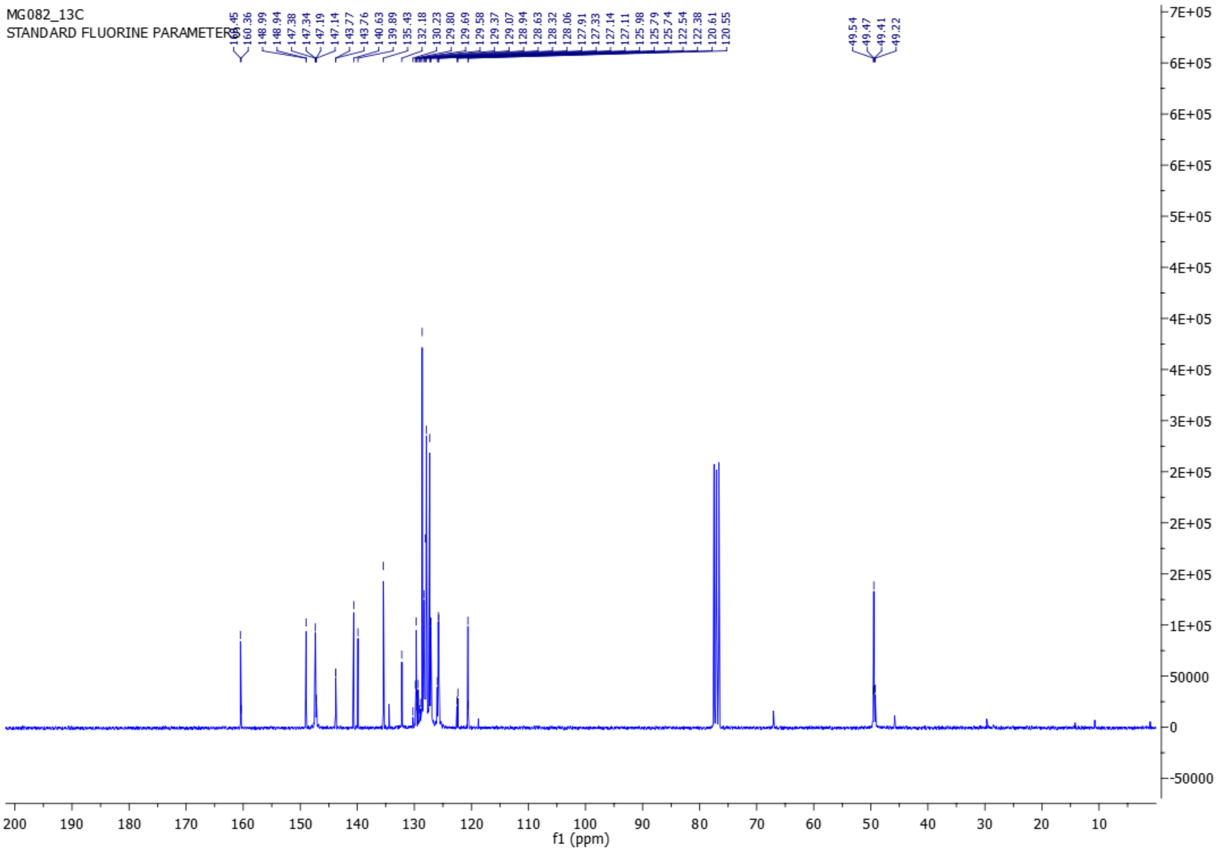

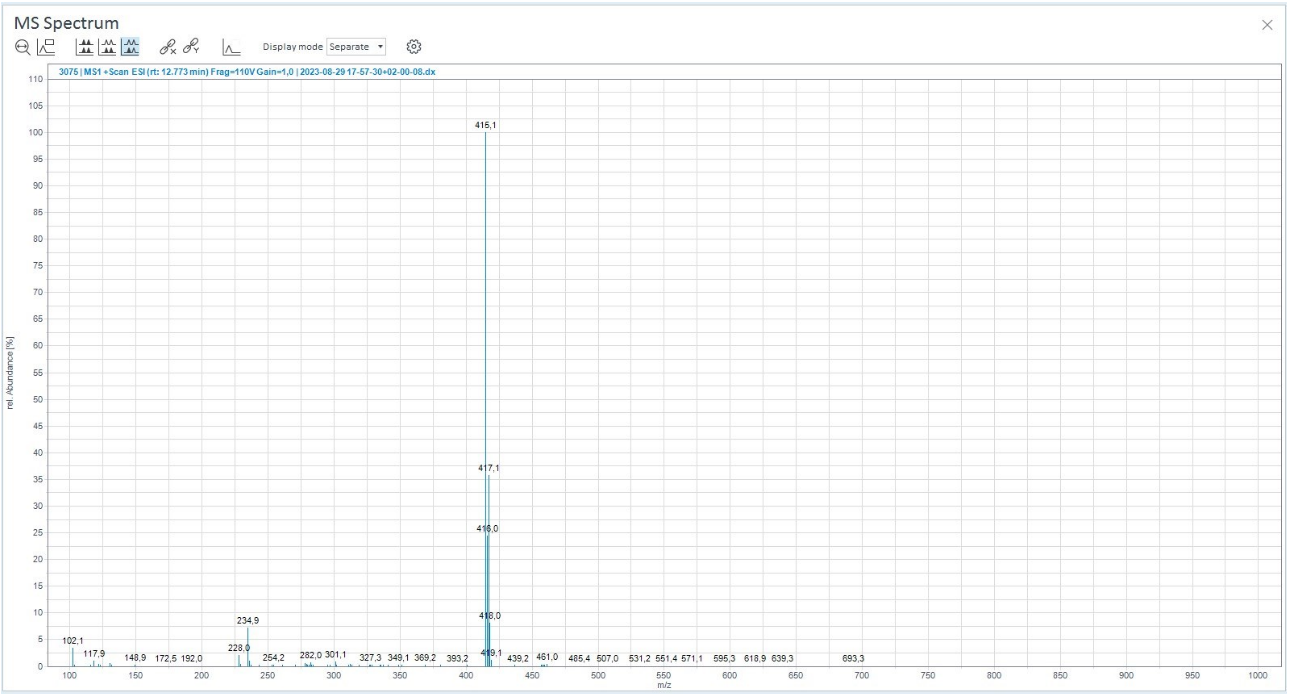

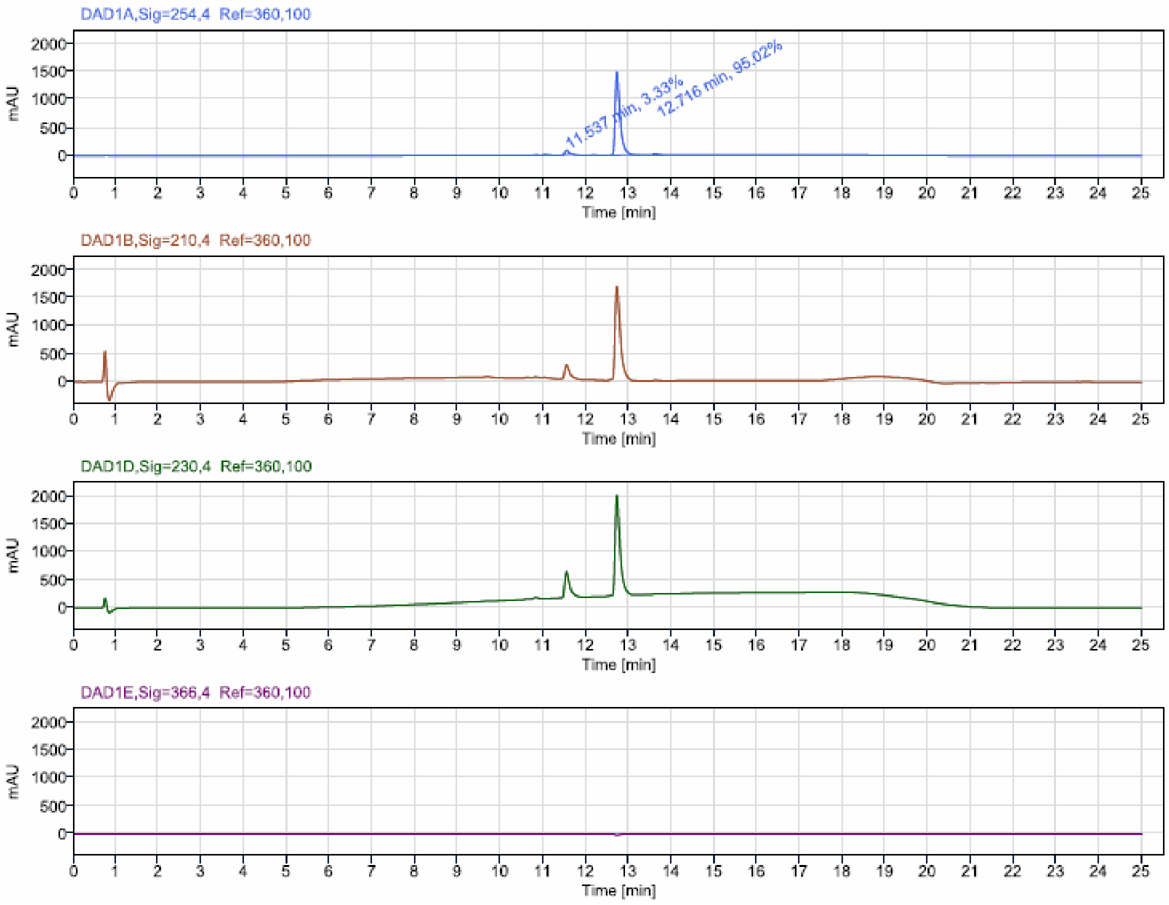

Peak Results (Area Percent at least 1%)

RT (min)	Signal Description	Width (min)	Area	Height	Area%
11.537	DAD1A,Sig=254,4 Ref=360,100	0.187	421.6	71.6	3.33
12.716	DAD1A,Sig=254,4 Ref=360,100	0.720	12036.0	1486.7	95.02
12,781	MS1 +TIC SCAN ESI Frag=110V Gain=1,0	0,580	9926186,2	876734,5	100,00

^{1}H, ^{13}C NMR and HPLC/MS spectra of compound 10

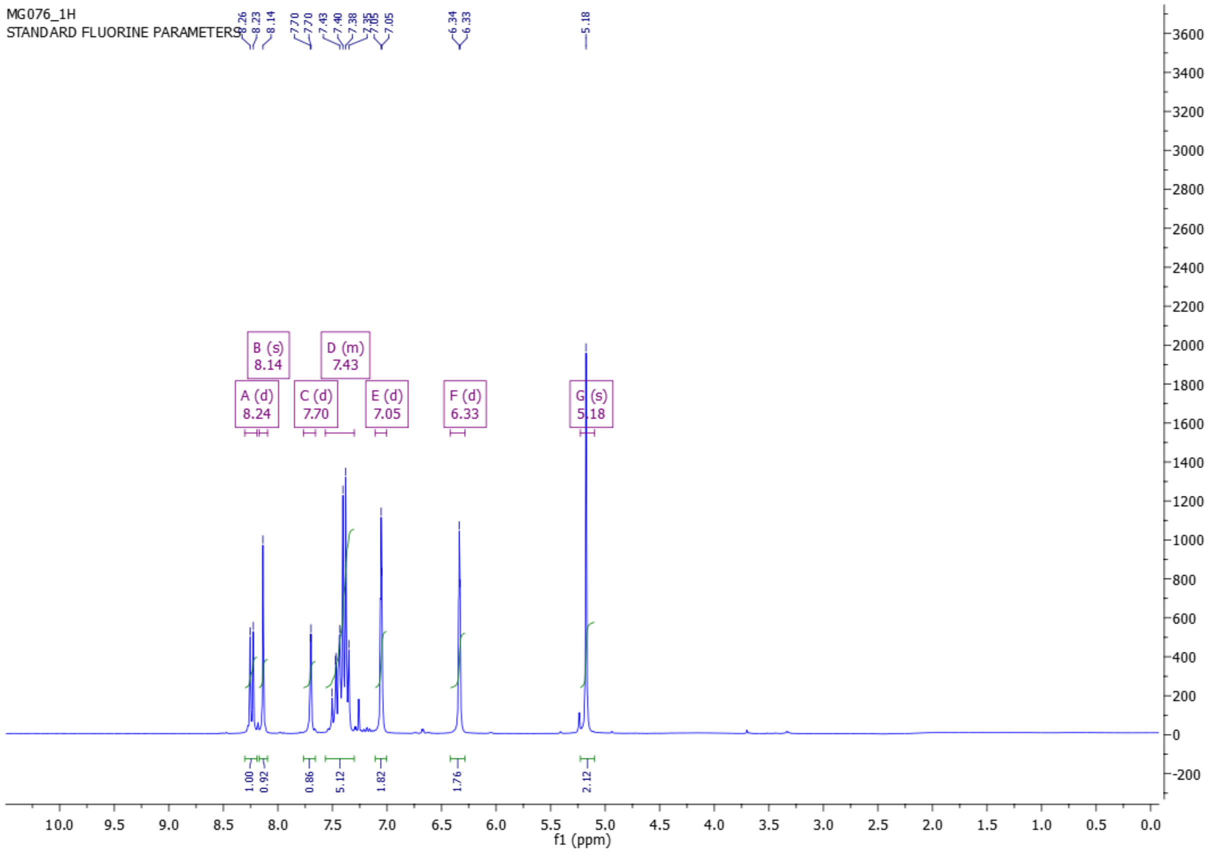

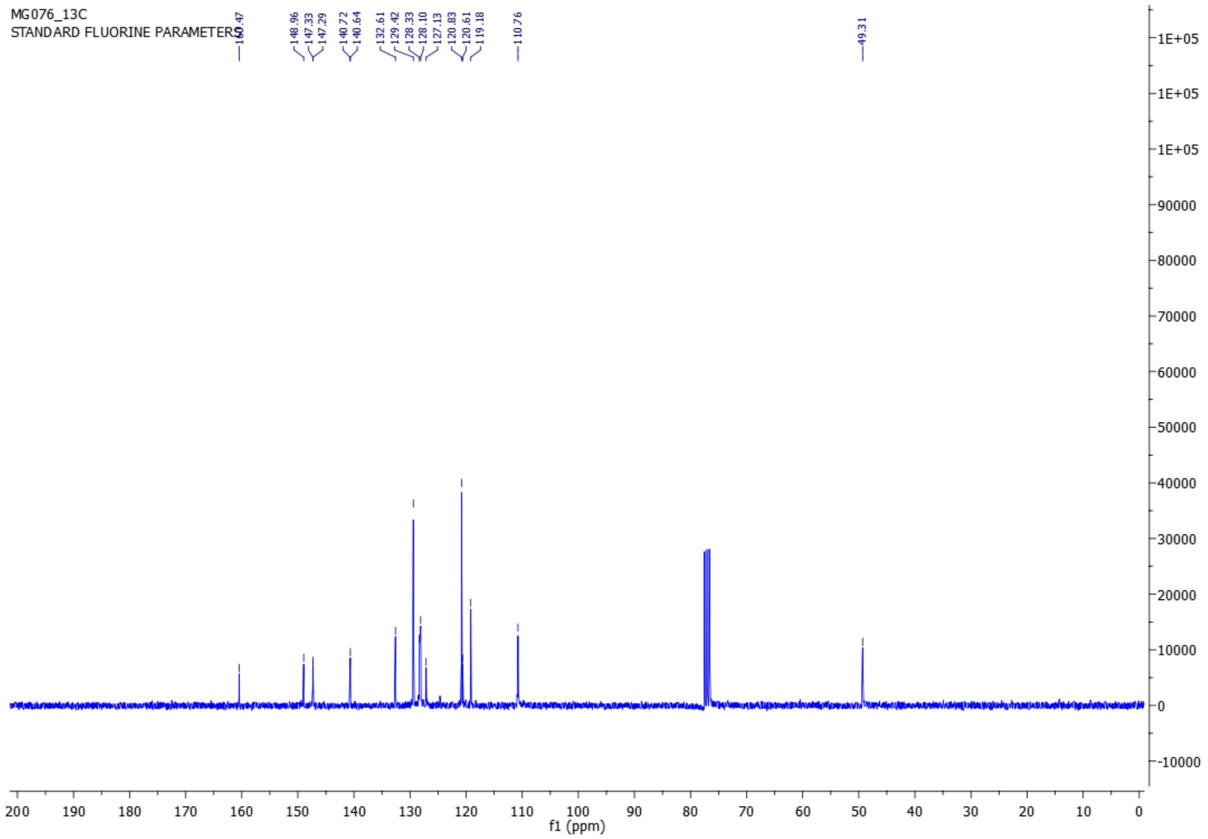

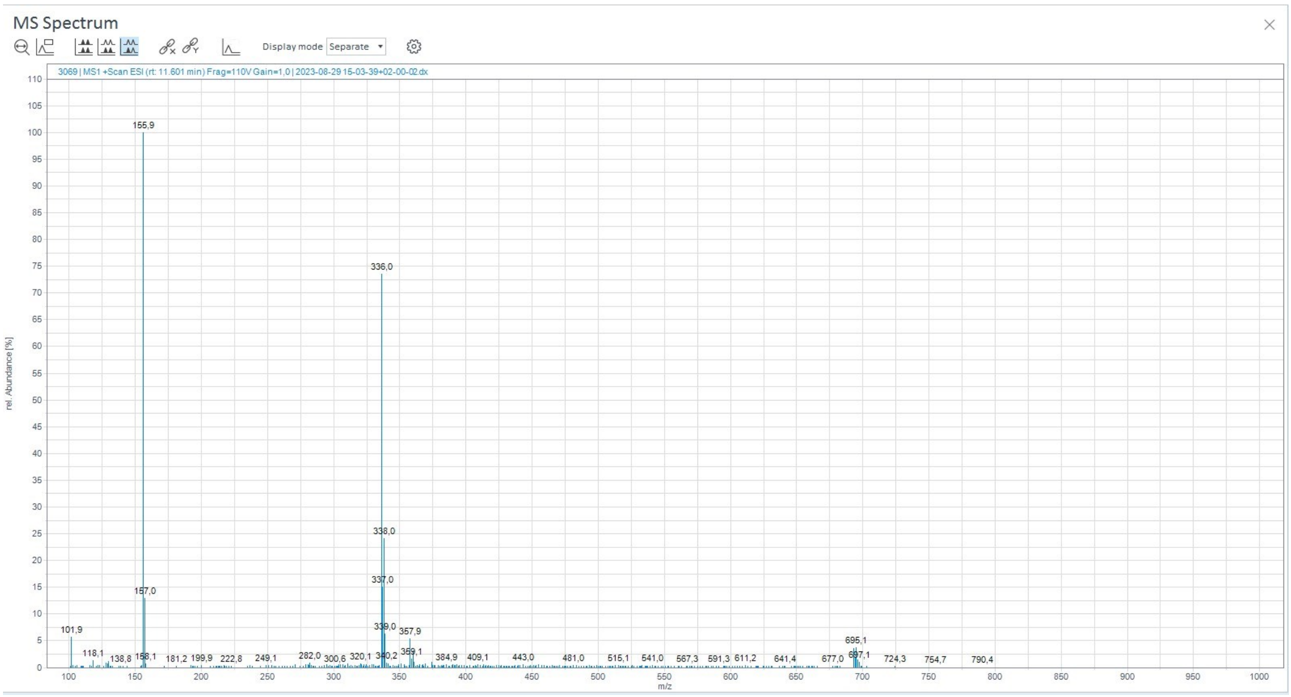

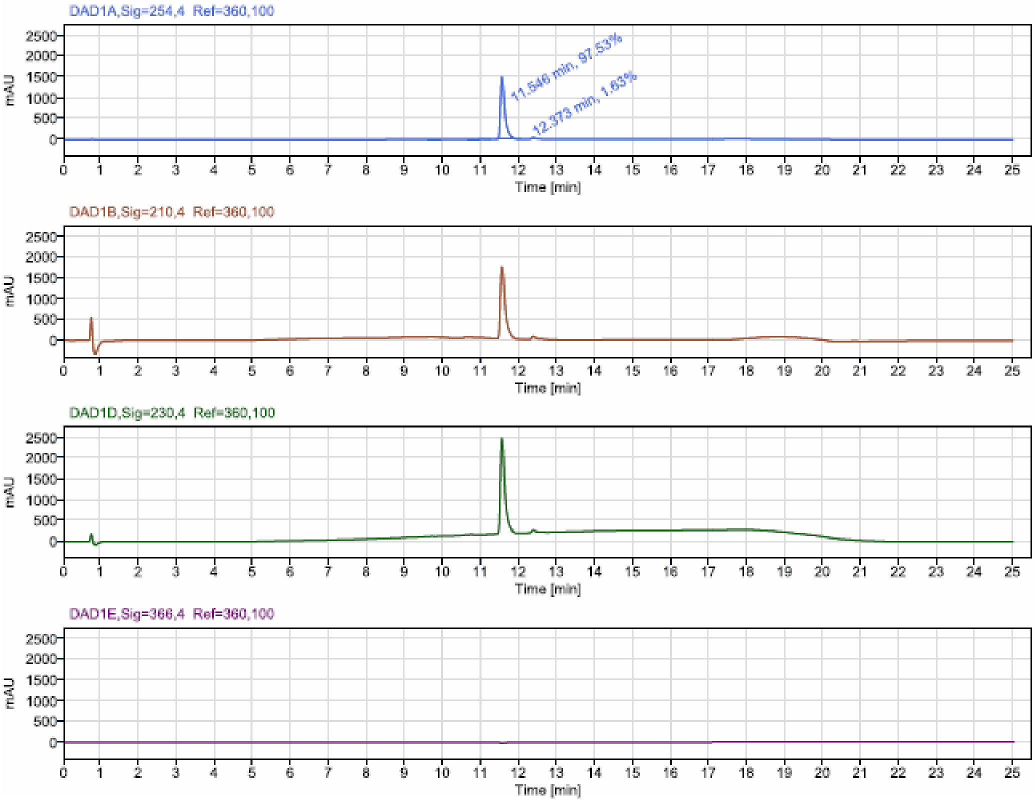

Peak Results (Area Percent at least 1%)

RT (min)	Signal Description	Width (min)	Area	Height	Area%
11,546	DAD1A,Sig=254,4 Ref=360,100	0,600	12079,4	1498,3	97,53
11,598	MS1 +TIC SCAN ESI Frag=110V Gain=1,0	0,309	4466372,3	433691,7	100,00
12,373	DAD1A,Sig=254,4 Ref=360,100	0,198	202,4	32,6	1,63

^{1}H, ^{13}C NMR and HPLC/MS spectra of compound 11a

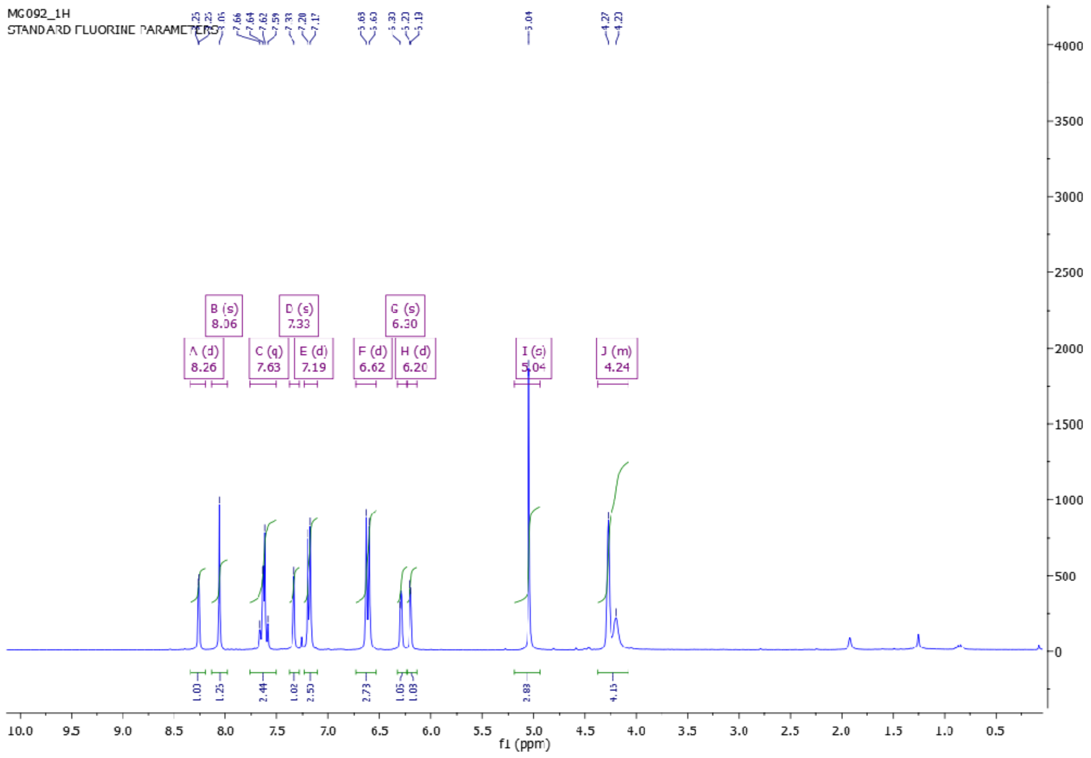

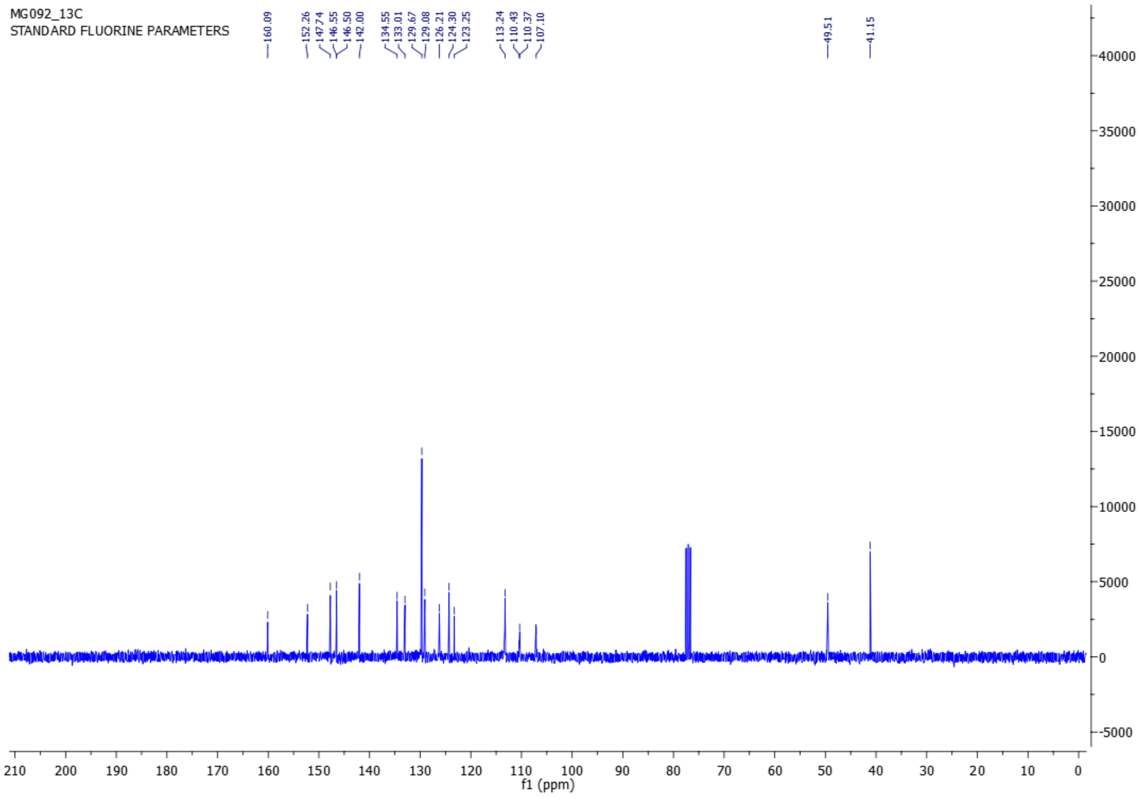

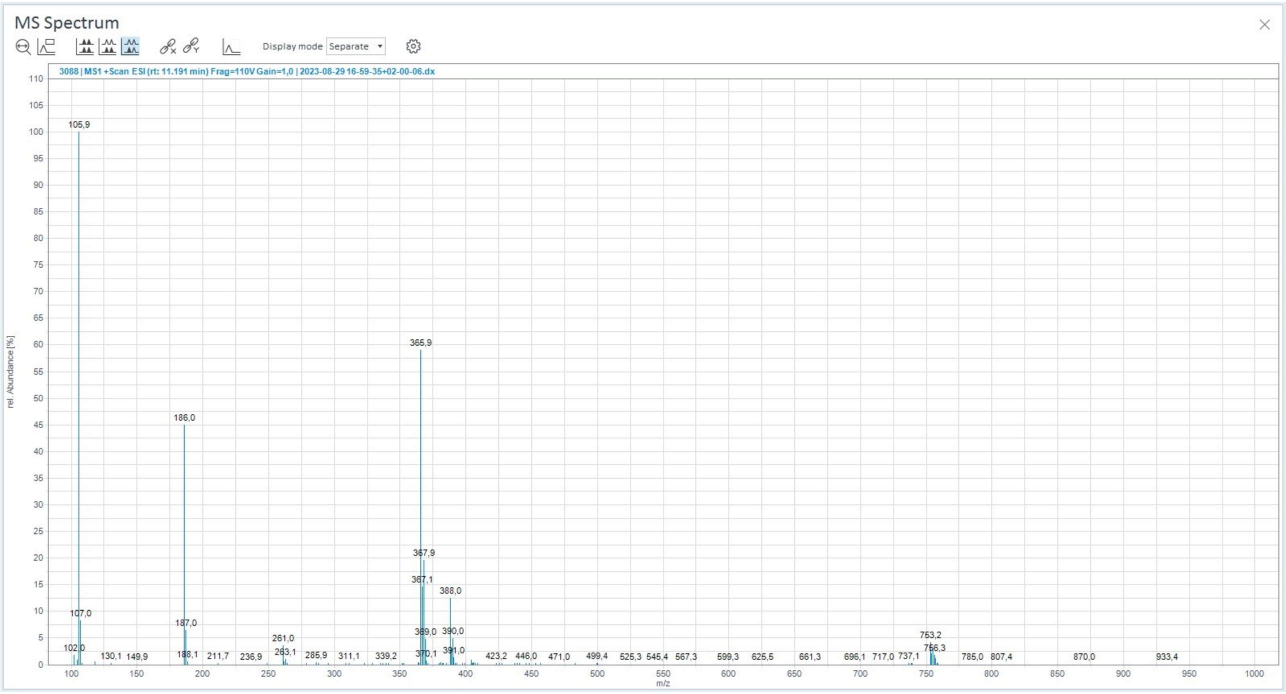

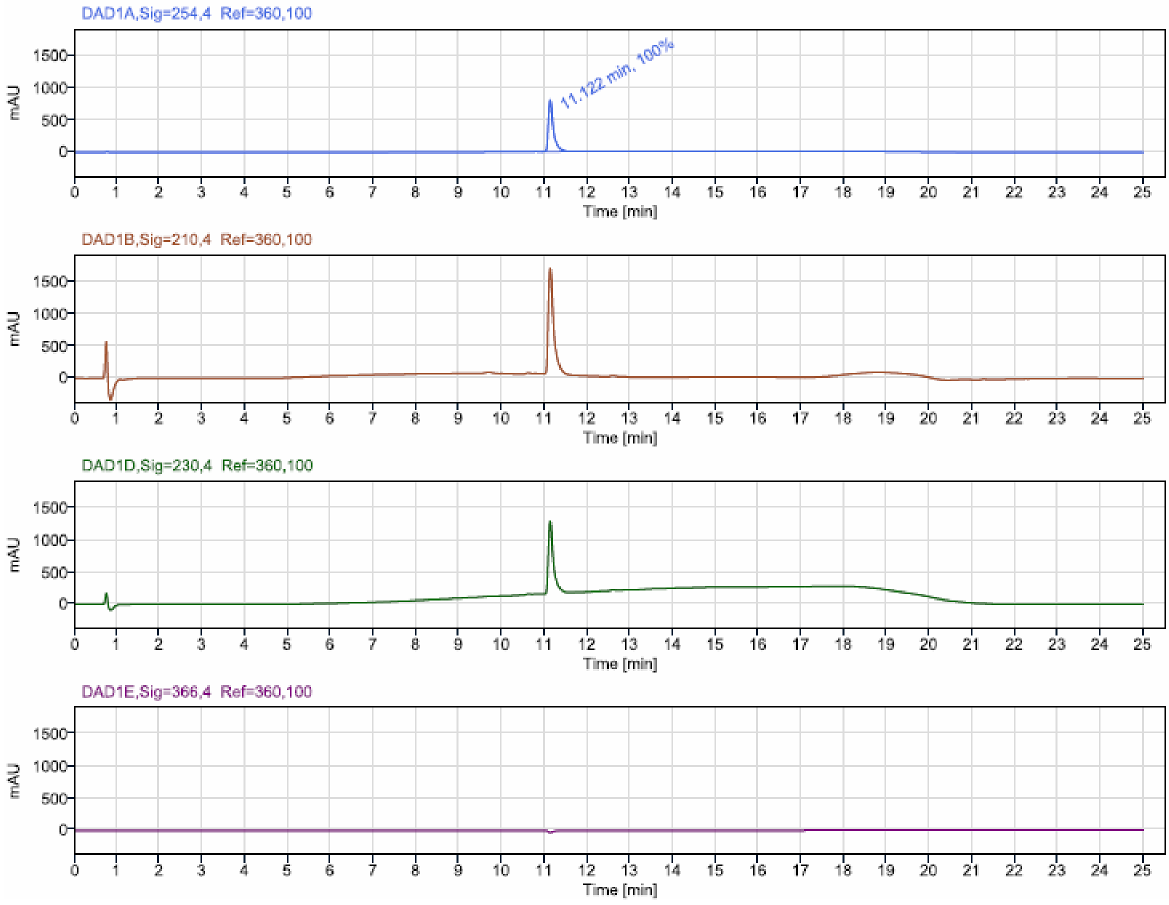

Peak Results (Area Percent at least 1%)

RT (min)	Signal Description	Width (min)	Area	Height	Area%
11.122	DAD1A,Sig=254,4 Ref=360,100	0.744	6527.2	799.6	100.00
11.194	MS1 +TIC SCAN ESI Frag=110V Gain=1,0	0.641	13906450.8	1242457.4	100.00

^1H, ^{13}C NMR and HPLC/MS spectra of compound 11b

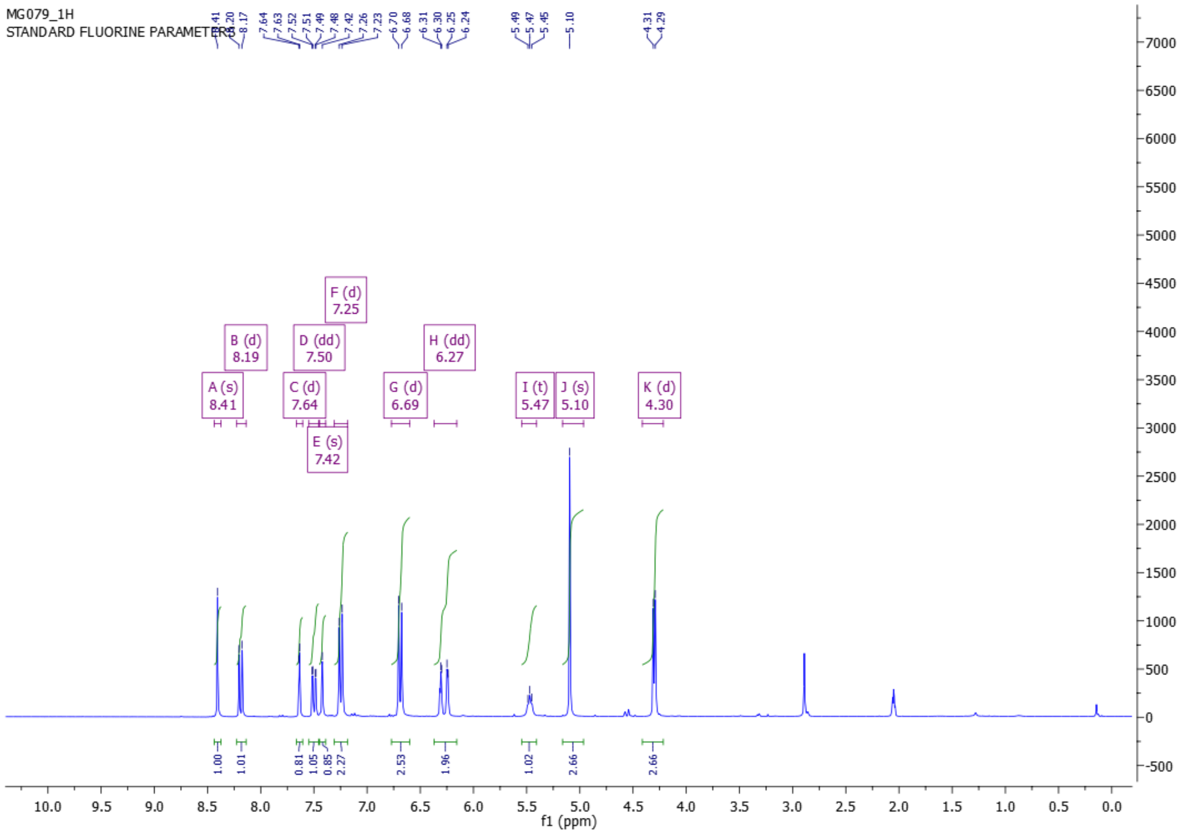

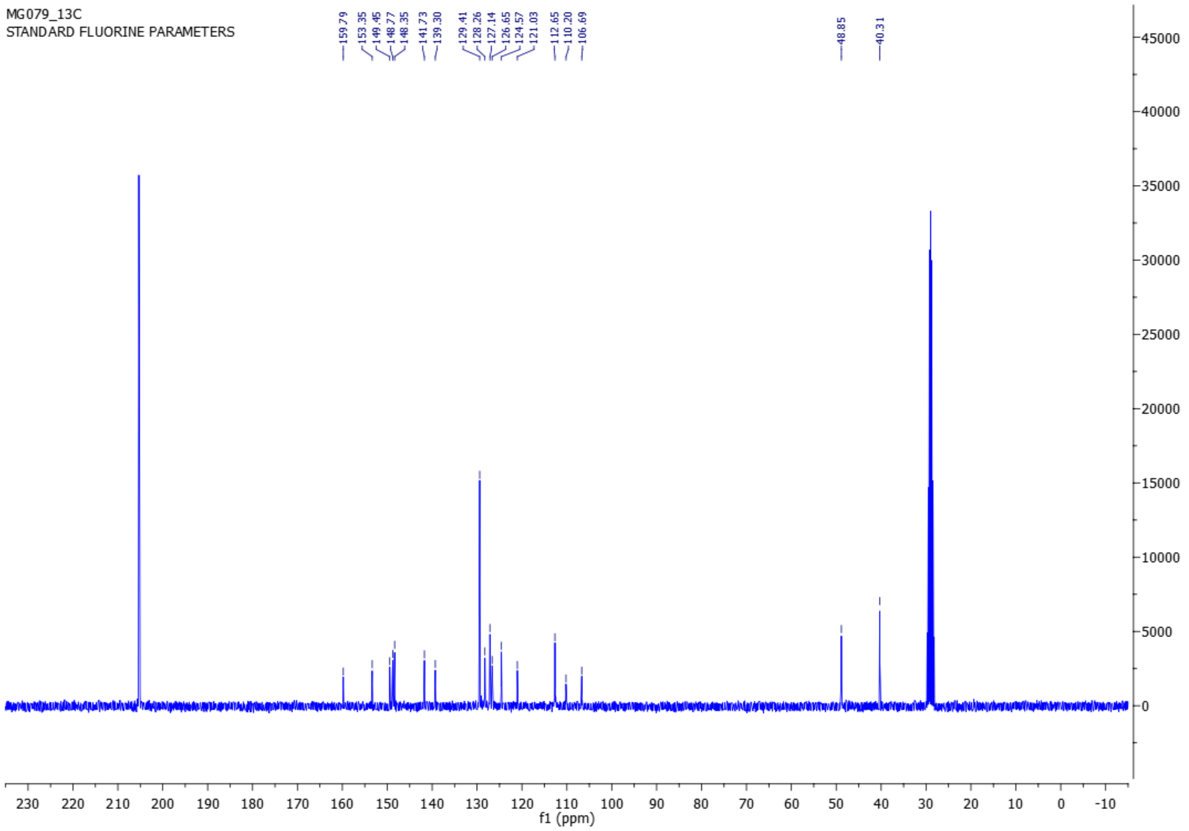

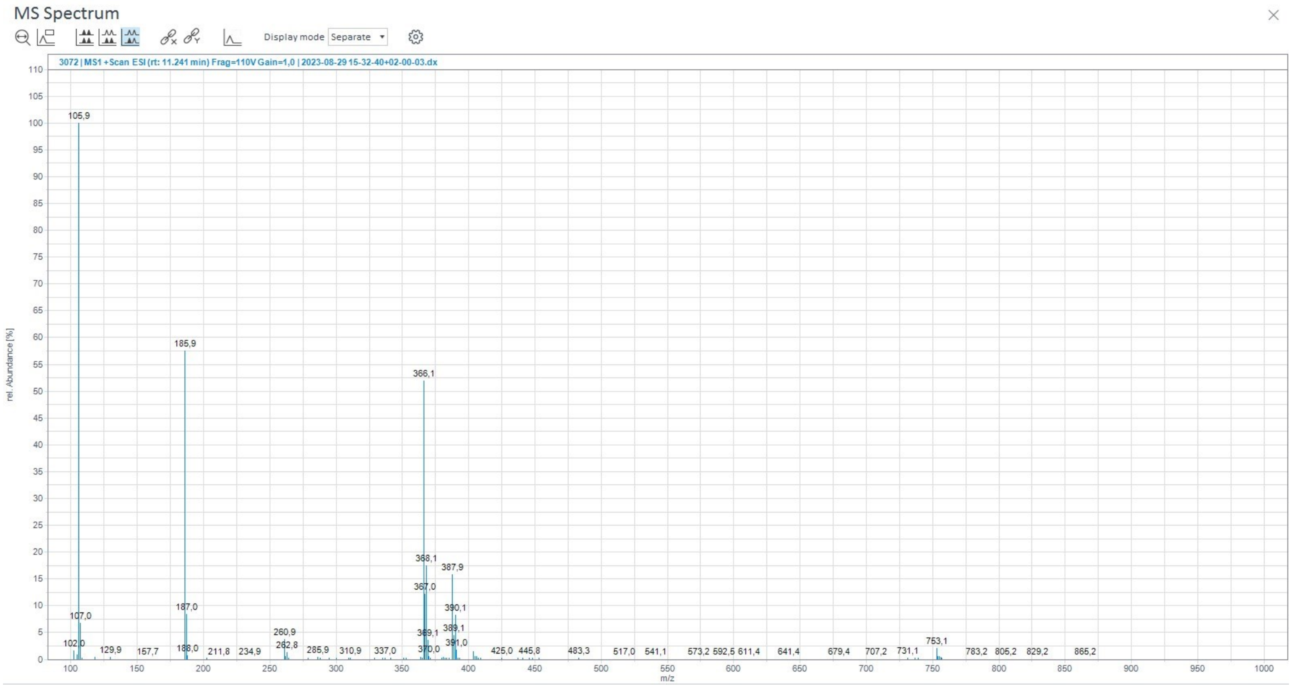

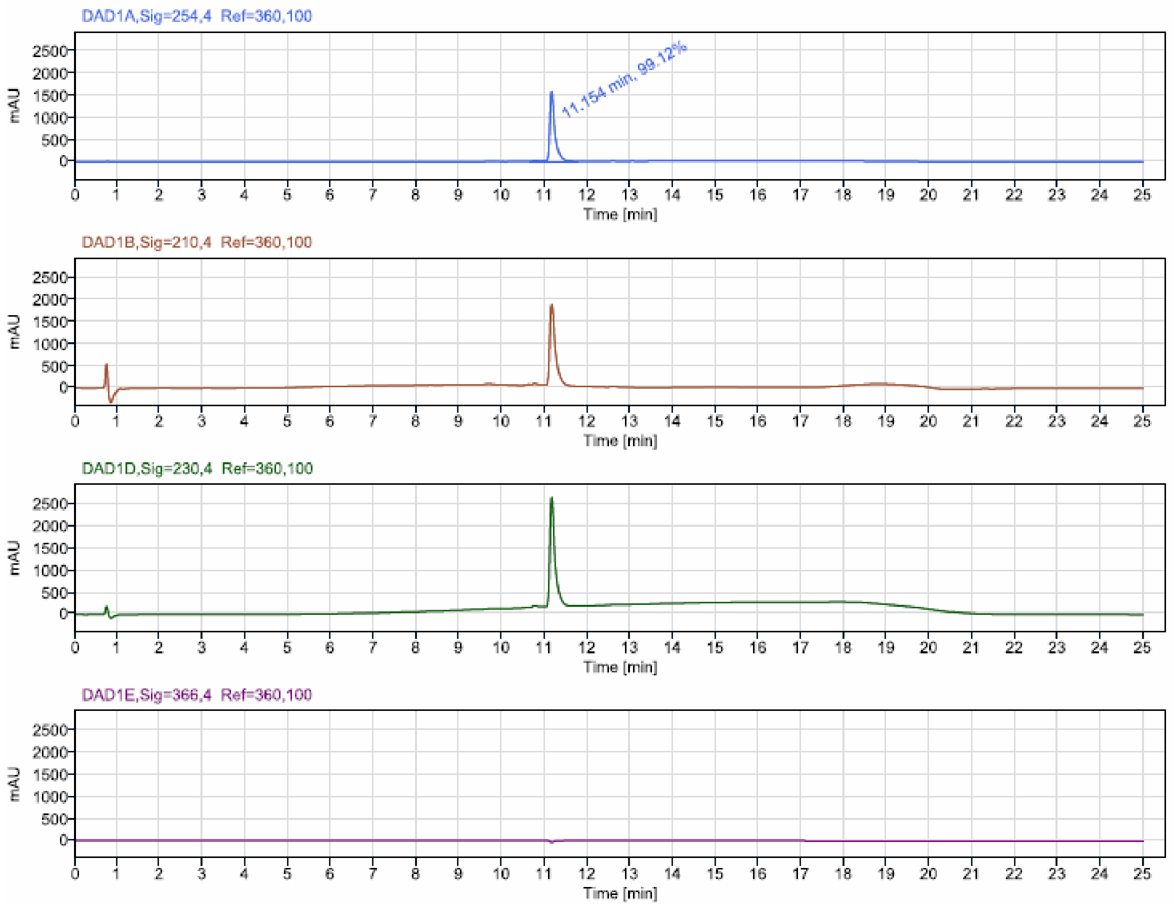

Peak Results (Area Percent at least 1%)

RT (min)	Signal Description	Width (min)	Area	Height	Area%
11.154	DAD1A,Sig=254,4 Ref=360,100	0.862	12972.6	1597.1	99.12
11.237	MS1 +TIC SCAN ESI Frag=110V Gain=1.0	0.245	7000609.1	954160.4	100.00

^{1}H, ^{13}C NMR and HPLC/MS spectra of compound 11c

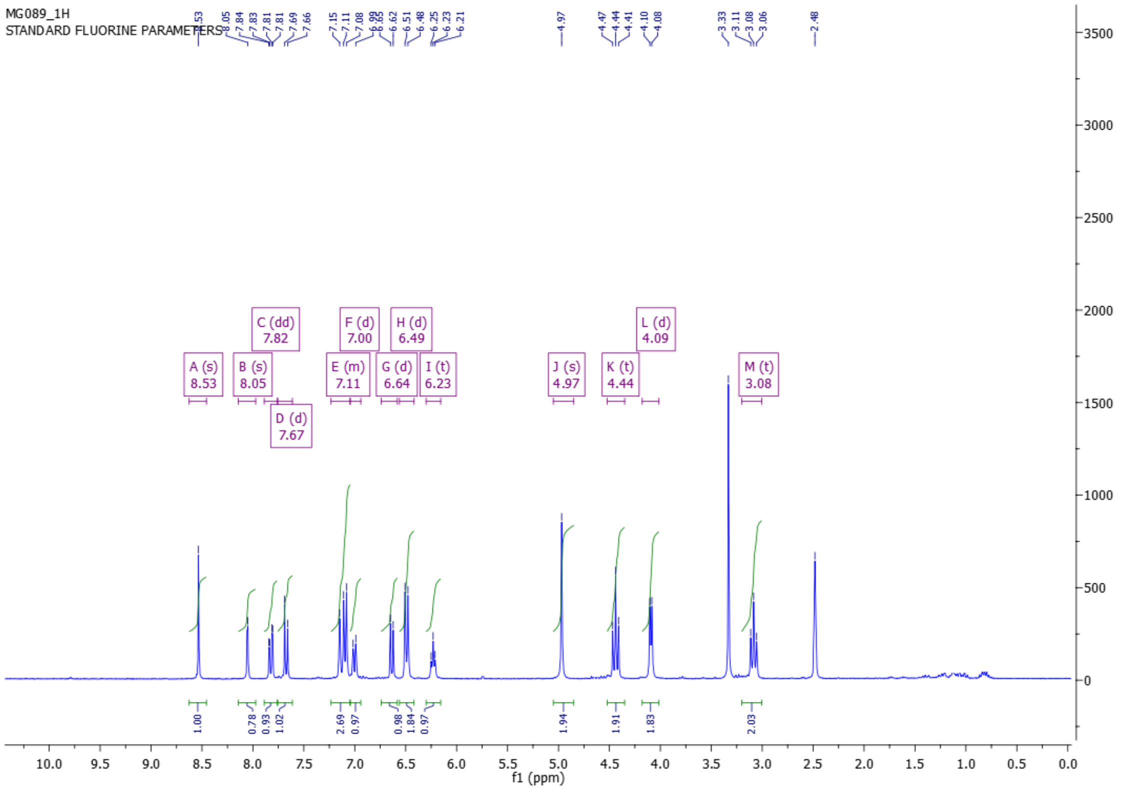

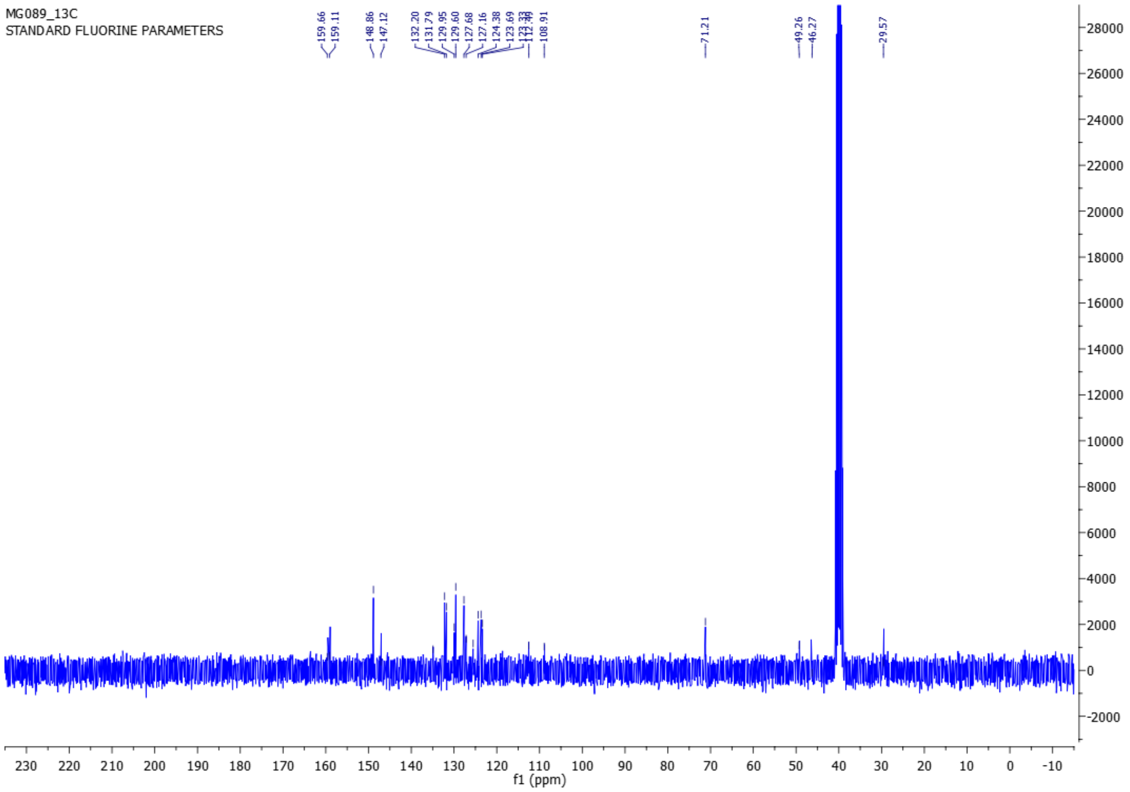

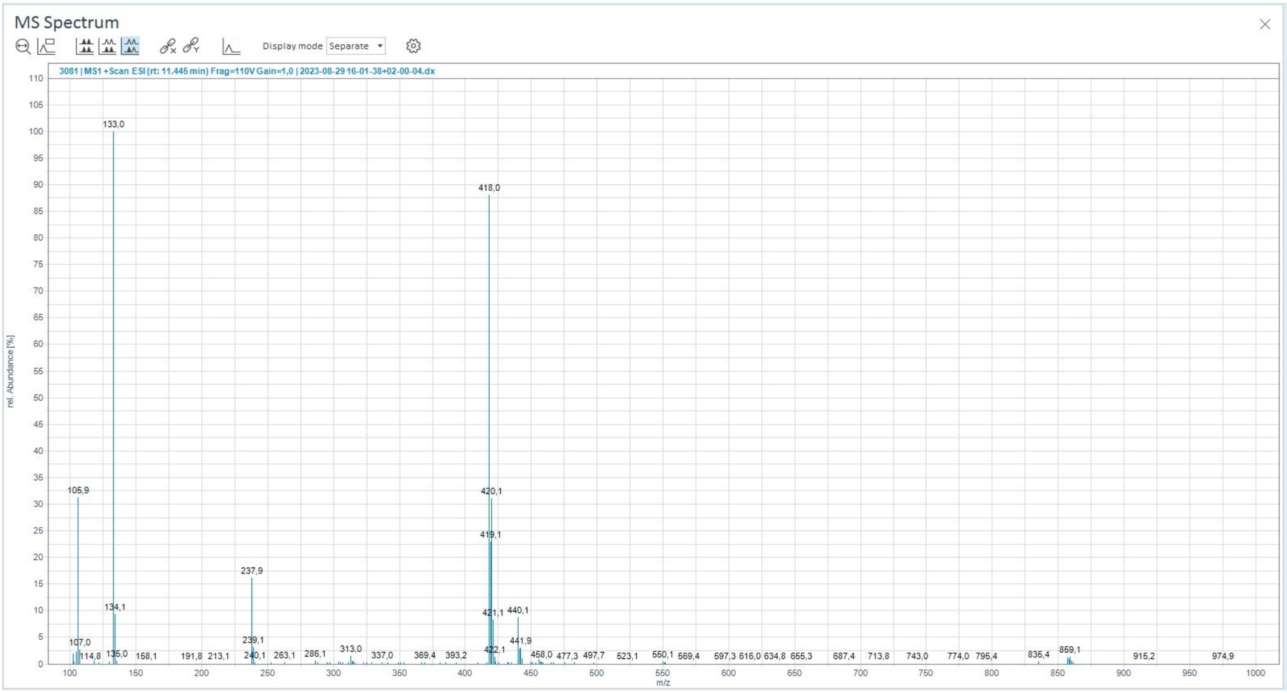

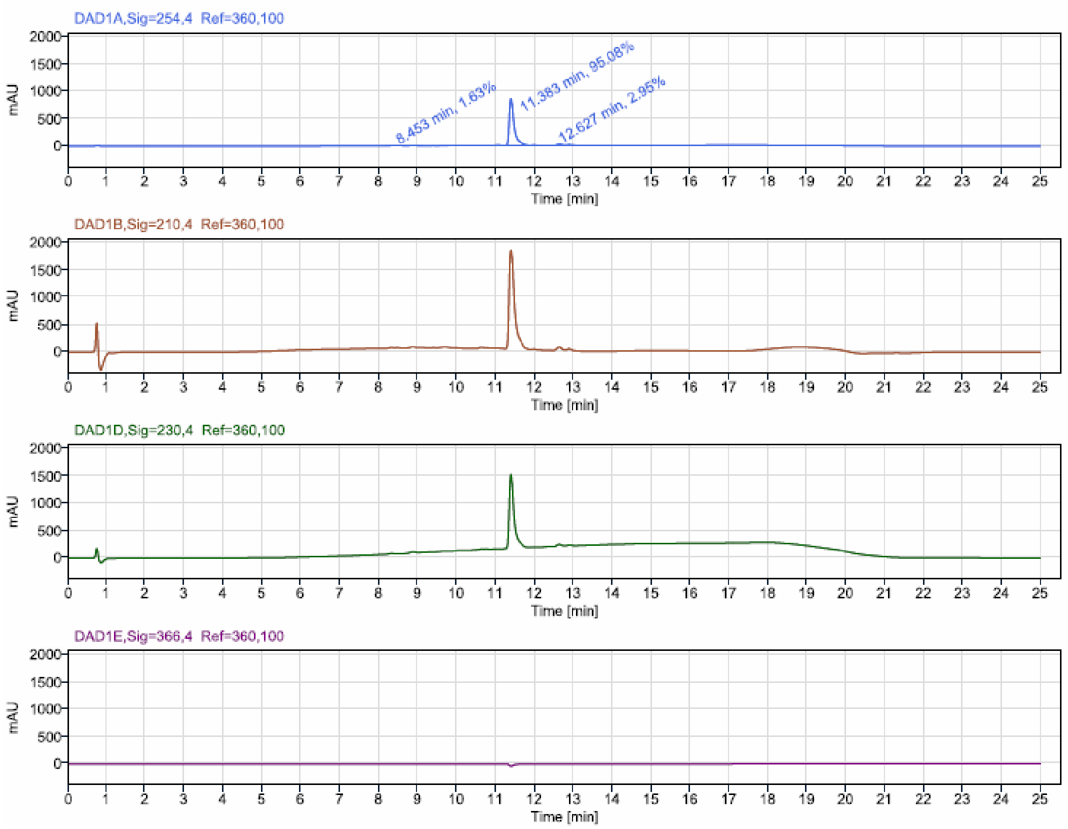

Peak Results (Area Percent at least 1%)

RT (min)	Signal Description	Width (min)	Area	Height	Area%
8.453	DAD1A,Sig=254,4 Ref=360,100	0.937	122.9	6.7	1.63
11.383	DAD1A,Sig=254,4 Ref=360,100	0.829	7149.8	855.5	95.08
11,450	MS1 +TIC SCAN ESI Frag=110V Gain=1,0	0,666	18189544,3	1504397,8	100,00
12.627	DAD1A,Sig=254,4 Ref=360,100	0.757	221.9	18.7	2.95

^{1}H, ^{13}C NMR and HPLC/MS spectra of compound 11d

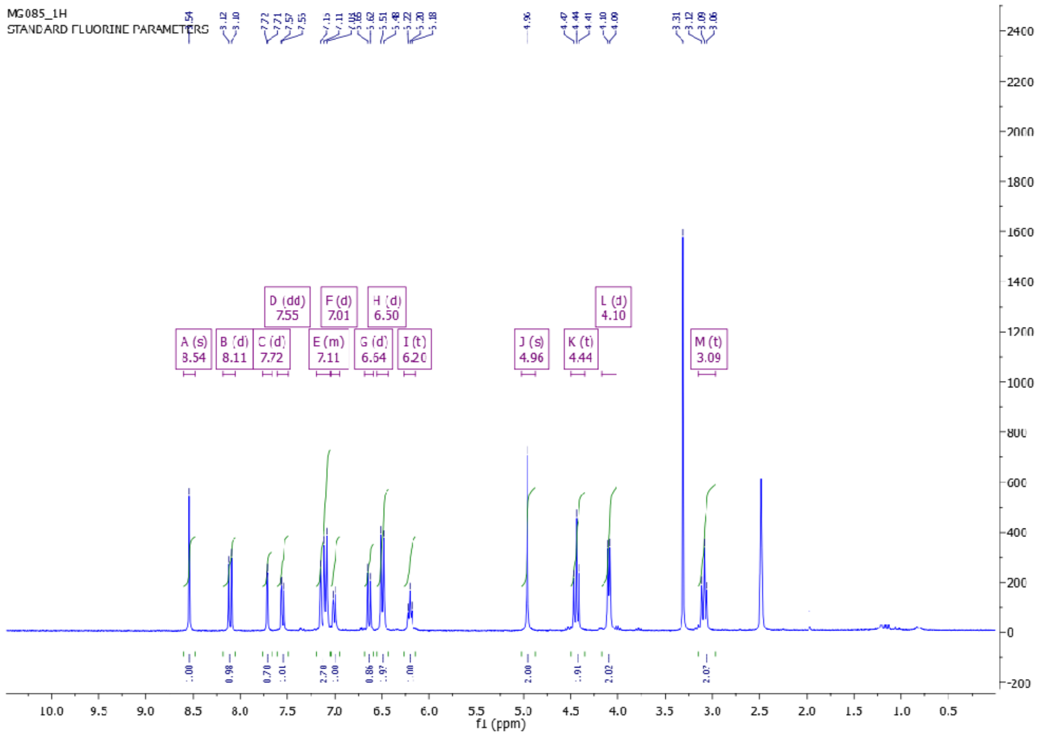

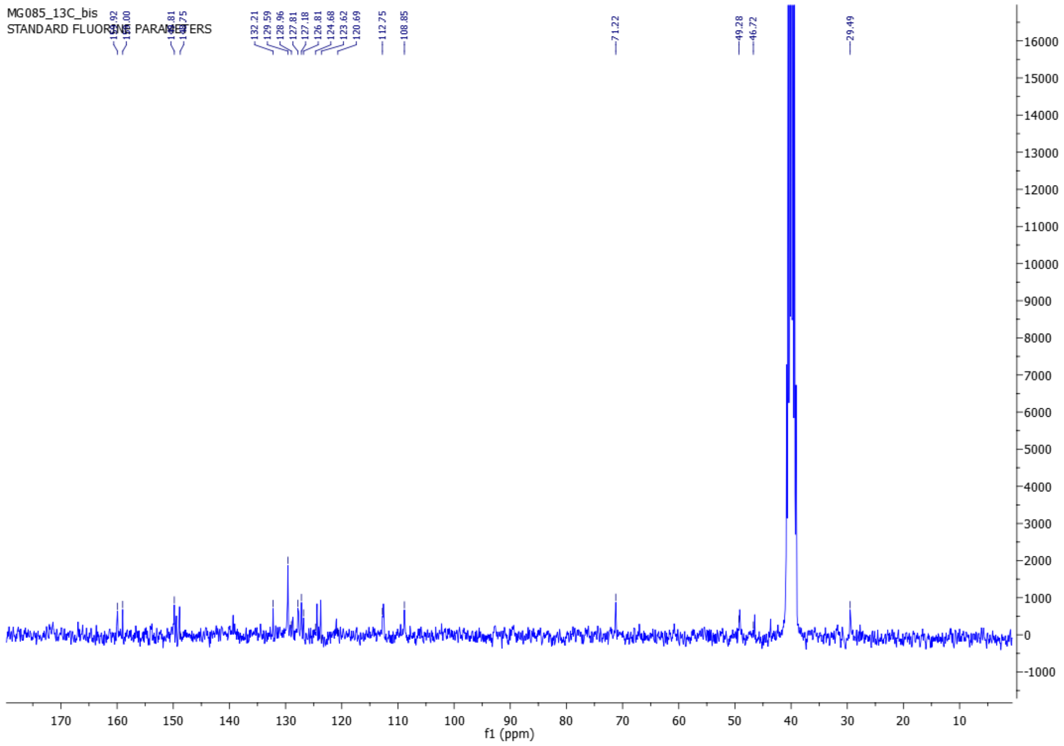

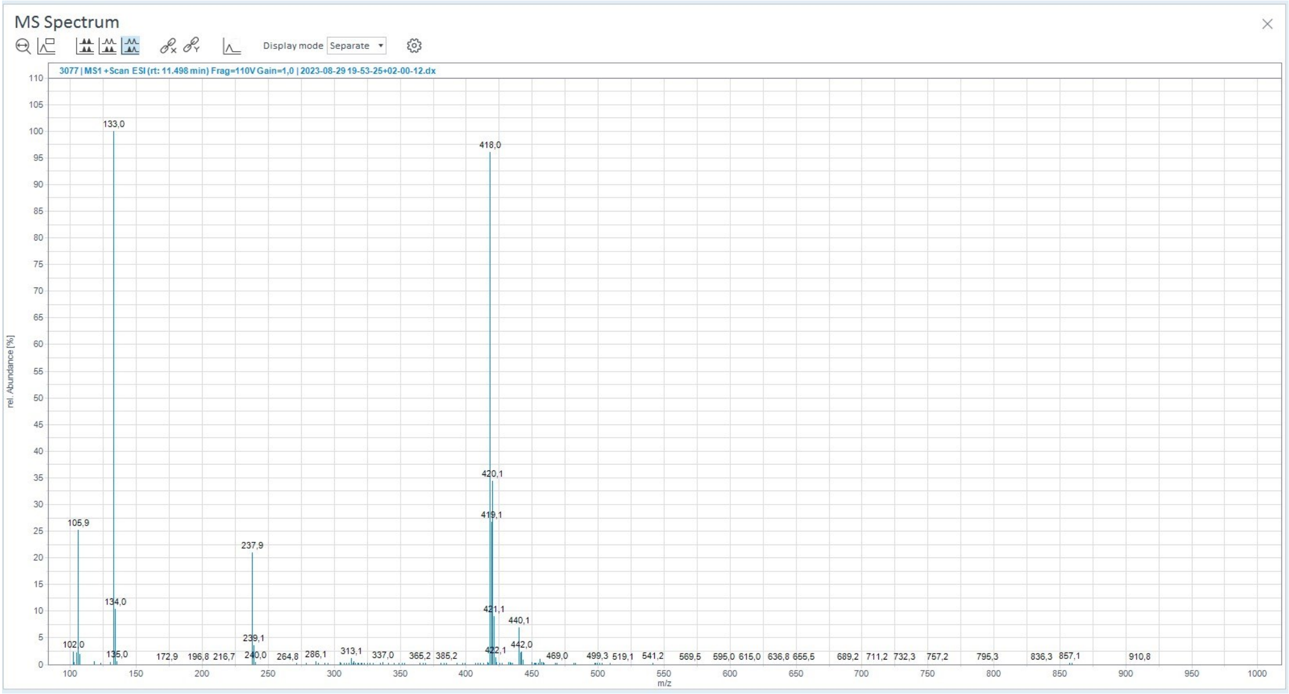

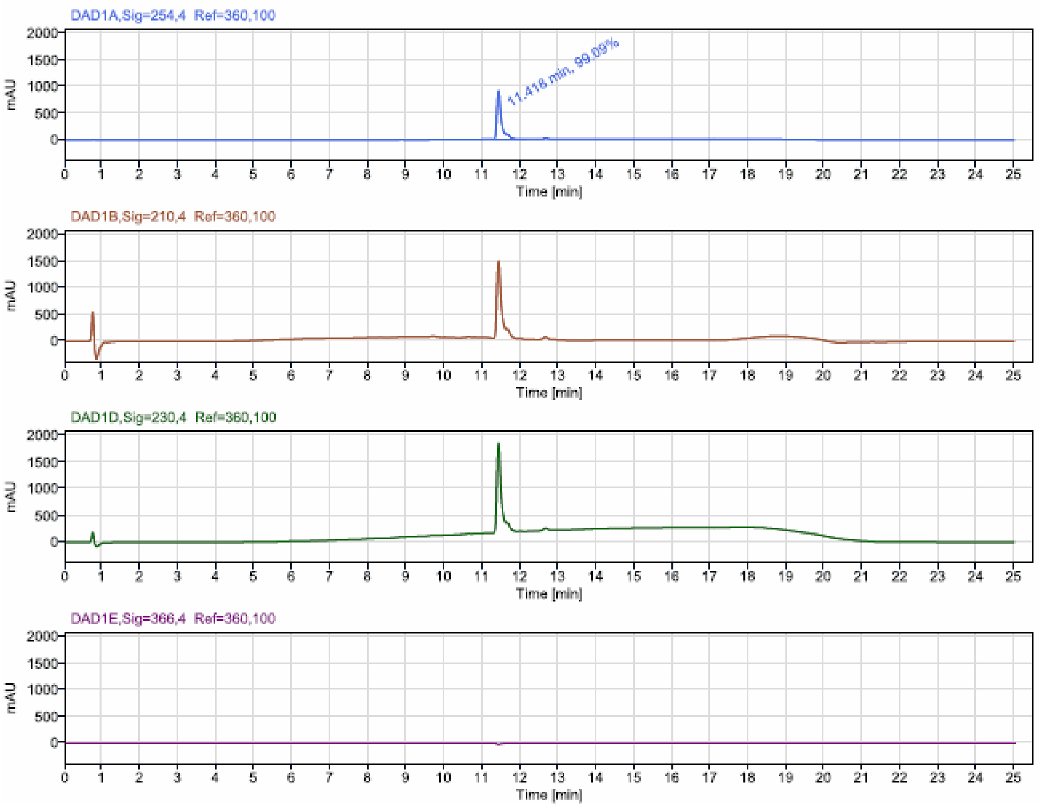

Peak Results (Area Percent at least 1%)

RT (min)	Signal Description	Width (min)	Area	Height	Area%
11,418	DAD1A,Sig=254,4 Ref=360,100	0,916	7954,0	921,1	99,09
11,494	MS1 +TIC SCAN ESI Frag=110V Gain=1,0	0,334	14399325,7	1429202,5	100,00

^{1}H, ^{13}C NMR and HPLC/MS spectra of compound 11e

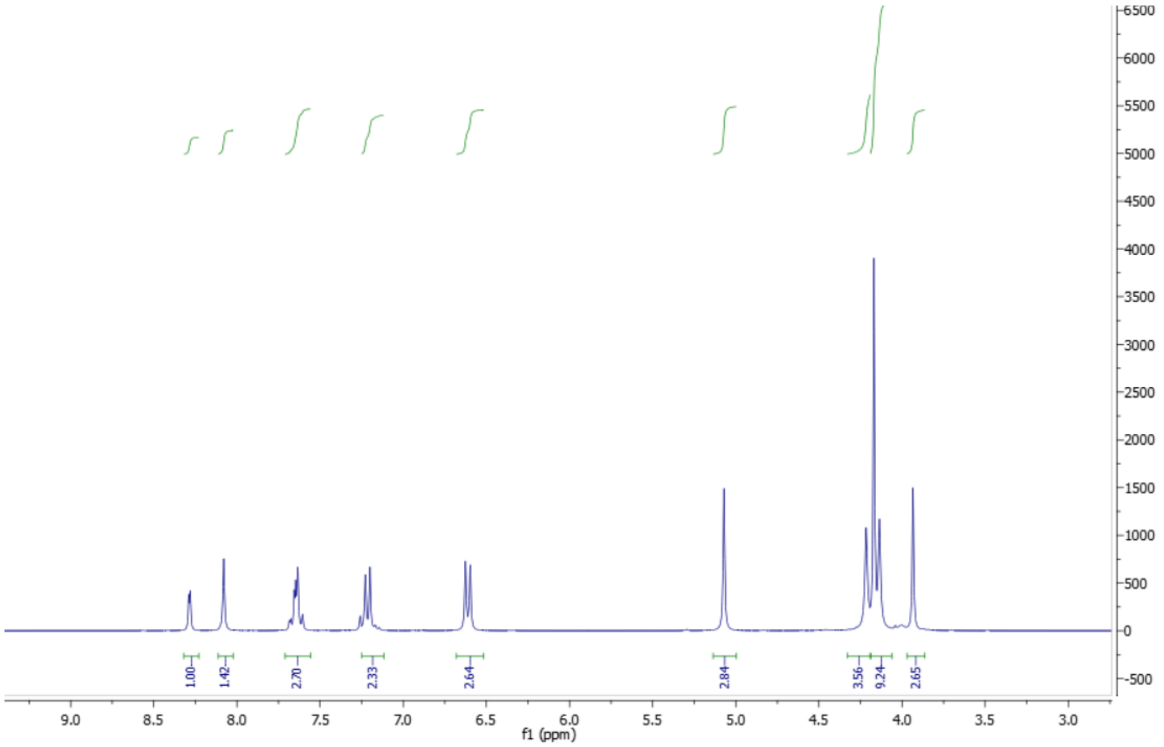

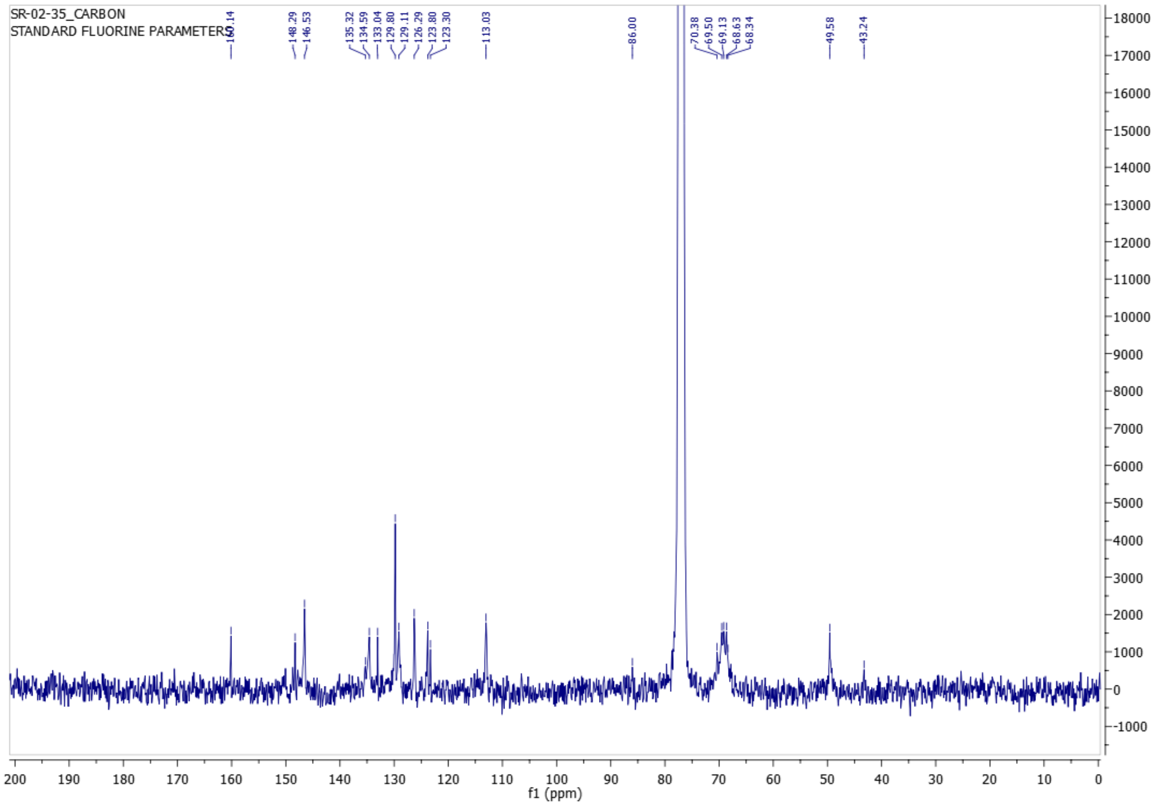

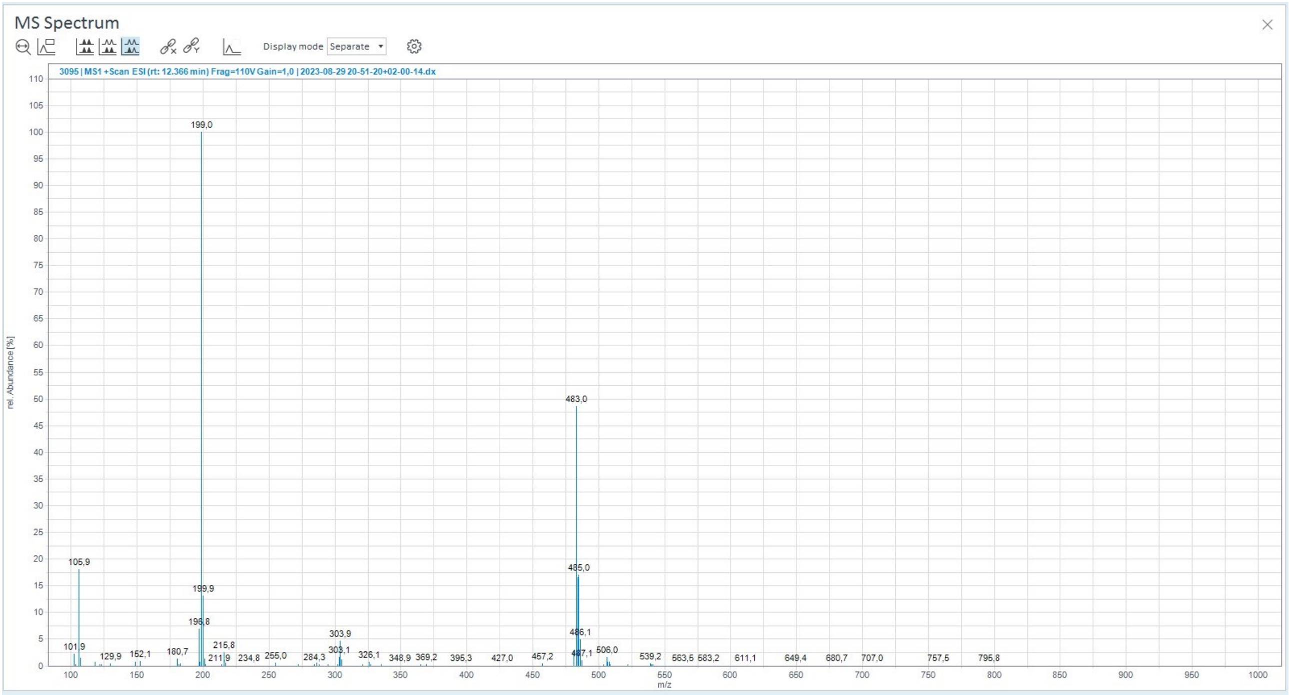

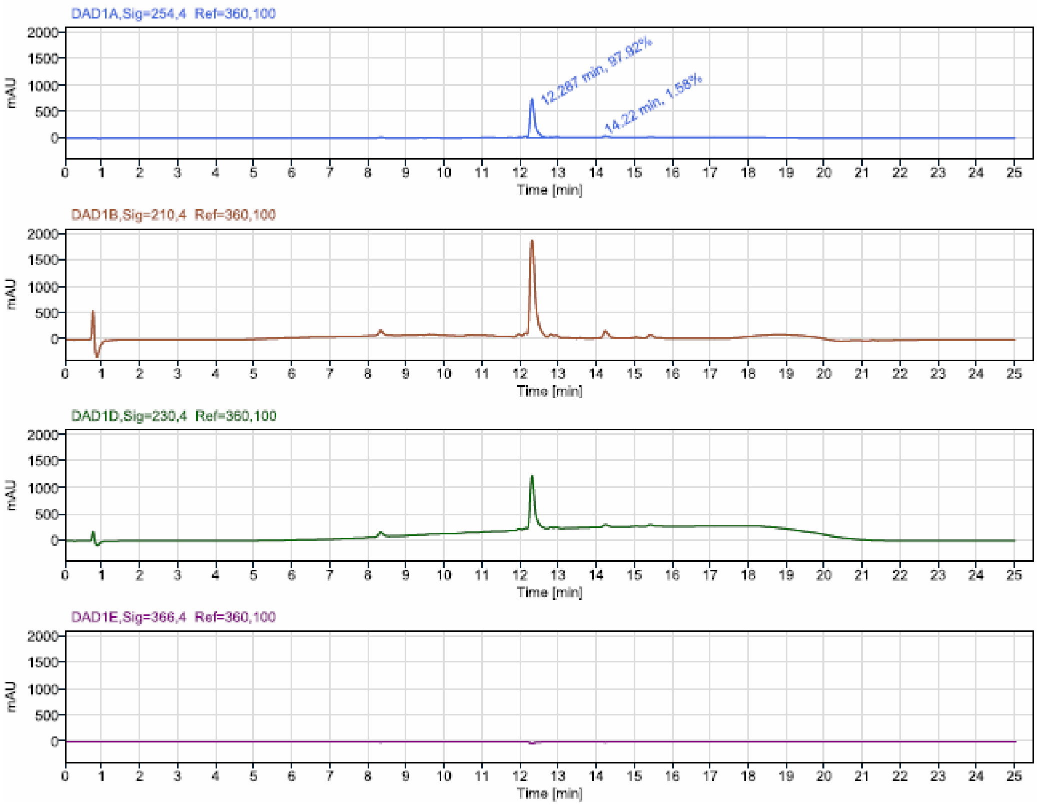

Peak Results (Area Percent at least 1%)

RT (min)	Signal Description	Width (min)	Area	Height	Area%
12,287	DAD1A,Sig=254,4 Ref=360,100	0,524	6154,9	729,0	97,92
12,366	MS1 +TIC SCAN ESI Frag=110V Gain=1,0	0,184	6862495,4	896087,9	100,00
14,220	DAD1A,Sig=254,4 Ref=360,100	0,158	99,4	17,2	1,58

^{1}H, ^{13}C NMR and HPLC/MS spectra of compound 11f

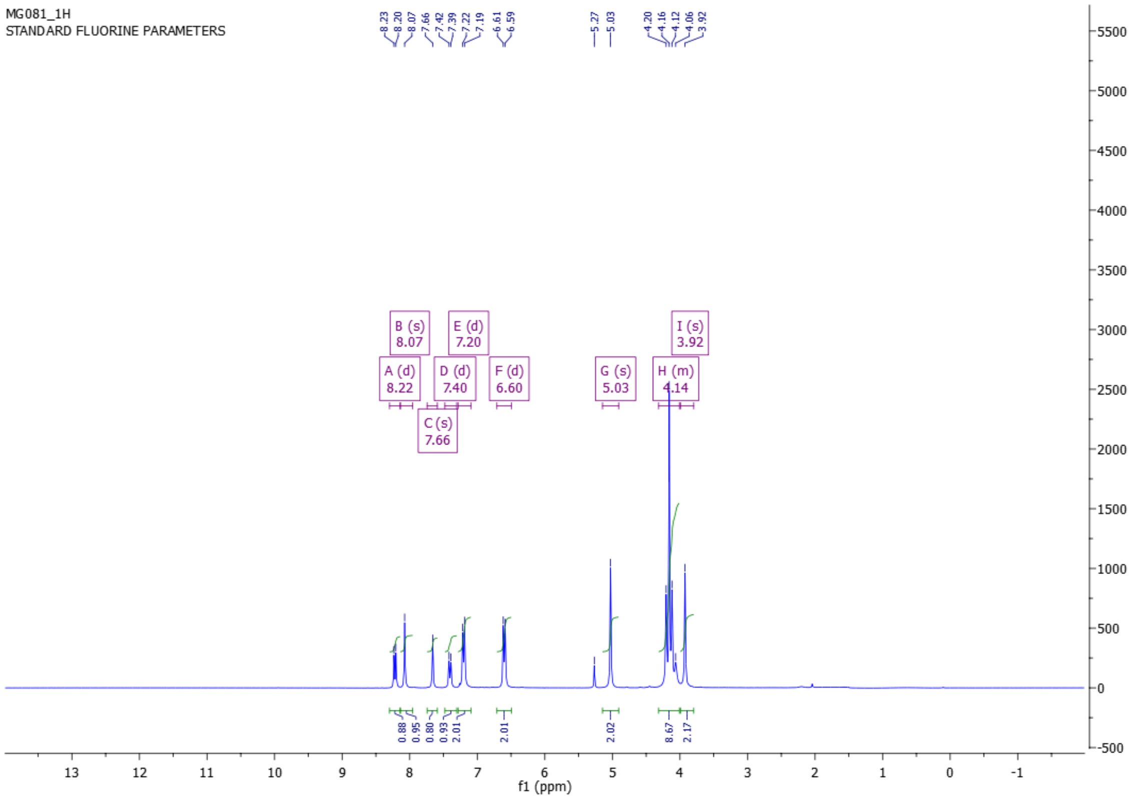

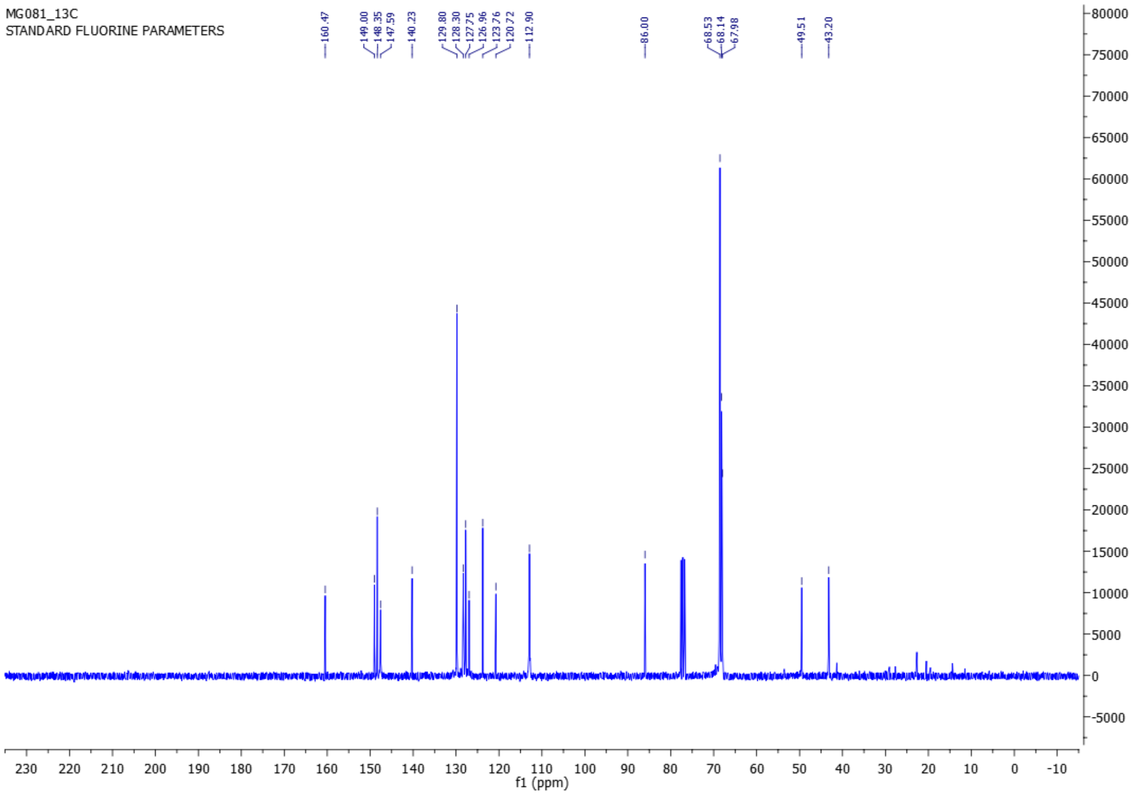

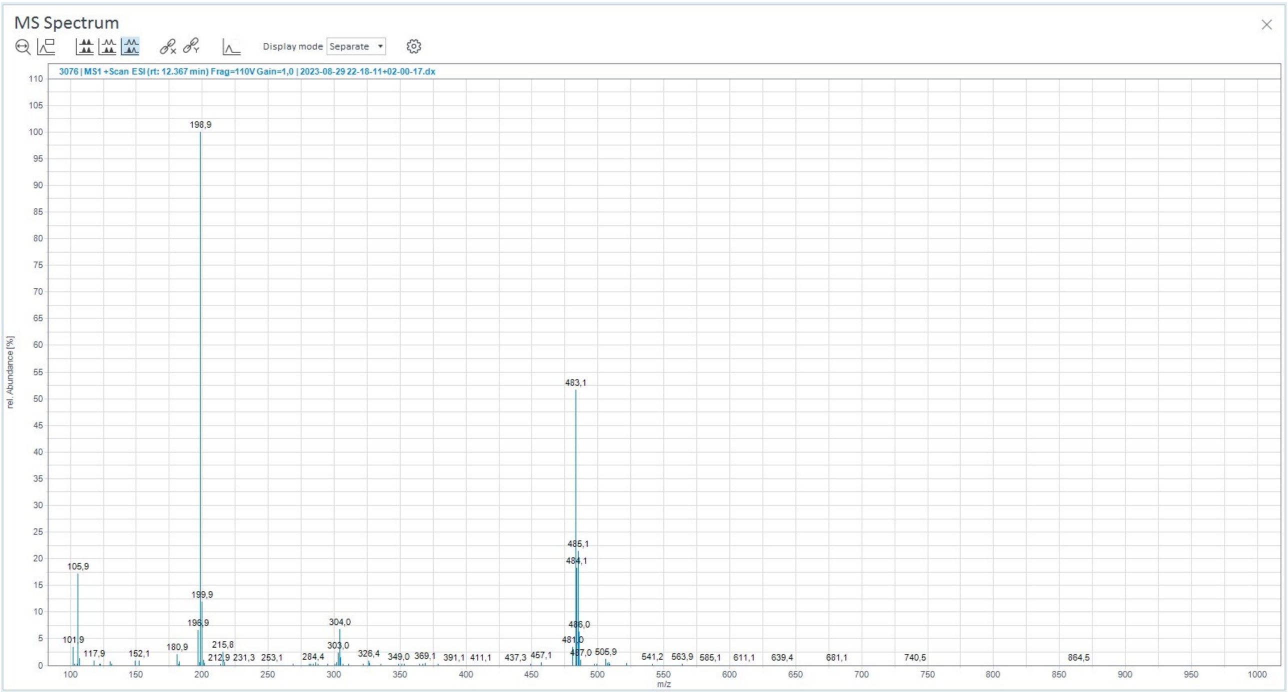

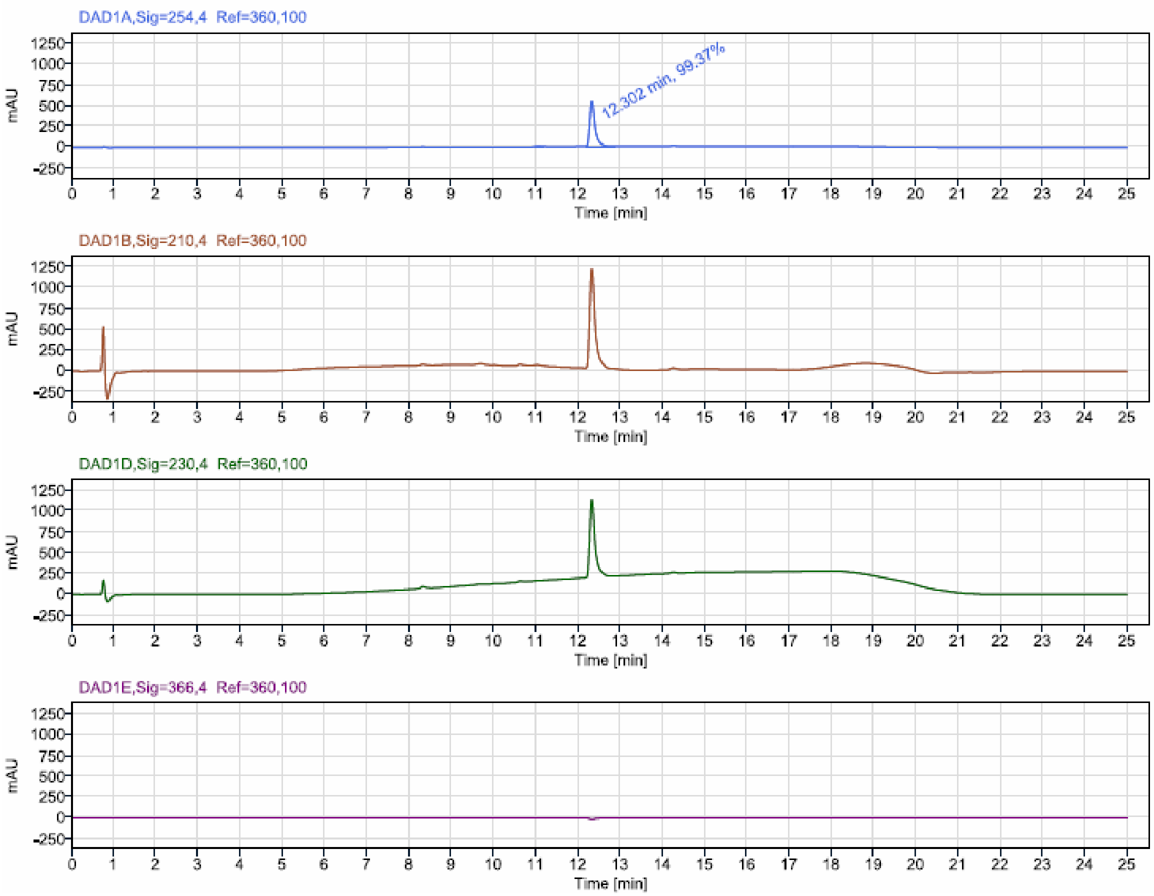

Peak Results (Area Percent at least 1%)

RT (min)	Signal Description	Width (min)	Area	Height	Area%
12.302	DAD1A,Sig=254,4 Ref=360,100	0.669	4656.8	541.3	99.37
12.364	MS1 +TIC SCAN ESI Frag=110V Gain=1.0	0.106	1641912.5	363973.6	100.00

^{1}H, ^{13}C NMR and HPLC/MS spectra of compound 22a

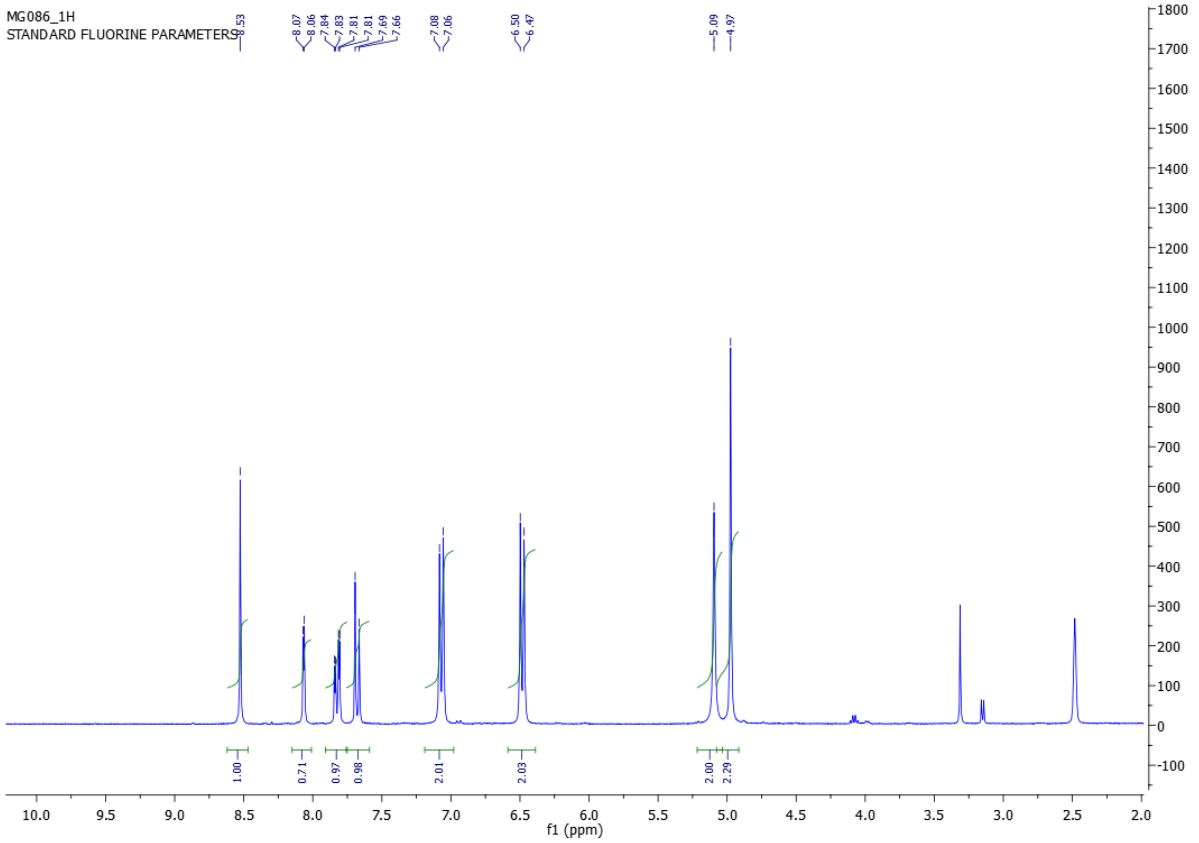

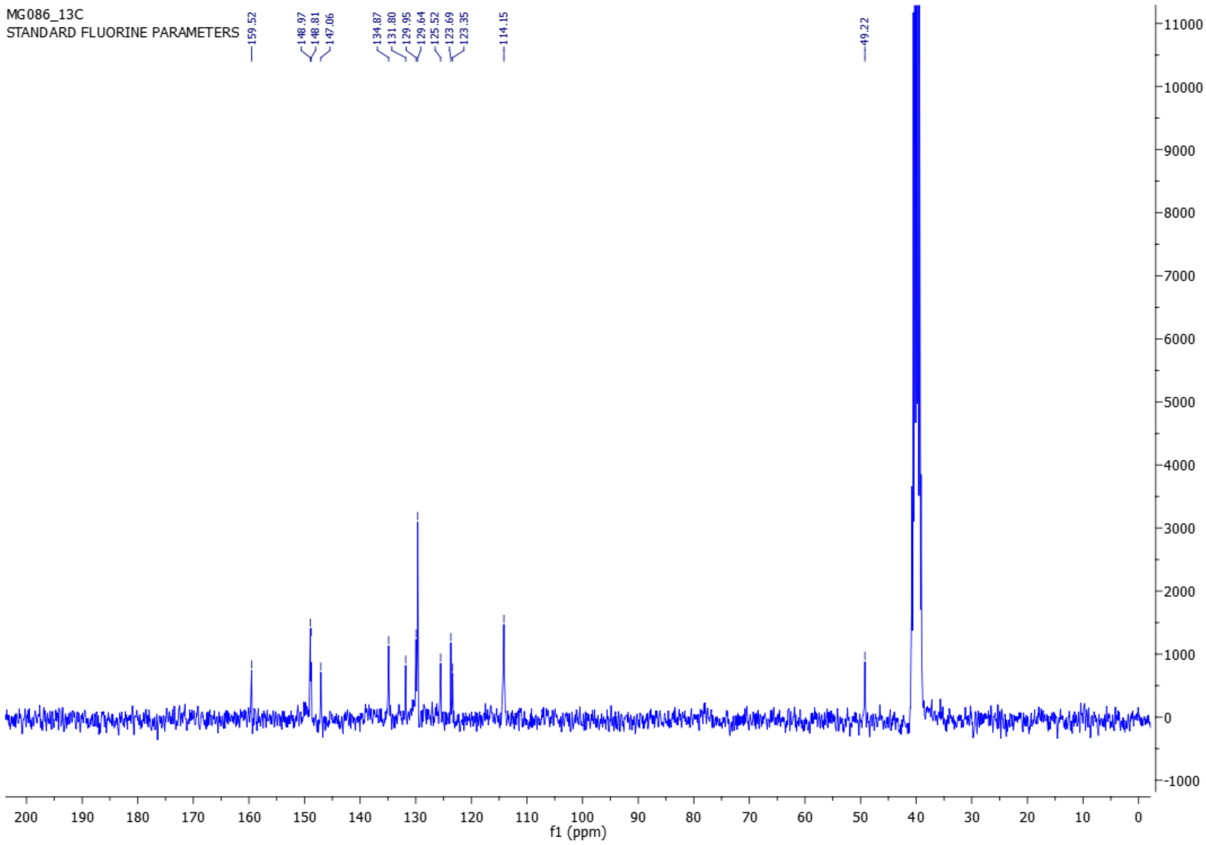

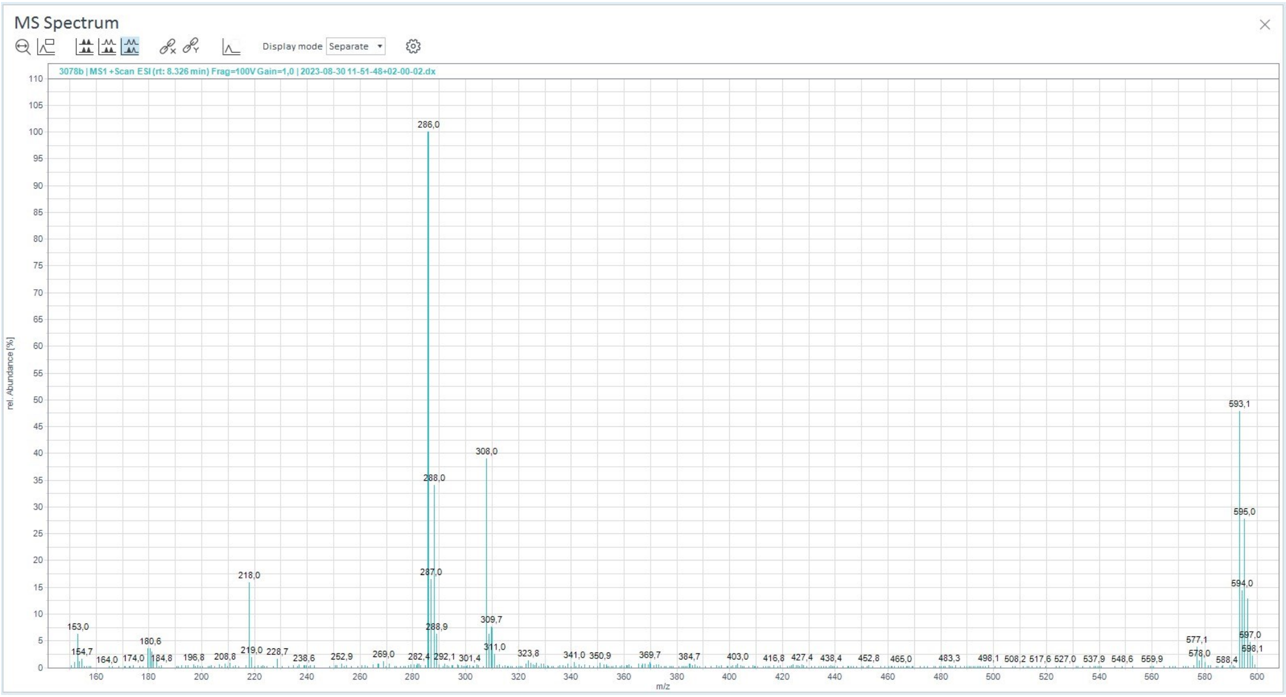

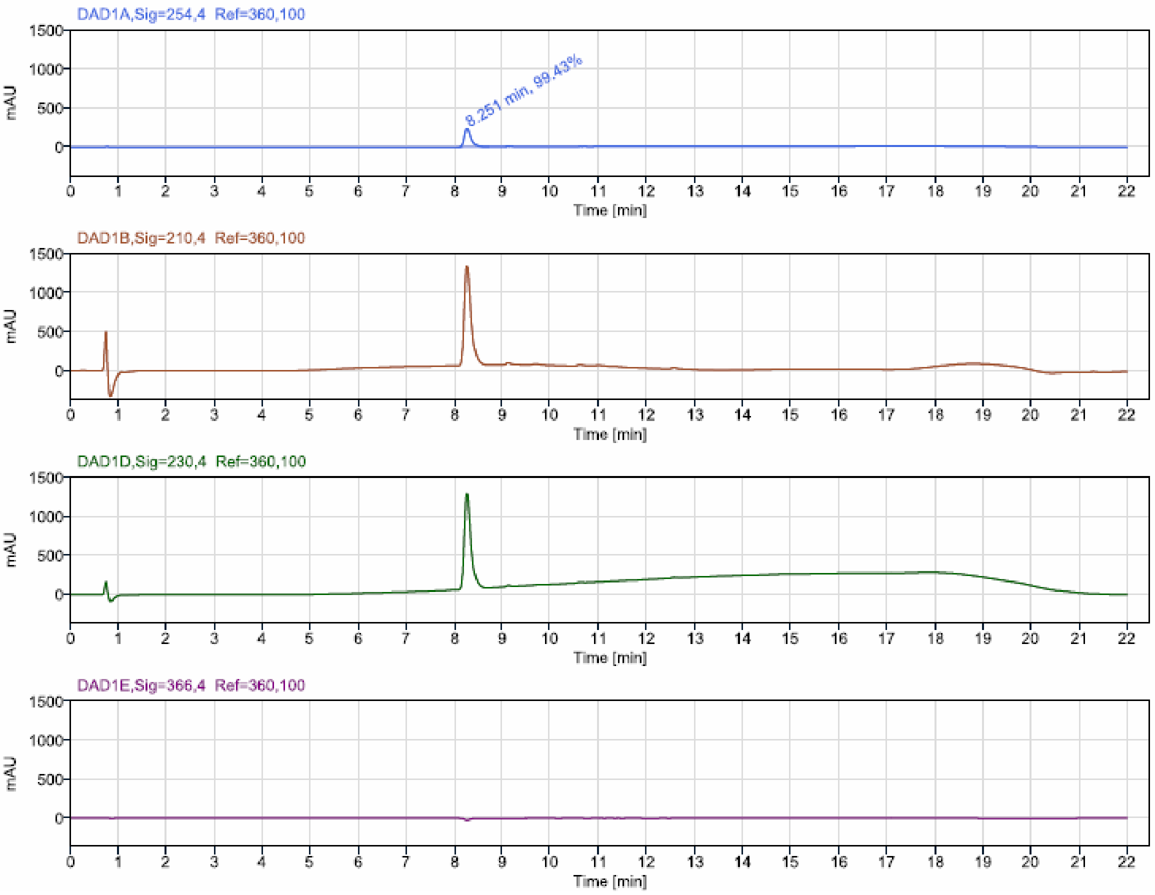

Peak Results (Area Percent at least 1%)

RT (min)	Signal Description	Width (min)	Area	Height	Area%
8.251	DAD1A,Sig=254,4 Ref=360,100	0.663	2282.5	239.0	99.43
8.324	MS1 +TIC SCAN ESI Frag=100V Gain=1.0	0.418	3273900.3	329227.4	100.00
8.326	MS1 +TIC SIM ESI Frag=100V Gain=1.0	0.461	948102.5	83126.0	100.00
8.361	MS1 -TIC SCAN ESI Frag=100V Gain=1.0	0.676	268240.5	18259.0	100.00

^{1}H, ^{13}C NMR and HPLC/MS spectra of compound 22b

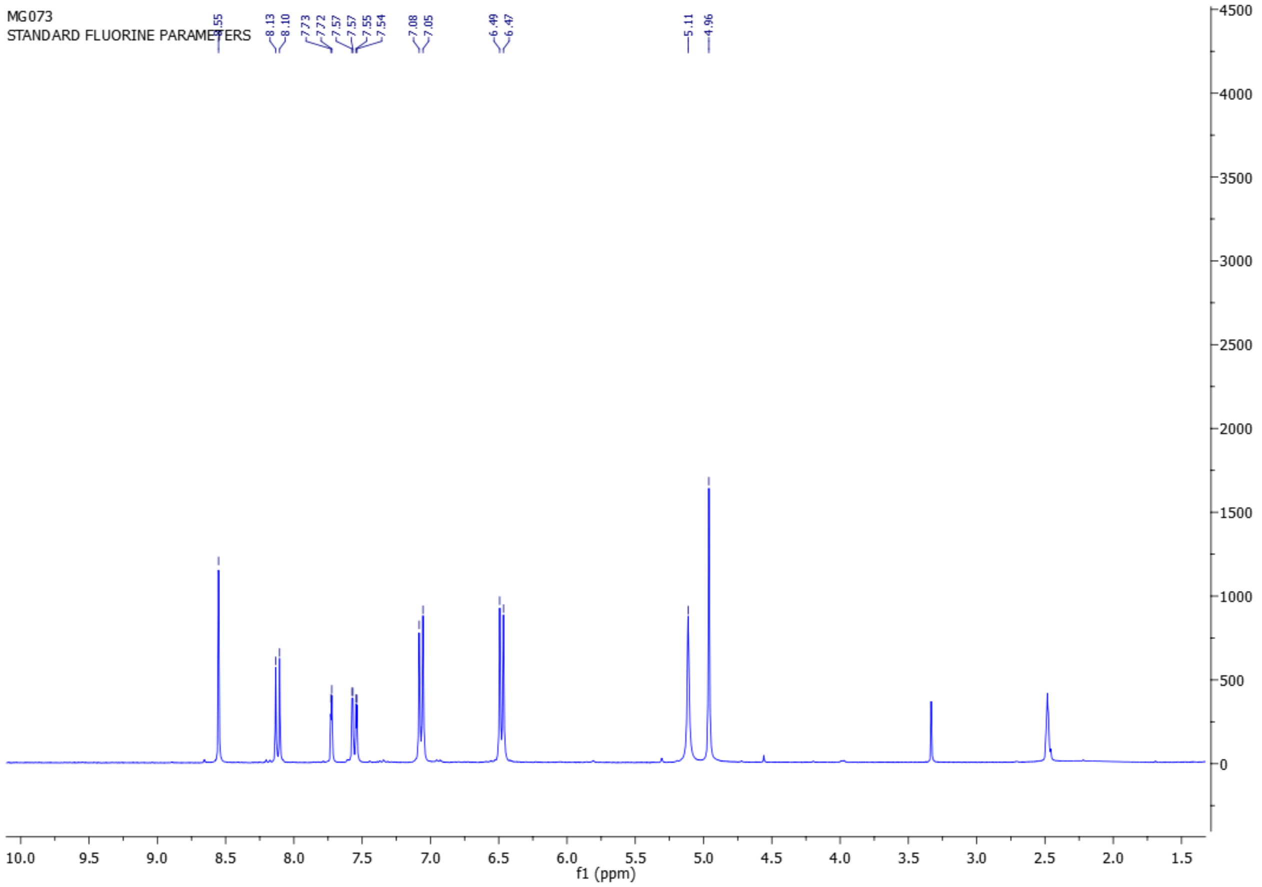

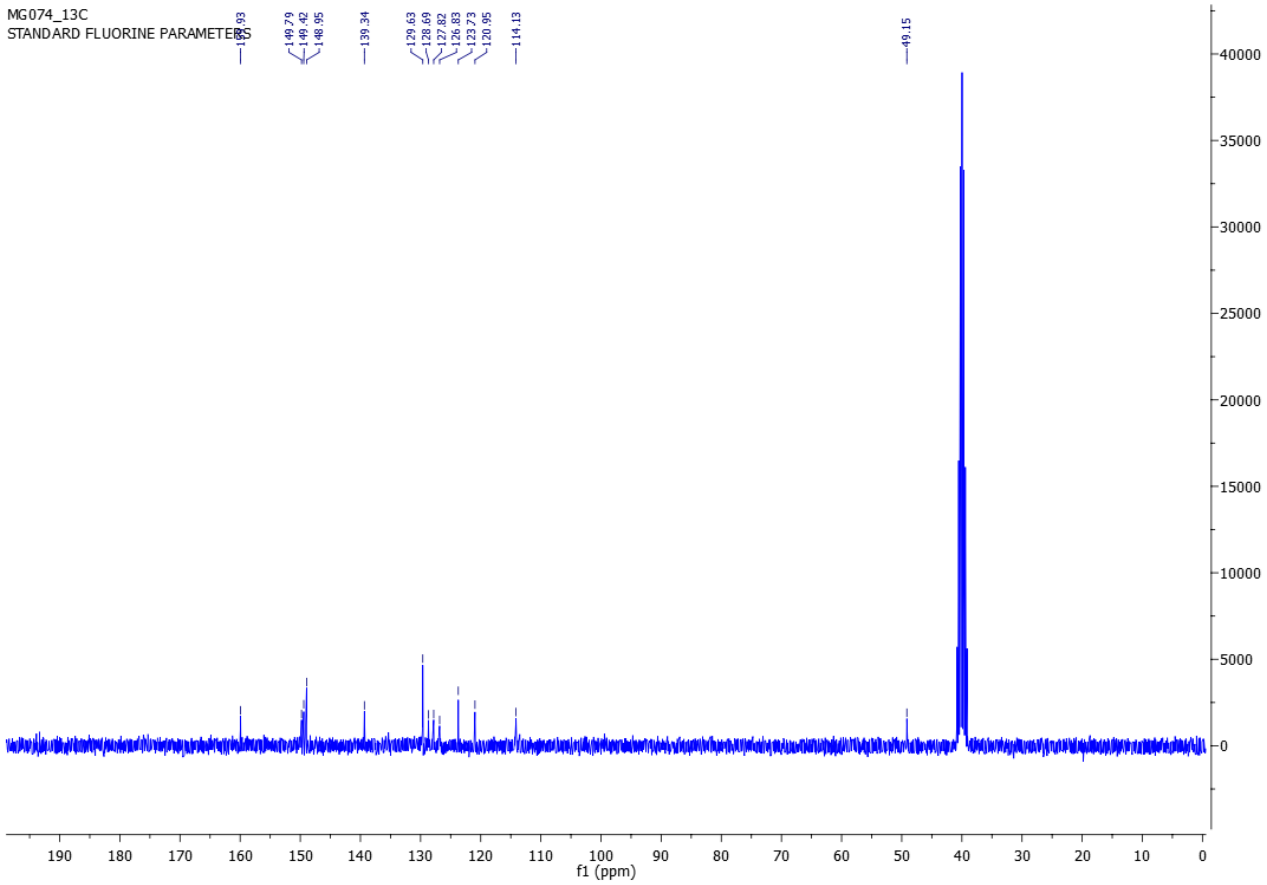

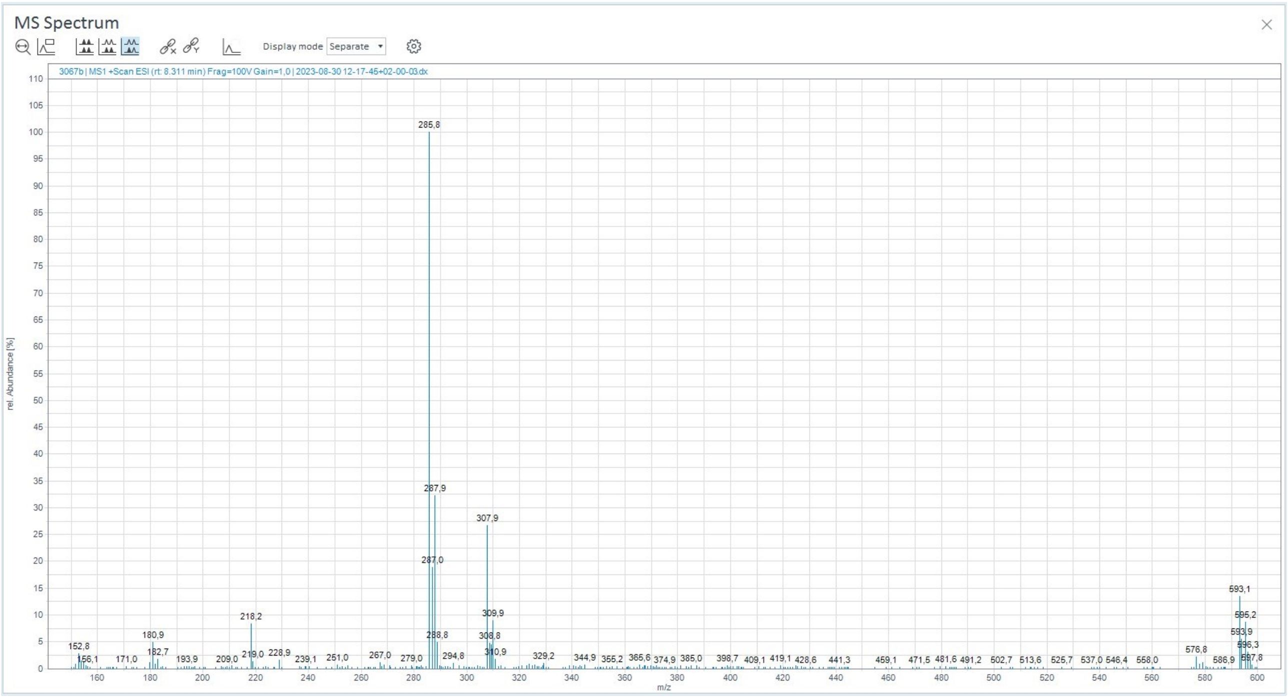

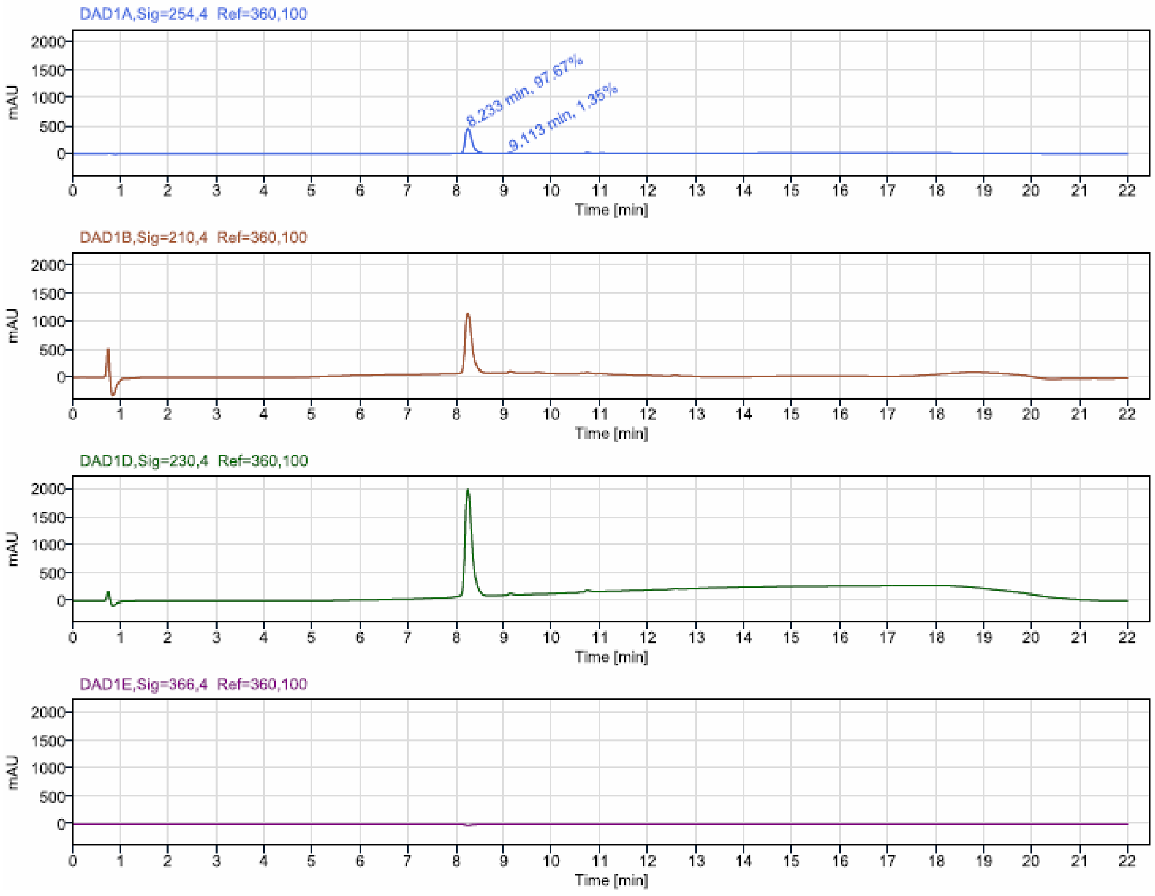

Peak Results (Area Percent at least 1%)

RT (min)	Signal Description	Width (min)	Area	Height	Area%
8.233	DAD1A,Sig=254,4 Ref=360,100	0.740	4456.3	450.0	97.67
8,304	MS1 +TIC SIM ESI Frag=100V Gain=1,0	1,183	1243736,5	101700,5	100,00
8,309	MS1 +TIC SCAN ESI Frag=100V Gain=1,0	0,718	3806960,1	301412,0	100,00
9.113	DAD1A,Sig=254,4 Ref=360,100	0.142	61.7	13.0	1.35

HRMS spectrum of compound 7a

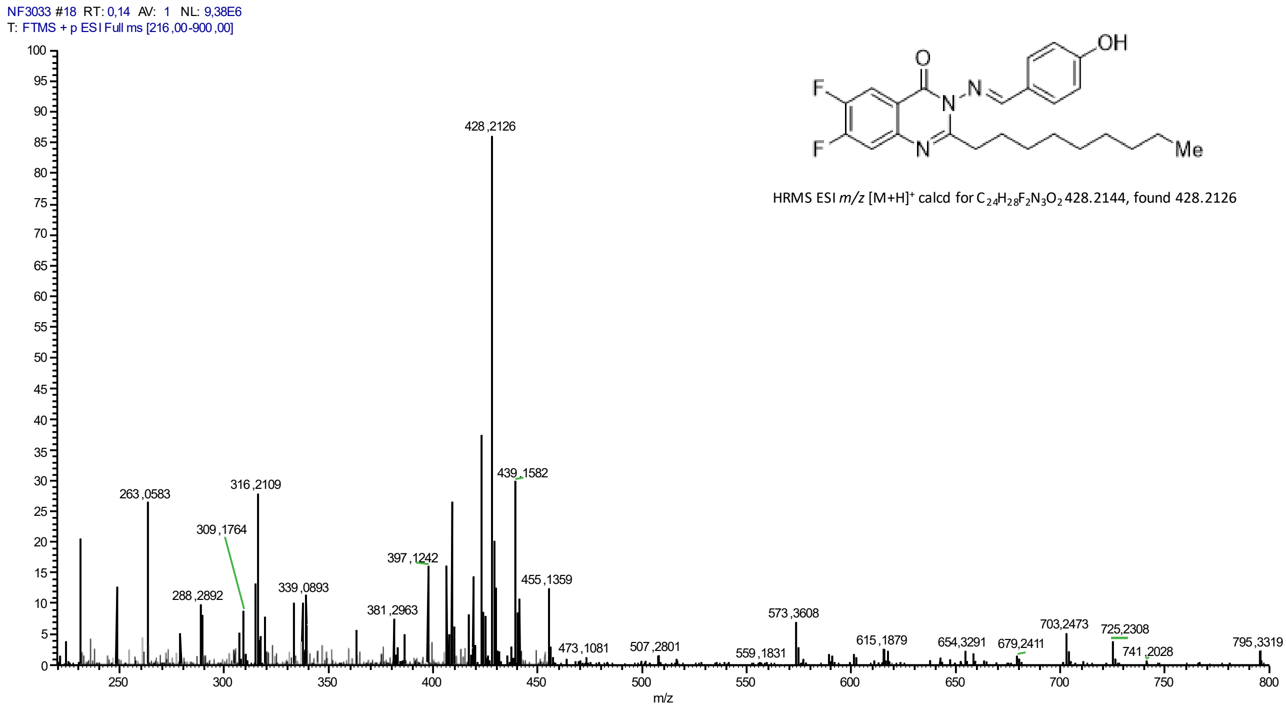

HRMS ESI *m/z* $[M+H]^+$ calcd for $C_{24}H_{28}F_2N_3O_2$ 428.2144, found 428.2126

HRMS spectrum of compound 11a

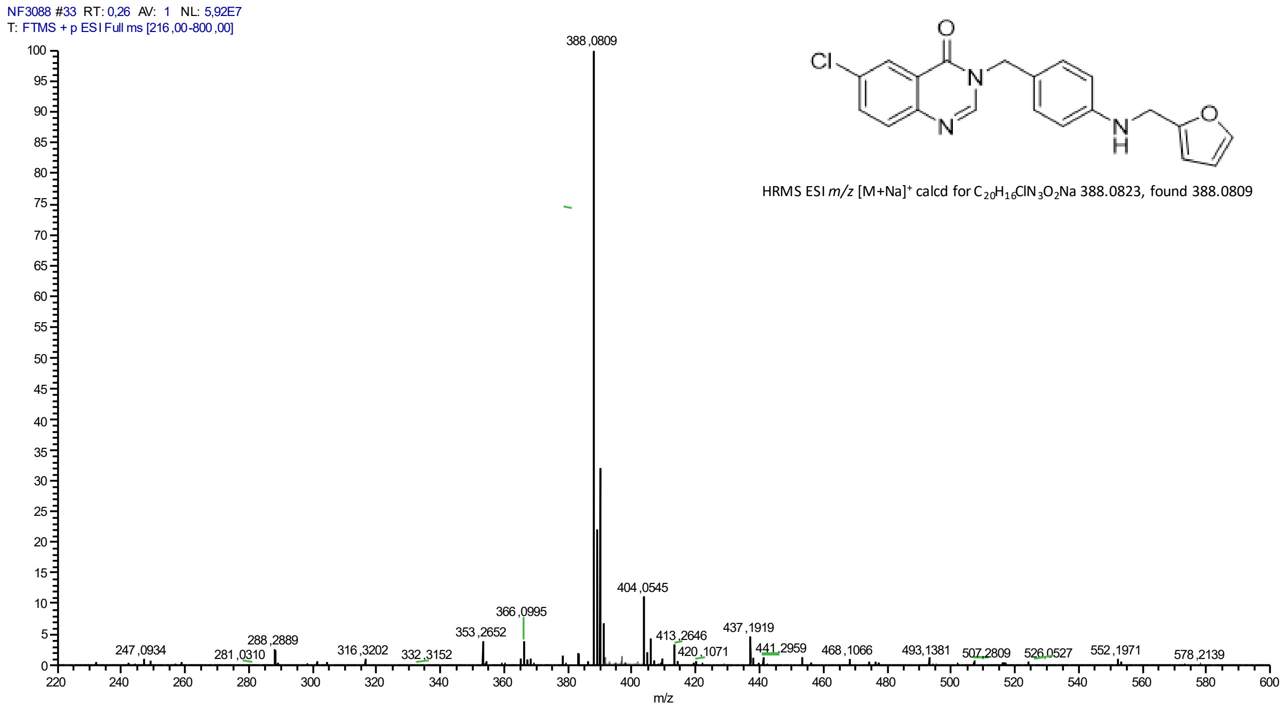

HRMS ESI *m/z* [M+Na]+ calcd for $C_{20}H_{16}ClN_3O_2Na$ 388.0823, found 388.0809

HRMS spectrum of compound 11b

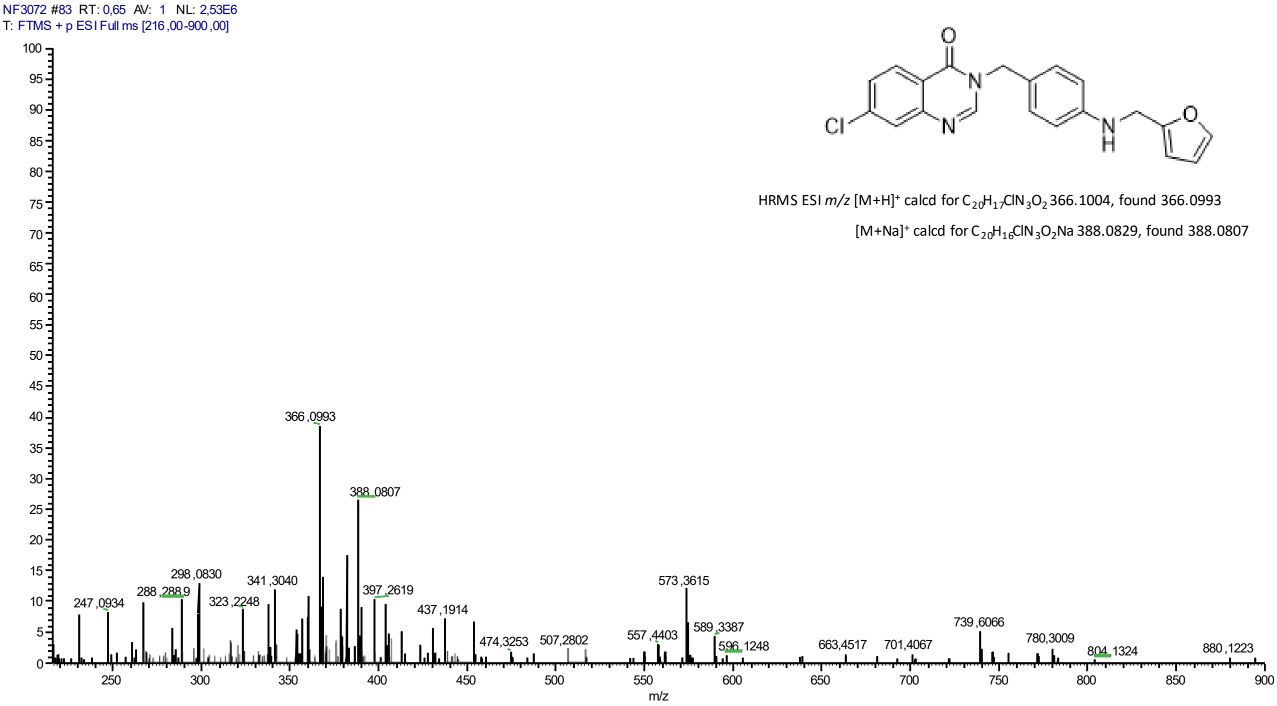

HRMS ESI *m/z* $[M+H]^+$ calcd for $C_{20}H_{17}ClN_3O_2$ 366.1004, found 366.0993

$[M+Na]^+$ calcd for $C_{20}H_{16}ClN_3O_2Na$ 388.0829, found 388.0807

HRMS spectrum of compound 11c

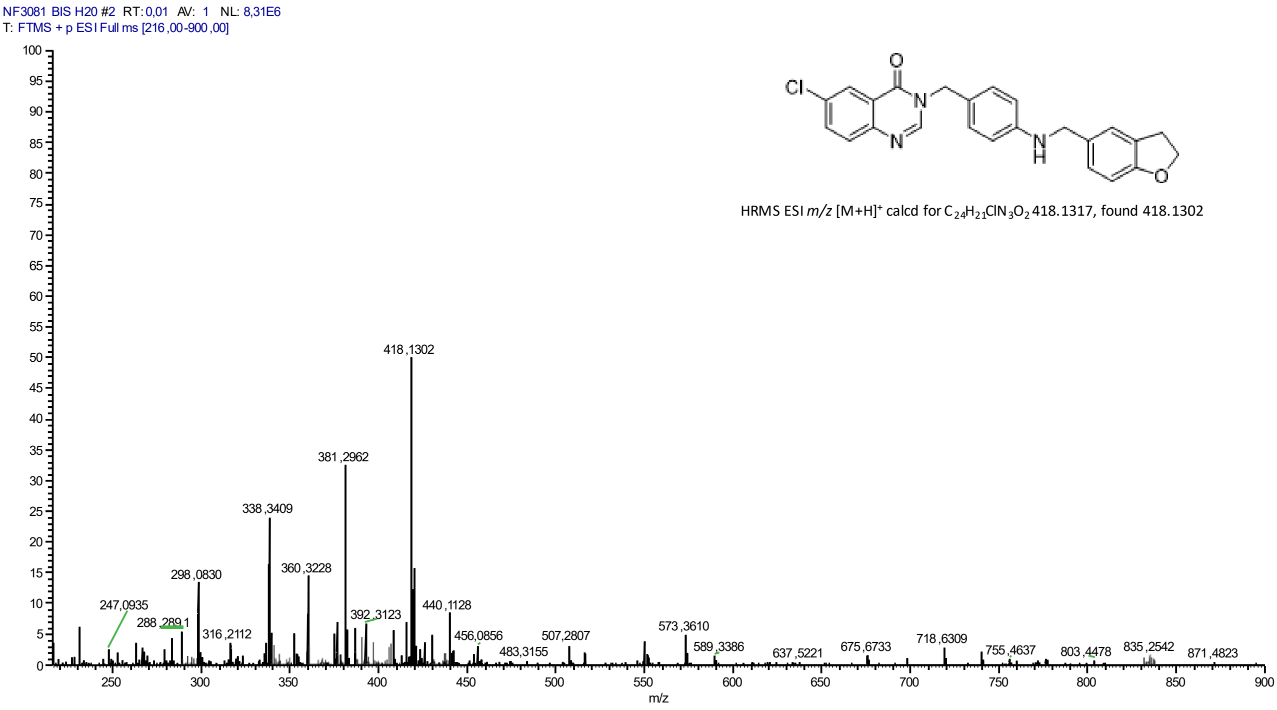

HRMS ESI *m/z* [M+H]+ calcd for $C_{24}H_{21}ClN_3O_2$ 418.1317, found 418.1302

HRMS spectrum of compound 11d

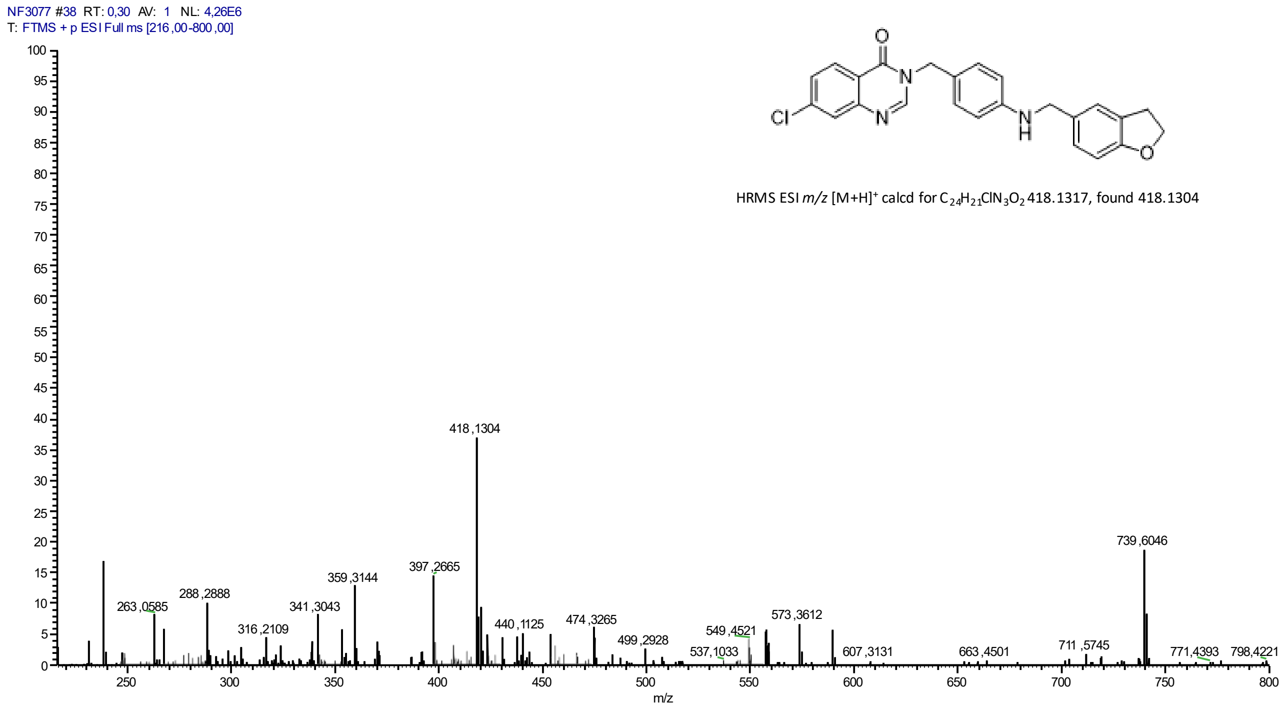

HRMS ESI *m/z* $[M+H]^+$ calcd for $C_{24}H_{21}ClN_3O_2$ 418.1317, found 418.1304
